# Segregation of prokaryotic magnetosomes organelles is driven by treadmilling of a dynamic actin-like MamK filament

**DOI:** 10.1186/s12915-016-0290-1

**Published:** 2016-10-12

**Authors:** Mauricio Toro-Nahuelpan, Frank D. Müller, Stefan Klumpp, Jürgen M. Plitzko, Marc Bramkamp, Dirk Schüler

**Affiliations:** 1 Department of Microbiology, University of Bayreuth, 95447 Bayreuth, Germany; 2 Department of Molecular Structural Biology, Max Planck Institute of Biochemistry, Planegg-Martinsried, Germany; 3 Department Theory and Bio-Systems, Max Planck Institute of Colloids and Interfaces, Potsdam, Germany; 4 Institute for Nonlinear Dynamics, Georg August University Göttingen, Göttingen, Germany; 5 Department of Biology I, Ludwig-Maximilians-University Munich, Planegg-Martinsried, Germany

**Keywords:** Actin, Bacterial cytoskeleton, Magnetosomes, Magnetotactic bacteria, MamK, Organelles, Partitioning, Prokaryote, Segregation

## Abstract

**Background:**

The navigation of magnetotactic bacteria relies on specific intracellular organelles, the magnetosomes, which are membrane-enclosed crystals of magnetite aligned into a linear chain. The magnetosome chain acts as a cellular compass, aligning the cells in the geomagnetic field in order to search for suitable environmental conditions in chemically stratified water columns and sediments. During cytokinesis, magnetosome chains have to be properly positioned, cleaved and separated in order to be evenly passed into daughter cells. In *Magnetospirillum gryphiswaldense*, the assembly of the magnetosome chain is controlled by the actin-like MamK, which polymerizes into cytoskeletal filaments that are connected to magnetosomes through the acidic MamJ protein. MamK filaments were speculated to recruit the magnetosome chain to cellular division sites, thus ensuring equal organelle inheritance. However, the underlying mechanism of magnetic organelle segregation has remained largely unknown.

**Results:**

Here, we performed in vivo time-lapse fluorescence imaging to directly track the intracellular movement and dynamics of magnetosome chains as well as photokinetic and ultrastructural analyses of the actin-like cytoskeletal MamK filament. We show that magnetosome chains undergo rapid intracellular repositioning from the new poles towards midcell into the newborn daughter cells, and the driving force for magnetosomes movement is likely provided by the pole-to-midcell treadmilling growth of MamK filaments. We further discovered that splitting and equipartitioning of magnetosome chains occurs with unexpectedly high accuracy, which depends directly on the dynamics of MamK filaments.

**Conclusion:**

We propose a novel mechanism for prokaryotic organelle segregation that, similar to the type-II bacterial partitioning system of plasmids, relies on the action of cytomotive actin-like filaments together with specific connectors, which transport the magnetosome cargo in a fashion reminiscent of eukaryotic actin-organelle transport and segregation mechanisms.

**Electronic supplementary material:**

The online version of this article (doi:10.1186/s12915-016-0290-1) contains supplementary material, which is available to authorized users.

## Background

In eukaryotes, the transport and segregation of organelles mediated by cytoskeleton and motor proteins are well-studied processes [1–4]. In contrast, it only recently became apparent that bacteria not only possess organelles [5, 6] but also homologs of eukaryotic cytoskeletal proteins such as tubulin, intermediate filaments and several actin families [7–9]. As in eukaryotes, during cell division, the equipartitioning of plasmids, chromosomes and organelles has to be carefully controlled to ensure viability and fitness of the offspring throughout the entire bacterial life cycle. To date, only few examples of organelle or protein cluster segregation in bacteria have been studied in some detail. For example, carboxysomes (protein microcompartments for CO_2_ fixation in cyanobacteria [10]) are linearly spaced by the cell cycle-related ParA protein [11] associated to chromosome-partitioning [12, 13], whereas the segregation of cytoplasmic chemotaxis clusters in *Rhodobacter sphaeroides* also depends on the ParA-like PpfA [14]. However, the fundamental mechanisms of bacterial organelle segregation have remained largely unknown.

A particularly intriguing example of well-ordered prokaryotic organelles are the magnetosomes of magnetotactic bacteria. In the α-proteobacterium *Magnetospirillum gryphiswaldense* MSR-1 (from now on referred to as MSR) magnetosomes are composed of magnetite (Fe_3_O_4_) crystals surrounded by a bilayer membrane, thus resembling eukaryotic organelles [15]. Individual magnetosomes are assembled into a single linear magnetosome chain (MC) that aligns the cell with the earth’s magnetic field. So far, two proteins have been implicated in the assembly of MCs [16], one of which is MamK, a bacterial actin, which polymerizes into a cytoskeletal bundle of two-to-four filaments in vivo and is thought to assemble magnetosomes into a coherent chain [17–19]. MamK from the closely related *Magnetospirillum magneticum* AMB-1 (AMB) was found to form filaments that require an intact ATPase motif for their in vivo dynamics and in vitro disassembly [20, 21]. Furthermore, MamK interacts with MamJ [22, 23], an acidic magnetosome-associated [24] protein thought to attach magnetosomes to the MamK filament in MSR, since *mamJ* deletion caused a collapsed-chain phenotype [25].

To become faithfully divided and segregated during cytokinesis, the MC has to be properly positioned, cleaved and separated against intrachain magnetostatic forces. In MSR, the MC is positioned at midcell, and later localized traversing the division site to be cleaved by unidirectional constriction of the septum [19]. Upon *mamK* deletion MSR cells formed shorter and fragmented MCs [17] that were no longer recruited to the division site [19]. From these observations, it was concluded that newly generated magnetosome sub-chains must undergo a pole-to-midcell translocation into daughter cells, and MamK was hypothesized to mediate this positioning and migration during the MSR cell cycle. However, the pole-to-midcell movement of the MC and the role of MamK in MC positioning are yet to be demonstrated directly and questions such as whether the putative dynamics of MamK filaments may generate the forces required for magnetosome motion and segregation need to be addressed. Overall, the exact mechanism of MC repositioning and segregation (defined as even inheritance of magnetosomes into the offspring) has remained elusive.

Here, by using photokinetics and advanced electron microscopy, we investigated the intracellular dynamics of both the MC and the actin-like MamK filament throughout the cell cycle. We discovered that equipartitioning of MCs occurs with unexpectedly high precision. We found that the MC dynamic pole-to-midcell motion into daughter cells depends directly on the dynamics of MamK filaments, which seem to originate at the cell pole undergoing a treadmilling growth from the pole towards midcell. Furthermore, the observed dynamics of MamJ indicates a transient interaction with MamK. We propose a model where the specific features of MamK filaments dynamics as well as its interplay with MamJ are fundamental for proper MC assembly, precise equipartitioning, pole-to-midcell movement and, ultimately, segregation.

## Results

### Magnetosome chains undergo a rapid and dynamic pole-to-midcell repositioning which becomes impaired by the MamK^D161A^ amino acid exchange

**Fig. 1:**
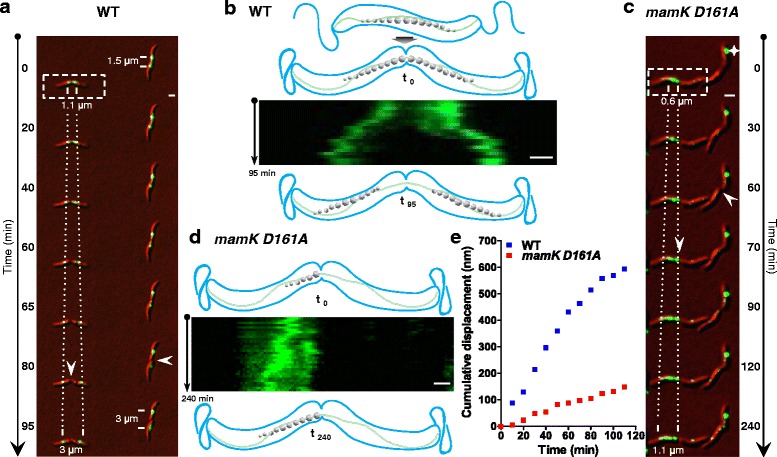
Imaging of magnetosome chain (MC) motion throughout the cell cycle. **a** In vivo time-lapse fluorescence microscopy of MCs by means of MamC-EGFP signal (*green*) in the wildtype (WT). *White bars:* center of EGFP signal position. Distances between *bars* are indicated in the first and last image. *White arrowheads* indicate the frame in which cytokinesis has been completed for each cell. *White dotted lines*: MamC-EGFP signal progression. **b** Kymograph displaying the MamC-EGFP signal (*x*-axis) over the time (*y*-axis) of the WT cell indicated in “A” (*dashed line box*). Schemes above and below depict the septum and MC position at the starting and ending point of the time-lapse. **c** In vivo time-lapse fluorescence microscopy of MCs in the *mamK D161A* strain. *Star:* mispositioning of the chain at cell pole. **d** Kymograph displaying the MamC-EGFP signal (*x*-axis) over the time (*y*-axis) of the *mamK D161A* cell indicated in “C” (*dashed line box*). Schemes above and below depict the septum and MC position at the starting and ending point of the time-lapse. Scale bars: 1 μm. Scale bars of kymographs: 500 nm. **e** MC cumulative displacement as a function of time in the WT (*n* = 24) and *mamK D161A* (*n* = 19) strains. Cumulative displacement was determined from the MamC-EGFP fluorescence signal

To assess the MC localization through the cell cycle, we performed in vivo time-lapse fluorescence imaging of EGFP tagged to MamC (the most abundant magnetosome protein that has been previously used as marker of MC position) [26] in synchronized cells of MSR. In wildtype (WT) cells, single MCs were typically located at midcell (as observed by MamC-EGFP fluorescence), which became evenly partitioned and segregated into daughter cells as the cell cycle progressed (Fig. 1a, Additional file 1: Movie S1). After MC partitioning, the recently divided daughter chains moved apart from the new poles towards midcell into the newborn daughter cells (Fig. 1a, b). MC pole-to-midcell repositioning proceeded with a speed of 18.4 ± 1.1 nm/min (*n* = 87) and was completed after 61.1 ± 4.0 min, i.e., within < 25 % of the MSR doubling time (typically around 240 to 280 min [19, 27]). In addition, the MC repositioning mostly occurred before completion of cytokinesis (Fig. 1a, 80 min-left cell), but in few cells also within the first 30 min after cell division (Fig. 1a, 80 min-right cell).

To study the role of MamK dynamics in magnetosome segregation, we exchanged a conserved aspartate residue by alanine within its ATPase domain (Additional file 2: Figure S1A), yielding the mutant strain *mamK D161A*. Mutation of these conserved residues (D161 or E143 in MamK) abolished ATPase activity and, in turn, the filament dynamics of other actins [9, 21, 28–31].

In contrast to the WT, in vivo time-lapse imaging of *mamK D161A* cells showed that the MC was inherited by only one of the two daughter cells (Fig. 1c, left cell and Additional file 3: Movie S2), suggesting an unequal partitioning of the MC. Further, the *mamK D161A* strain frequently exhibited a mislocalization of the magnetosome signal next to the cell poles (Fig. 1c, 0 min, star). Remarkably, *mamK D161A* did not display MC reposition to the daughter cell center, but instead, after 30 min, a MamC-EFGP signal gradually appeared at the end of the chain (Fig. 1c, d), owing to *de novo* magnetosome synthesis rather than MC pole-to-midcell repositioning. This indicates that the MC was no longer dynamic in the *mamK D161A* strain. Although a late and random displacement of MCs was observed in a minor fraction of *mamK D161A* cells (Additional file 4: Movie S3), MCs were rather static during the previously described asymmetric cell elongation [19].

To quantify the difference in MC movement between WT and *mamK D161A* strains, we determined the cumulative displacement (Fig. 1e) as well as the mean-square displacement (MSD, Additional file 2: Figure S1B) of nascent MCs from cells undergoing division and plotted them as a function of time. In the WT, both parameters exhibited an initial strong increase and entered a plateau at around 90 min, likely because the MC reaches the midcell position where motion is abruptly stopped. The biphasic time dependence of the MC motion was consistent with an initial directed movement followed by restricted mobility at the end [32]. This behavior became even more obvious when the apparent diffusion coefficient (D*) of MCs from WT cells was plotted as a function of time, displaying a continuous increase in D*, consistent with directed rather than diffusive motion, until reaching a maximum value at 90 min lag time, which further underwent a strong and steep decay as observed at 110 min (Additional file 2: Figure S1C). In addition, the cumulative displacement, MSD and D* values were considerably lower in the *mamK D161A* strain (*n* = 19) compared to the WT (*n* = 24), indicating a higher displacement rate and mobility of MCs in WT cells. Furthermore, using MC displacement data, we have determined the velocity (V_MC_) as a function of time (10 min intervals). Strikingly, the *mamK D161A* strain exhibited a low and fairly constant V_MC_ (between 3.6 to 9.3 nm/min; Additional file 2: Figure S1D), whereas WT cells showed an increase of V_MC_ during the first 40 min (up to 17.6 nm/min), which then decreased over time, reaching the V_MC_ levels of the *mamK D161A* mutant (8.3 nm/min; Additional file 2: Figure S1D). The WT V_MC_ behavior matches our hypothesis that MCs are highly mobile for a certain period after cell division (pole-to-midcell transport), and then undergo a strong drop of motion likely correlated with the final midcell positioning. Moreover, V_MC_ values during the period of high mobility were in agreement with the MC speed of WT cells determined above based on the traveled distance (18 nm/min). Altogether, these results demonstrate that MCs underwent a directed movement after cell division, which then becomes restricted upon reaching the final position at midcell, and that the MC motion was severely impaired in *mamK D161A* cells.

### The *mamK D161A* mutation causes a severe mispartitioning of the magnetosome chain

**Fig. 2:**
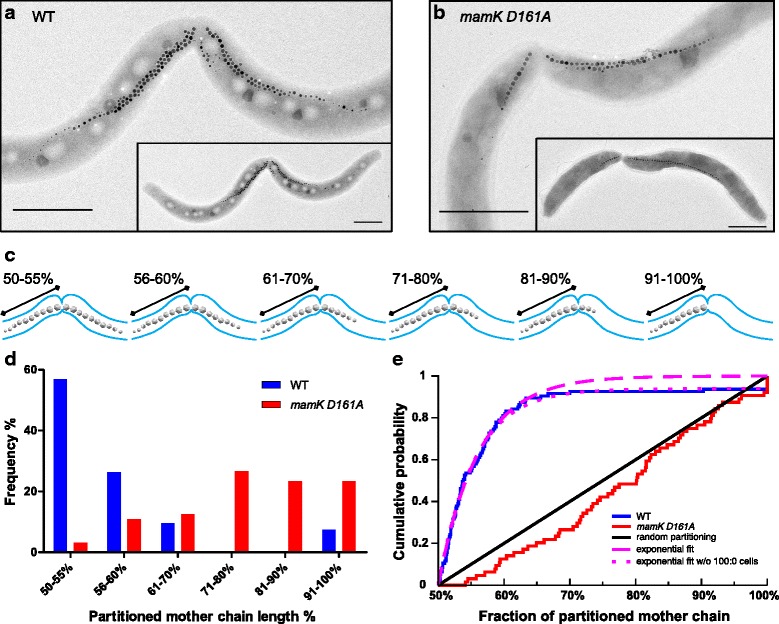
Distribution of magnetosome chain (MC) partitioning. TEM micrographs of (**a**) wildtype (WT) and (**b**) *mamK D161A* cells displaying MC partitioning during cell division. Scale bars: 1 μm. **c** Scheme illustrating putative partitioning scenarios during MC length quantification. **d** Quantification of the partitioned MC length to be inherited by future daughter cells as determined by TEM for the WT (*n* = 95) and *mamK D161A* (*n* = 64) strains. Cells were incubated under 21 % oxygen for 5 h to suppress *de novo* magnetosome synthesis. **e** Cumulative distribution of partitioned MC length data for the WT (*n* = 95) and *mamK D161A* (*n* = 64) strains

Transmission electron microscopy (TEM) revealed that the MC was evenly split in WT cells (Fig. 2a). Strikingly, TEM micrographs of *mamK D161A* cells showed unequal partitioning of the chain, where one daughter cell typically inherited a larger fraction of the MC (Fig. 2b). To further study MC partitioning independently of *de novo* magnetosome synthesis (Fig. 2c), WT and *mamK D161A* strains were incubated for 5 h under 21 % oxygen conditions to suppress magnetite production [33] and to ensure the completion of one entire cell cycle. Again, quantification of the inherited chain length from TEM micrographs showed that WT cells tended to divide the MC into daughter chains of similar lengths (Fig. 2d). Although a minor MC missegregation was observed in few cells (7 %), 83 % of the cells partitioned MCs into equal halves within up to only 10 % fluctuation of the mother chain length. In contrast, in the *mamK D161A* strain, 73 % of the chains were unequally partitioned between a 70/30 % to 100/0 % of the mother chain length, confirming a strongly biased magnetosome segregation for the *mamK D161A* mutant.

If scored for their intracellular position by TEM (Additional file 5: Figure S2A), in *mamK D161A* cells a majority of MCs were located at the pole (Additional file 5: Figure S2B) or adjacent to it (59 %: 37 % and 22 %, respectively; Additional file 5: Figure S2C and S2D). In contrast, WT and ∆*mamK* cell MCs were predominantly positioned at midcell (88 % and 68 %, respectively). Therefore, the increment of MCs found at the poles after cell division, likely caused by absence of MamK dynamics, confirmed the repositioning defect in *mamK D161A* cells resulting in magnetosome missegregation into the offspring.

### Magnetosome chain cleavage occurs with highest possible precision at midchain

To determine whether the MC cleavage is random or driven by specific factors such as directed forces, we analyzed the cumulative distribution of the chain length fractions. Data for the WT strain fitted extremely well (R > 0.99) to the exponential function, F(x) = 1–exp^[–(x–0.5)/λ]^ (Fig. 2e, pink dashed line), corresponding to a distribution of the chain length fraction (x) in which increasingly unequal partitioning is exponentially suppressed. The parameter λ (=0.061), characterizing the accuracy of MC center location, was obtained as 6.1 % of the chain length, which for a chain of 40 magnetosomes corresponds to two magnetosomes. This result remained unchanged if the cell pairs inheriting the MC into one daughter were excluded (Fig. 2e, pink dotted versus dashed lines). Another parameter to characterize the accuracy is the point at which the cumulative distribution is 0.5, that is the median of the distribution. For the fitted exponential function this was 0.542, i.e., 4.2 % deviation from equal partitioning of chain length, again close to one-to-two magnetosomes, suggesting that partitioning proceeded with near-maximal precision.

Furthermore, the MC partitioning distributions of *mamK D161A* cells were compared with a distribution expected for splitting a line (representing an MC) at a random position, which resulted in a linear cumulative distribution (Fig. 2e, red line). A Kolmogorov–Smirnov test of *mamK D161A* strain data was consistent with such random segregation (*P* = 0.14), supporting the MC partitioning defect. On the contrary, WT partitioning data were inconsistent with random segregation (*P* = 5 × 10^–33^).

In addition, as MC partitioning measured by length may not necessarily correlate with the magnetosome number inherited per daughter cell (due to variants such as double chains), we also quantified the latter, obtaining similar results (Additional file 6: Figure S3). These results support the notion that MamK^D161A^ protein residue exchange negatively affects the MC equipartitioning.

Furthermore, the exponential distribution of the deviation from MC equal partitioning in WT cells (Fig. 2e) suggested that the chain center is determined by a balance of an active directed movement and diffusion. As a consequence, the pole-to-midcell movement of the MC after division reflects this active directed movement. We tested this hypothesis *in silico* by simulating the pole-to-midcell movement of the chain after division. The computational model [34] considered an active transport of magnetosomes towards the center of the cell (by MamK filaments dynamics) as well as magnetosome diffusive movements, with magnetic forces considered opposed to such movements. Simulations resulted in an MC repositioning to the cell center upon low and high diffusive movements (Additional file 7: Figure S4A). In the model, active transport is characterized by a linear force-velocity relation and thus by two parameters, a force-free velocity (v_0_) and a stall force (F_s_), against which active transport can work. We hypothesized that defective MC active transport in *mamK D161A* cells could occur by reduction of either parameter and tested both scenarios. In both cases, the chain movement was strongly slowed down (Additional file 7: Figure S4B and S4C). Remarkably, two or more shorter MCs were formed upon reduced F_s_ and high diffusive mobility (Additional file 7: Figure S4C), resembling the phenotype observed experimentally in *mamK D161A* cells.

### Magnetosome concatenation is disturbed in the *mamK D161A* strain

TEM analysis of the *mamK D161A* mutant showed an intermediate phenotype between the WT and ∆*mamK* with respect to MC organization. The WT strain showed only 1 % of cells lacking an MC, whereas 98 % had a single chain (Additional file 8: Figure S5A), consistent with the MC equipartitioning observed before, the remaining 1 % corresponded to cells with double chains. In contrast, 34 % of ∆*mamK* cells had two-to-four fragmented chains, and 50 % of *mamK D161A* cells had between two-to-five chains. Thus, *mamK D161A* cells displayed both WT-like MCs and fragmented chains (Additional file 8: Figure S5B) resembling that of ∆*mamK* [17]. Therefore, it can be hypothesized that a lack of MamK^D161A^ filament dynamics increases MC fragmentation, causing the development of more albeit shorter sub-chains due to magnetosome concatenation deficiency.

**Fig. 3:**
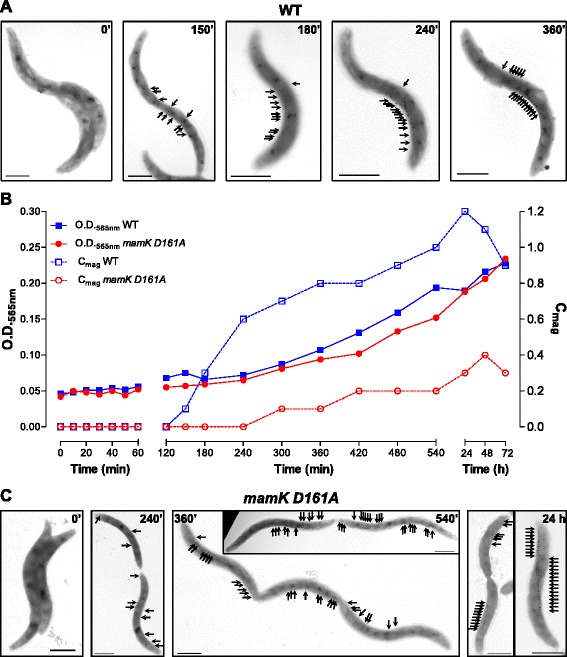
*De novo* magnetite formation kinetics assay. **a** TEM micrographs of non-magnetic wildtype (WT) cells induced for magnetite formation upon addition of 100 μM iron at time zero. Cells are arranged by progression after iron addition. *Black arrows* indicate magnetite crystals position. **b** Growth (OD_565nm_) and magnetic response (C_mag_) for iron-starved non-magnetic WT and *mamK D161A* cells treated with iron at time zero. **c** TEM micrographs of magnetite formation progression in *mamK D161A* cells. Doubling time was determined for WT and *mamK D161A* strains as 4.4 and 4.9 h, respectively. Scale bars: 1 μm

Considering that the replacement of D161A in MamK had an evident impact on chain assembly, we next analyzed whether this mutation also affects *de novo* MC development. To this end, magnetite formation was induced by re-addition of iron to iron-starved non-magnetic cells [25]. The magnetosome synthesis in WT cells was first detected by magnetic response [35] (C_mag_) 150 min post-iron addition, and TEM images revealed small crystallites evenly scattered along the entire cell (Fig. 3a, b). After 180 min, individual crystals began to concatenate into precursory chains, magnetosomes gradually approached each other and moved towards midcell until completion of a WT-like MC at 360 min post-iron addition (Fig. 3a), demonstrating a dynamic MC concatenation as previously described [17, 36]. In contrast, *mamK D161A* cells showed an evident delay in appearance of C_mag_ (Fig. 3b). Moreover, the crystals remained scattered throughout the cell (i.e., spaced for > 50 nm, a distance assumed to be necessary for magnetostatic interaction [17, 37]) even after 540 min post-iron treatment, indicating that magnetosome concatenation was severely affected (Fig. 3c). Only 24 h post-iron addition, *mamK D161A* cells displayed WT-like MCs. In addition, MC uneven segregation and mislocalization of the chains at the cell poles were again observed (Fig. 3c).

### The stabilized MamK^D161A^ filaments cause connected cells

**Fig. 4:**
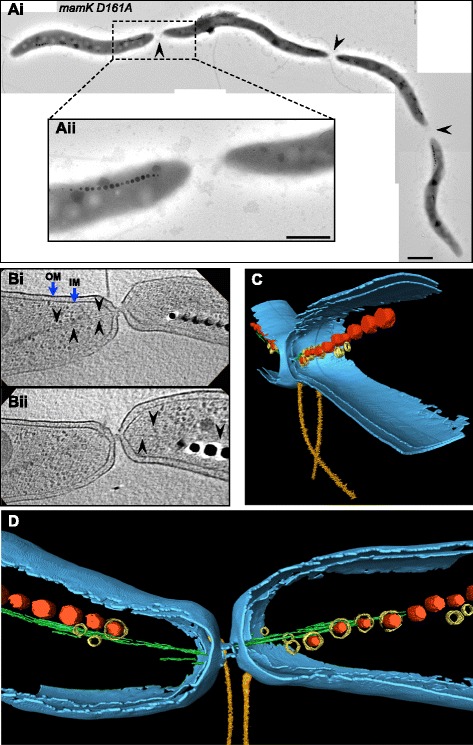
Electron microscopy and cryo-electron tomography (CET) of *mamK D161A* connected cells. **ai** TEM micrograph of four connected cells. *Arrowheads* indicate cell division sites. Scale bar: 1 μm. **aii** Closer view of a cell division site. Scale bar: 500 nm. **b** CET sections of a selected cell division site of three connected cells (*n* = 30). Additional file 11: Figure S8A shows a CET micrograph corresponding to the three connected cells indicating the division site imaged in “b”. *Black arrowheads* denote MamK filaments. *Blue arrows* indicate inner (IM) and outer (OM) membranes. **c**, **d** CET reconstruction of the selected cell division site in “b”. Magnetite crystals (*red*) enclosed by vesicles (*yellow*) and flanked by the MamK filament (*green*). The cellular envelope inner and outer membranes are depicted in blue. Flagella are represented in gold

The *mamK D161A* strain displayed a further perturbed cell separation phenotype. Although cells seemed almost completely divided, they sometimes failed to become fully separated forming up to four joined cells (Fig. 4a and Additional file 9: Figure S6). Fluorescence microscopy of mCherry-MamK^D161A^ filaments in those joined cells showed filaments likely traversing the cell division sites (Additional file 10: Figure S7). Further, cryo-electron tomography (CET) of *mamK D161* division sites from connected cells revealed a tight (35–50 nm) membranous channel connection between the cells (Fig. 4b and Additional file 11: Figure S8A). The MamK^D161A^ filaments appeared to enter and exit the narrow connection of several examined cell division sites (Fig. 4b–d, Additional file 11: Figure S8B–S8F, Additional file 12: Movies S4, Additional file 13: Movies S5, Additional file 14: Movies S6). Consequently, these observations support the hypothesis that the stabilized, non-dynamic MamK^D161A^ filaments could form such rigid structures holding the cells attached and preventing their separation against forces generated by both the divisome complex and the newly forming cell wall.

### MamK filaments exhibit dynamics strongly affected by the D161A residue exchange

**Fig. 5:**
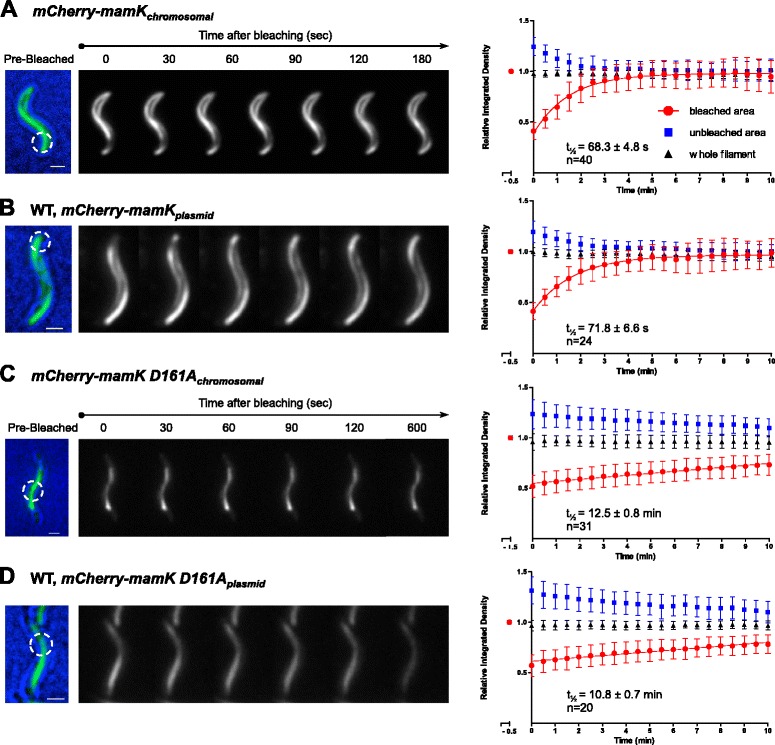
MamK filament dynamics analysis by FRAP. Photobleaching of mCherry-MamK was used to follow the recovery of the fluorescence corresponding to the MamK filament during 10 min. (**a**) mCherry-MamK and (**b**) mCherry-MamK^D161A^ translational fusions expressed from a chromosomal insertion from the native P_*mamAB*_ promoter. (**c**) mCherry-MamK and (**d**) mCherry-MamK^D161A^ translational fusions expressed from a replicative plasmid from the P_*mamAB*_ promoter. The left panels show representative cells for this assay, indicating the selected bleached areas (*white dashed circles*) and fluorescence recovery progression. The pre-bleaching image is a composite of the bright field and fluorescence channel to display subcellular localization. The right panels show the quantification of the MamK filament fluorescence recovery over the time from the corresponding strain. Time point zero was measured immediately after laser pulse. The half-time fluorescence recovery is presented as t_½_ in each plot. Scale bars: 1 μm

All phenotypes of the *mamK D161A* strain presented so far were consistent with a putative lack of MamK^D161A^ filament dynamics. To verify this assumption, the filament dynamics was studied directly by FRAP. In fact, a functional chromosomal translational mCherry-MamK_*chromosomal*_ fusion [38] showed a half-time fluorescence recovery (t_½_) of 68.3 ± 4.8 s (Fig. 5a) after photobleaching. The same *mCherry-mamK*_*plasmid*_ fusion episomally expressed under control of the native *mamK* promoter P_*mamAB*_ in the WT strain (i.e., in presence of the native *mamK* allele) displayed a coherent t_½_ of 71.8 ± 6.6 s (Fig. 5b). We controlled for dark-state-reversal of mCherry fluorophore (described as ~20 s [39]) by fixing the cells with 1 % formaldehyde for 1 h and subsequent evaluation by FRAP, which showed no fluorescence recovery after laser application (Additional file 15: Figure S9A and S9B). These results indicated that mCherry-MamK fluorescence recovery was not due to photoswitching, but reflects a true dynamics of the filaments. In contrast, chromosomal and episomally expressed translational mCherry-MamK^D161A^ fusion showed a consistent t_½_ of 12.5 ± 0.8 and 10.8 ± 0.7 min, respectively (Fig. 5c, d). Therefore, the D161A exchange resulted in a strong decay (10-fold) in dynamics causing stabilized filaments as expected (recovery curves comparison, Additional file 15: Figure S9C-S9F).

**Fig. 6:**
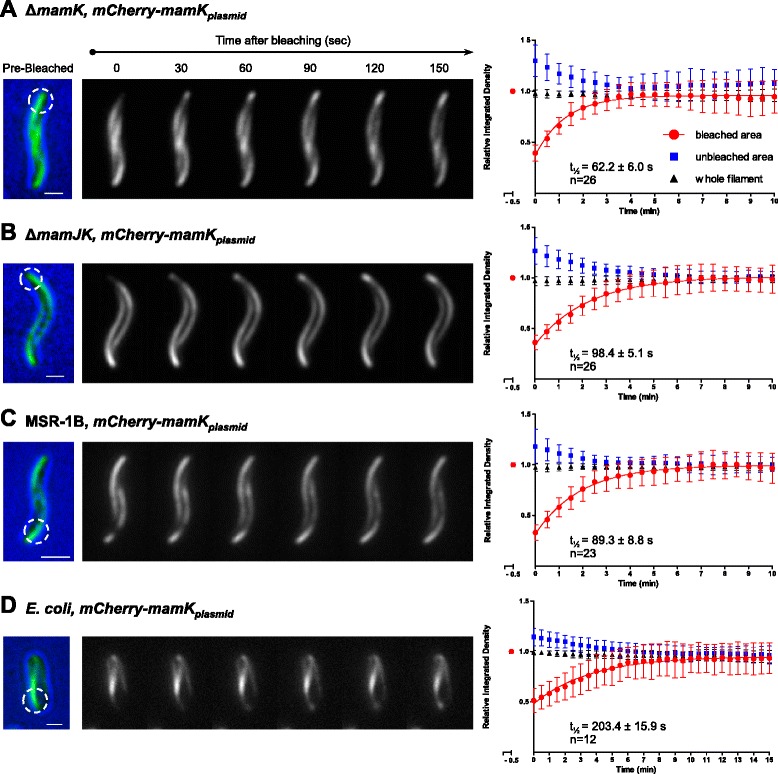
MamK dynamics in different genetic backgrounds. Photobleaching of mCherry-MamK was used to follow the recovery of the fluorescence corresponding to the MamK filament. The mCherry-MamK translational fusion was expressed from a plasmid with the P_*mamAB*_ promoter in the following MSR strains: (**a**) ∆*mamK*, (**b**) ∆*mamJK*, (**c**) MSR-1B, and (**d**) *E. coli*. The left panels show representative cells for this assay, indicating the selected bleached areas (*white dashed circles*) and fluorescence recovery progression. The pre-bleaching image is a composite of the bright field and fluorescence channel to display subcellular localization. The right panels show the quantification of the MamK filament fluorescence recovery over the time from the corresponding strain. Zero time was measured immediately after laser pulse. The half-time fluorescence recovery is presented as t_½_ in each plot. Scale bars: 1 μm

Previously, it was described that MamJ promoted MamK filament turnover in the closely related bacterium AMB [20]. Therefore, we next examined the effect of MamJ on MamK filament dynamics in MSR. First, a ∆*mamK* strain was used as a control to test functionality of the mCherry-MamK_*plasmid*_ fusion (episomally expressed from the P_*mamAB*_ promoter) in the absence of native MamK. mCherry-MamK_*plasmid*_ fluorescence recovery t_½_ was 62.2 ± 6.0 s (Fig. 6a), similar to that of mCherry-MamK_*chromosomal*_ (Fig. 5a). Next, MamK filament dynamics was measured in the ∆*mamJK* background and the non-magnetic mutant MSR-1B (lacking most of the genes comprised within genomic magnetosome island – MAI [40]) resulting in a t_½_ of 98.4 ± 5.1 and 89.3 ± 8.8 s, respectively (Fig. 6b, c). The latter results are statistically different from the t_½_ value described for the mCherry-MamK_*plasmid*_ in the ∆*mamK* strain, as verified by an unpaired Student’s *t*-test (Additional file 16: Figure S10), indicating that MamJ absence has an effect on MamK dynamics. Furthermore, the absence of other MAI-encoded proteins along with MamJ did not promote an additional increase in MamK fluorescence recovery t_½_, excluding a joined epistatic effect of MamJ and other MAI-encoded proteins. In order to evaluate the MamK filament dynamics in the absence of MamJ and all other magnetosome-specific factors from MSR, the *mCherry-mamK* fusion was expressed in *Escherichia coli* under control of the *lac* promoter (Fig. 6d). Indeed, FRAP analysis resulted in a MamK filament recovery t_½_ of 203.4 ± 15.9 s, a three-fold increase compared to its recovery t_½_ in MSR. Despite the MamK dynamics decreasing considerably in the absence of MamJ or any MAI-encoded protein, the fluorescence recovery was always completely restored to 1.0 during the experiment. This observation can be interpreted such that the calculated mobility fraction for MamK molecules is a 100 % in all the genetic backgrounds tested, suggesting that MamK is highly dynamic and dependent only partially on the presence of MamJ.

### MamK filaments appear to originate at the cell poles and undergo treadmilling growth

**Fig. 7:**
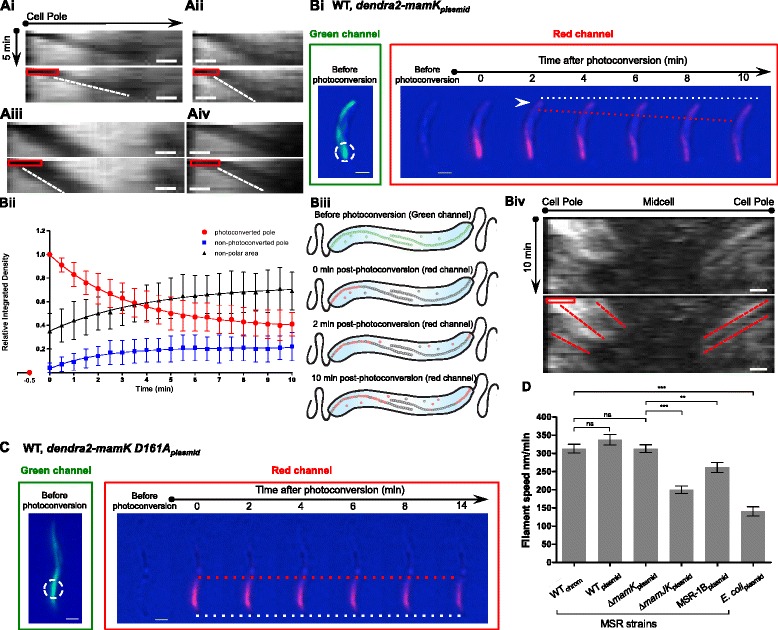
MamK filament growth behavior analysis by FRAP and photoconversion. **a** Kymographs displaying fluorescence signal intensity (*x*-axis) of bleached MamK filaments over the time (*y*-axis). mCherry-MamK fusion expressed from a plasmid (P_*mamAB*_ promoter) in MSR: (**ai**) wildtype (WT), (**aii**) ∆*mamK*, (**aiii**) ∆*mamJK*, and (**aiv**) MSR-1B. The corresponding duplicated kymograph indicates bleaching time/area (*red box*) and filament fluorescent signal progression (*white dashed line*). The bleach-marked filaments were followed for 5 min (imaging every 30 s). **bi** Photoconversion of Dendra2-MamK expressed in WT cells from a plasmid (P_*mamAB*_ promoter). *Green* channel: MamK filament prior to photoconversion. *Red* channel: photoconverted protein after a 405 nm laser pulse. *White dashed circle*: photoconverted area. *White dashed lines* act a reference point. *Red dashed lines*: filament growth progression. *Arrow* indicates appearance of MamK signal at the cell pole. A total of 91 % of cells (*n* = 54) showed polar appearance of the photoconverted protein. **bii** Cartoon illustrating intracellular Dendra2-MamK dynamics. After the laser pulse (left pole), the filament and Dendra2-MamK free subunits are photoconverted (*green*-to-*red*). Freely diffusible Dendra2-MamK subunits migrate and incorporate at the right cell pole, where MamK filaments originate. **biii** Kymograph of a cell (Additional file 18: Figure S11E) displaying intracellular localization of the red photoconverted signal (*x-*axis) over time (*y-*axis). The corresponding duplicated kymograph below indicates the photoconverted time/area (*red box*) and MamK filament fluorescent signal appearance/progression (*red dashed lines*). **biv** Quantification of photoconverted Dendra2-MamK signal in WT cells (*n* = 21). **c** Photoconversion of Dendra2-MamK^D161A^ as in “bi”. Lack of dynamics was observed in 100 % of cells (*n* = 16) (see Additional file 18: Figure S11D for quantitative data). **d** MamK filament treadmilling speed per strain quantified from mCherry-MamK photobleaching data. An unpaired Student’s *t*-test was performed. * Significant: *P* = 0.01 to 0.05. ** Very significant *P* = 0.001 to 0.01. *** Extremely significant *P* < 0.001. ns: not significant.

The observed mCherry-MamK filaments usually generated a fluorescence signal whose intensity was higher at the cell poles and gradually decreased towards midcell along the filament length (Fig. 5a, b and Fig. 6a, c). In contrast, mCherry-MamK^D161A^ filaments appeared slightly shorter and displayed an even fluorescence intensity that was not increased at the poles (Fig. 5c, d). We generated kymographs from mCherry-MamK filaments bleached at the cell pole of four MSR strains: WT, ∆*mamK*, ∆*mamJK*, and MSR-1B (Fig. 7ai–aiv, respectively, Additional file 17: Movie S7). Remarkably, these kymographs showed that the fluorescence began to recover at the pole itself and migrated towards midcell in the four analyzed strains. Moreover, when an internal section of a MamK filament bundle was photobleached (Additional file 17: Movie S7), the bleached zone moved towards the opposite pole from where the filaments actually originated. Additionally, *mCherry-mamK*_*chromosomal*_ expressed from the native locus in MSR also displayed filaments originating at the poles (Additional file 18: Figure S11Ai and S11Aii), indicating that this phenomenon was not caused by a MamK overdose in strains expressing the fusion from multiple-copy plasmids. Therefore, insertion of subunits at the cell pole likely pushes the subunits in the filaments towards midcell, suggesting a treadmilling behavior. Similar results were obtained in *E. coli* (Additional file 18: Figure S11Bi and S11Bii), suggesting that the assumed treadmilling growth is an inherent property of MamK and does not require the presence of additional magnetosome-specific factors.

To further substantiate the polar origin of the filaments, MamK was tagged with the Dendra2 fluorophore capable of irreversible green-to-red photoconversion upon blue or UV-light excitation [41], allowing tracking of the newly activated red form and, consequently, protein dynamics [42]. The Dendra2-MamK filament was visualized in the green channel to select the area to be photoconverted. After application of a 405 nm laser pulse to the Dendra2-MamK filament at a cell pole, the photoconverted protein was monitored through the red channel. A few seconds after photoconversion of the filament at one cell pole, the Dendra2-MamK red signal was detected at the opposite pole as observed qualitatively (Fig. 7bi and Additional file 18: Figure S11C) and quantitatively (Fig. 7bii). Quantification of the fluorescent signal after photoconversion indicated that, while the photoconverted pole (red channel) signal intensity decayed over time, the signal intensity of both the non-photoconverted pole and non-polar area increased. The half-time of the red fluorescent signal decay at the photoconverted pole (187.8 ± 30.6 s, n = 21) and corresponding increase at the non-photoconverted pole (147.9 ± 27.0 s) shared a comparable timing. However, the half-time signal increment of the non-polar area lagged behind (381.7 ± 94.5 s), proving that the signal appearance in the non-photoconverted pole occurred faster than the suggested MamK filament treadmilling. In addition, the occurrence of red photoconverted signal at the opposite (non-photoconverted) pole was faster than the putative MamK filament growth time (described below) to reach the opposite cell pole. Thus, by the last two observations it can be ruled out that the signal appearance at the non-photoconverted pole is due to MamK filaments growing and reaching the opposite (i.e., non-photoconverted) pole.

We also generated kymographs of the subcellular progression of the photoconverted signal that display the MamK filament successive nucleation events in the non-photoconverted pole and further midcell displacement due to filament growth (red dashed lines – right side – in Fig. 7biii and biv; Additional file 18: Figure S11D). Remarkably, the filament nucleation event could also be seen at the photoconverted pole after a certain time (red dashed lines – left side – in Fig. 7biv and Additional file 18: Figure S11D), supporting the concomitant bipolar filament nucleation theory. Furthermore, the gradual disappearance of the fluorescence intensity along with filament growth suggests a gradual depolymerization of the filament at the opposite old growing end. Signal disappearance was not due to photobleaching during imaging since new signals appeared nucleating at the same cell pole simultaneously (red dashed lines in Fig. 7biv and Additional file 18: Figure S11D). Then, the photoconversion data set further supports the incorporation of subunits to the filaments occurring at the poles. In this manner, MamK filament polymerization proceeded growing by the cell poles and migrating towards midcell, likely depolymerizing gradually at the opposite old end. Assuming that the laser action at the cell pole photoconverted both (1) part of the newly growing Dendra2-MamK filament and (2) a pool of existing free Dendra2-MamK monomers, the latter could rapidly diffuse to the non-photoconverted pole to be integrated into the MamK filaments.

Photoconversion of Dendra2-MamK^D161A^, however, resulted in static, non-dynamic filaments lacking cell pole origination (Fig. 7c). In agreement with this, no signal intensity changes were detected upon quantification of different cellular zones (Additional file 18: Figure S11E). In order to further confirm this result, we generated a similar mutation of a conserved glutamate by alanine in the ATPase domain of MamK, MamK^E143A^, also predicted to cause impairment of the ATPase activity and thus filament dynamics [20, 21, 28]. Dendra2-MamK^E143A^ photoconversion at the cell pole also resulted in static filaments (Additional file 18: Figure S11F). Accordingly, photoconversion experiments also support the notion of MamK filament cell pole origination, treadmilling growth, and lack of dynamics of MamK^D161A^ filaments.

We next calculated the speed of MamK filament putative treadmilling from the bleached filaments dataset. MSR cells expressing mCherry-MamK_*chromosomal*_ had a treadmilling speed of 313 ± 12.1 nm/min (n = 36), significantly similar to that of mCherry-MamK_*plasmid*_ in MSR WT (337 ± 14.6 nm/min, *n* = 27) and ∆*mamK* backgrounds (313 ± 10.6 nm/min, *n* = 28) (Fig. 7d; Additional file 18: Figure S11G and S11H). Strikingly, the filament treadmilling speed was considerably higher (17-fold) than the MC motion (18 nm/min). Furthermore, MamJ stimulation of MamK filament dynamics was also reflected in the putative treadmilling growth, since absence of *mamJ* as well as the MAI decreased the treadmilling speed to 200 (*n* = 20) and 261 (*n* = 28) nm/min, respectively (Fig. 7d), which are statistically different from the filament speed obtained in the ∆*mamK* strain. To verify the influence of MamJ absence in the MamK filament treadmilling speed and the corresponding fluorescence recovery t_½_, we generated a correlation plot of treadmilling speed versus fluorescence recovery t_½_ using data of all analyzed MSR strains (Additional file 18: Figure S11I). The data fitted to a linear regression and the Pearson correlation coefficient (*r*) showed a significant (*P* = 0.01 to 0.05) negative linear relationship (*r* = –0.9097) between the decrease in filament treadmilling speed and the increment of fluorescence recovery t_½_ upon absence of MamJ. Therefore, it can be suggested that the mCherry-MamK filament fluorescence recovery is likely due to treadmilling growth, which in turn is stimulated by MamJ presence.

### MamJ turnover is higher than that of MamK

**Fig. 8:**
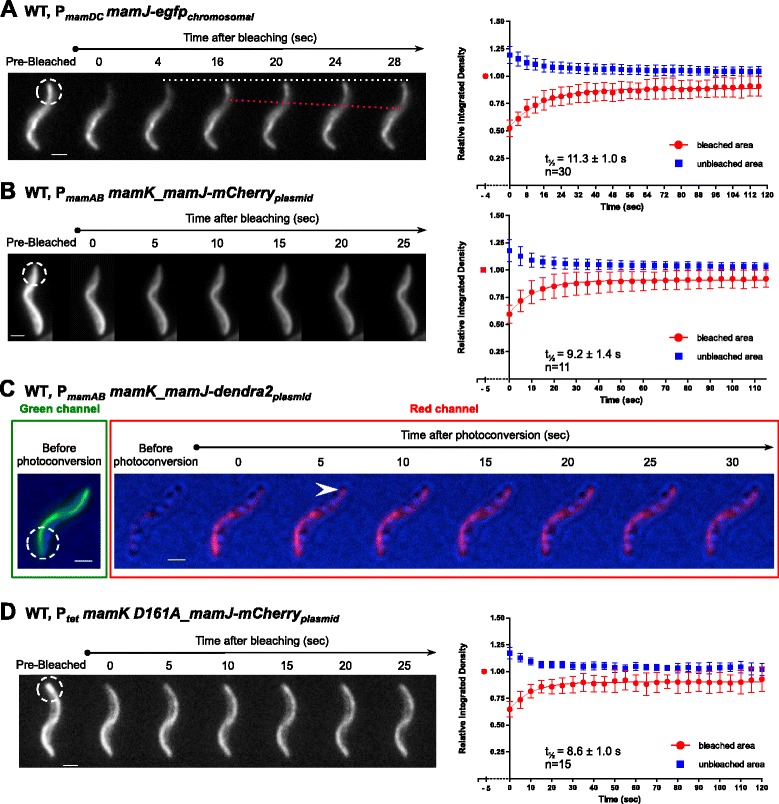
Analysis of MamJ dynamics. Photobleaching of EGFP and mCherry was used to follow the recovery of the fluorescence corresponding to MamJ. **a** MamJ-EGFP and **b** MamJ-mCherry translational fusions expressed from a Tn5-based chromosomal insertion and a replicative vector, respectively. *White dashed circles* indicate bleached areas. *White dashed line* acts a reference point. *Red dashed line* indicates signal progression. The right panels show the quantification of the MamJ fluorescence recovery over the time from the corresponding strain. Zero time was measured immediately after laser pulse. The half-time fluorescence recovery is presented as t_½_ in each plot. **c** MamJ-Dendra2 photoconversion upon *mamK* co-expression in MSR wildtype (WT). *Arrow* indicates appearance of MamJ signal at the cell pole. A 67 % of analyzed cells (*n* = 27) showed a putative polar appearance of the photoconverted protein. *Green* channel displays the filament prior to photoconversion. *Red* channel shows photoconverted the protein after a 405 nm laser pulse application. **d** Photobleaching of MamJ-mCherry upon *mamK D161A* co-expression in MSR WT. The *white dashed circle* indicates the bleached area while the right panels show the quantification of the MamJ fluorescence recovery over time. The half-time fluorescence recovery is presented as t_½_ in each plot. Scale bars: 1 μm

To analyze a direct connection between the MamK filament dynamics and magnetosome motion, the MamK-interacting and magnetosome-associated MamJ protein dynamics was examined. FRAP of two MamJ C-terminal tagged fusions (EGFP and mCherry, expressed either chromosomally or episomally) using two laser lines showed a consistent recovery t_½_ of 11.3 ± 1.0 and 9.2 ± 1.4 s, respectively (Fig. 8a and b; Additional file 19: Figure S12A), determining that MamJ turnover is faster compared to MamK recovery t_½_. Notably, the fluorescence recovery of MamJ-EGFP reached 100 % at approximately 5 min post-bleaching (not shown), suggesting a 100 % fraction mobility for the MamJ protein.

Photoconversion of MamJ-Dendra2 was employed to investigate whether MamJ, like MamK, originates at the cell poles. MamJ-Dendra2 overexpressed in MSR WT showed a week filamentous signal compared to the cytoplasmic signal (Additional file 19: Figure S12B), indicating a limited availability of MamK molecules to interact with. Photoconversion of the poles in this strain did not function properly as the tracking of red photoconverted proteins was difficult due to the fast MamJ mobility (arrow, Additional file 19: Figure S12B). Therefore, we overexpressed MamK together with MamJ-Dendra2 in MSR WT and ∆*mamJK* (Fig. 8c and Additional file 19: Figure S12C, respectively), which showed an improved MamJ filamentous localization where photoconversion suggested a rapid polar appearance of the signal after photoconversion of the opposite cell pole (arrows, Fig. 8c and Additional file 19: Figure S12C; dashed lines, Fig. 8a). However, the overly fast MamJ dynamics prevented unambiguous interpretation of its polar origination.

We further evaluated whether the MamK dynamic status affects MamJ turnover, and specifically whether the lack of MamK^D161A^ dynamics has an influence on the dynamics of MamJ. Thus, this assay could provide further proof for the short-lived interaction between MamJ and MamK. For this, MamJ-mCherry dynamics was analyzed upon co-expression of *mamK D161A* in the MSR WT, ∆*mamK*, and ∆*mamJK* strains. It is important to note that MamK^D161A^ is a negative trans-dominant mutation in the WT background as shown in Fig. 5d. FRAP analysis of MamJ-mCherry showed a consistent recovery t_½_ of 8.6 s (±1.0), 10.6 s (±2.0), and 12.2 s (± 1.7) for the WT (Fig. 8d), ∆*mamK*, and ∆*mamJK* strains (Additional file 19: Figure S12D and S12E), respectively. The latter values of recovery t_½_ are similar to that of the MamJ dynamics in the presence of the *mamK* WT gene (from Fig. 8a, b and Additional file 19: Figure S12A). In addition, photoconversion assessment of MamJ-Dendra2 upon co-expression of *mamK D161A* gene in ∆*mamJK* (Additional file 19: Figure S12F) and MSR WT (not shown) strains revealed a fast MamJ motion throughout the cell after the photoconversion event.

We controlled for dark-state-reversal of all used fluorophores fused to MamJ as mentioned above, revealing no fluorescence recovery or dynamics in fixed cells (Additional file 20: Figure S13A–S13D). To corroborate that *mamJ* and *mamK* were properly expressed, we checked for complementation of the ∆*mamJK* strain that no longer displays a linear MC, but instead has a phenotype that resembles that of ∆*mamJ*, i.e., clustered magnetosomes. TEM micrographs indicated that cells were rescued by either of these constructs as the agglomerated magnetosomes in ∆*mamJK* were reconstituted into linear MC (Additional file 21: Figure S14B–S14D), which was also properly segregated upon presence of *mamK* (Additional file 21: Figure S14B and S14D). It is important to note that, for reconstitution of a WT-like MC and proper mispartitioning, both *mamJ* and *mamK* must be present. Although non-complemented cells exhibiting clustered magnetosomes can still be observed (Additional file 21: Figure S14B–S14D), this is absolutely within expectations due to the fact that we mostly used replicative plasmid derivatives of the pBBR1 vector. It has previously been reported that *gfp* expression from pBBR1 plasmid in MSR causes only up to 26 % of cells to fluoresce [43]. Therefore, the observed cells harboring clustered magnetosomes are likely unable to express the fusion of interest, but not due to a lack of functionality of the fusion itself. As a matter of fact, the complementation is such that the chains are even correctly localized at midcell and further properly partitioned (Additional file 21: Figure S14B and S14D), confirming that MamJ as well as MamK must be strictly present. Finally, when co-expressing a *mamJ* fusion together with a *mamK D161A* gene in ∆*mamJK* cells, the agglomerated magnetosomes were reconstituted into linear MCs. Additionally, the phenotypes of MC mispartitioning and polar retention in single cells were also commonly observed (Additional file 21: Figure S14E and S14F).

Collectively, these findings reveal that the MamJ turnover does not depend on MamK dynamics and that the rapid MamJ turnover upon the presence of static MamK^D161A^ filaments indicates a putative transient interaction with MamK, where MamJ might constantly bind and dissociate from MamK filaments.

## Discussion

By direct imaging of MCs in live cells, we showed that MCs are precisely partitioned into equal halves and, indeed, undergo a dynamic pole-to-midcell repositioning. Furthermore, MC equipartitioning and motion relied directly on a dynamic MamK filament with an intact ATPase domain. In fact, despite a minor missegregated fraction, in most cells, MCs were precisely partitioned typically into equal halves. The precision with which the MC center was placed at the septum roughly corresponded to the dimension of two magnetosomes. Since (1) MCs splitting must naturally occur between two magnetosomes and (2) the maximal precision to locate the chain center is the size of one magnetosome, our data suggest that equipartitioning of MCs take places with the highest possible accuracy.

Our data support the notion that MSR MamK filaments originate and grow from the cell poles likely undergoing treadmilling, where incorporation of MamK subunits at the cell poles pushes the subunits in the filament towards midcell equivalent to exerting a “treadmilling against a wall”. In agreement with this, previous CET observations showed that MSR MamK filament bundles appeared to end nearby the cell poles [19], inferring a putative filament polar origin. The results of photoconversion experiments of MamK filaments also support this hypothesis, as the polar photoconverted filament area moved towards midcell together with the appearance of a new signal at the non-photoconverted cell pole. Moreover, the MSR MamK filament likely disassembles gradually at the opposite growing end, since the earliest incorporated monomers within the filament might undergo ATPase activity causing depolymerization. In accordance with this, MamK filaments exhibited a stronger fluorescent signal at the cell poles (i.e., at their origin) that decreased along the filament length, in contrast to the stable MamK^D161A^ filaments. In agreement with our observations in MSR, in the related AMB, an intact ATPase activity was also a prerequisite for disassembly of polymeric MamK in vivo and in vitro [20, 21, 28]. Additionally, in our study, the polar photoconverted filaments exhibited a dilution of the signal over time, which may be either due to photobleaching during imaging or filament turnover, assuming that MamK forms bundles of overlapping polymers, non-photoconverted oligomers could be substituted along the filaments by lateral association-dissociation, as suggested for other bacterial actin filaments such as AlfA [29] and MreB [44]. Thus, MSR MamK as well as other bacterial actins are proposed to form bundles of dynamic polymers that experience turnover and/or treadmilling [9, 28, 29, 44, 45], the latter also a feature of eukaryotic actins [46].

**Fig. 9:**
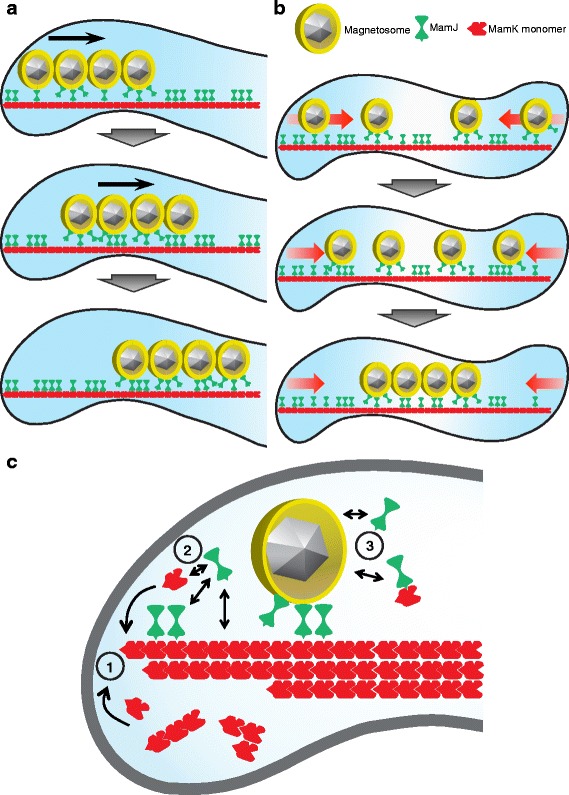
MamK-MamJ tracks drive magnetosome chain concatenation and repositioning. **a** Progression of a recently divided MC undergoing motion from the pole towards midcell conducted by the MamK-MamJ tracks due to the MamK incorporation to the filament at the cell poles and further treadmilling growth. **b** Stepwise magnetosome concatenation into a linear chain driven by MamK filament treadmilling and its interaction with MamJ. **c** Models depicted in “a” and “b” show only one filament. However, it is known that MamK forms filament bundles that flank the MC. Steps 1 to 3 represent the putative dynamics and interactions of MamK, MamJ, and magnetosomes during MC assembly and/or transport

Based on our observations, we propose a model where magnetosomes are actively transported by a mechanism dependent on MamK filaments and their interaction with the magnetosome-associated MamJ [24, 25]. Considering that MamK filaments eventually originate from the new cell poles of recently divided cells, the suggested constant generation and breakage of interactions between MamK-MamJ and magnetosomes-MamJ would direct magnetosome motion from the poles of newborn cells towards midcell (Fig. 9a). Notably, this implies that MamK-MamJ complexes move faster than MCs, inferring that the intracellular directed motion of large objects like MCs across the viscous cytoplasm requires elevated forces and energy, as motion is disproportionally constrained with object size increment [47]. It has been shown that eukaryotic actin polymerization can generate forces of approximately 1 pN [48, 49], therefore, the fast-moving actin-like MamK filaments and the suggested interplay with MamJ might provide sufficient forces for magnetosome transport towards midcell against the cytoplasmic friction. In the same fashion, MSR MamK filament growth features could explain why magnetosomes become concatenated into a linear chain specifically located at midcell (Fig. 9b). Independent of the magnetostatic forces, an external force of approximately 1 pN is indispensable to form a single midcell positioned MC in MSR [34], matching the hypothetical force that could be exerted by MamK. Thus, stochastically nucleated crystals in the magnetosome vesicles could be guided towards midcell by MamK filaments and aided by magnetic interactions between adjacent magnetosomes generating a single midcell localized MC. However, the mechanism might be even more complex assuming not a single filament but a bundle of two to four filaments (Fig. 9c), as previously shown [17], implying that these filaments could be laterally connected. This is also based on the fact that bleached areas of a bundle of MamK filaments seem to move coordinately, making tracking of the treadmilling growth front line possible. On the same matter, a related study by Abreu et al. [50] proposed a model for MamK lateral interaction with a second MamK-like homolog. In addition, each single filament could have different polymerization states; thus, the older part of the filament segments could undergo ATPase activity and disassemble. The filament disassembly also seems to be coordinated as observed by photoconversion, which could be explained due to a coordinated growth rate of many single filaments and their putative lateral interactions helping to synchronize bundle growth. In this scenario, MamK molecules may be incorporated by the cell poles (Fig. 9c, Step 1), whereas MamJ could interact transiently with either free MamK or MamK units incorporated in the filament (Fig. 9c, Step 2), and also associating-dissociating from magnetosomes (Fig. 9c, Step 3). Although there is strong evidence endorsing a direct MamK-MamJ interaction [22, 23], the presence of a third party intermediary protein cannot be discarded, which could have an important role in the interplay of these two proteins regulating their dynamics. However, our data suggest that a dynamic MamK filament and its interplay with MamJ are fundamental for proper MC assembly and intracellular motion in MSR.

MamK possesses a recovery t_½_ comparable to eukaryotic actin (~13 s) [51] and several bacterial actins involved in plasmid segregation such as ParM [28], AlfA (~45 s) [29], and Alp7 [9]. Strikingly, MamK from MSR has a recovery t_½_ 10-fold faster with regards to AMB MamK (~11 min) [20], which instead resembles that of MSR MamK^D161A^ stable filaments. Furthermore, MamJ is essential for AMB MamK dynamics as its deletion caused absolutely static filaments [20]. Although we observed that MamJ enhances the MSR MamK filament dynamics, namely treadmilling, no additional magnetosome-specific factors are necessary for its dynamics as it was self-sustained, exhibiting a fast fluorescence recovery rate, 100 % of mobility fraction, and treadmilling growth in all tested genetic backgrounds. These phenotypic differences between MSR and AMB might be attributed to species-specific characteristics previously reported. For instance, while MSR forms a single or double compact coherent MC located at midcell, AMB generates a long and continuous MC that may traverse the entire cell. However, and remarkably, MCs of AMB seem to be fragmented, as mature crystals are often interspersed with empty magnetosome vesicles. Second, deletion of *mamK* in MSR caused formation of shorter and segmented MCs [17], but not scattered magnetosomes as in AMB [18]. A third and more striking difference resulted from the deletion of *mamJ*, which in MSR caused loss of the linear MC, forming clustered magnetosomes instead [25], whereas in AMB, the same deletion had only a minor phenotype in MC organization as the chain displayed small gaps lacking magnetosome vesicles [20]. Furthermore, AMB contains a single *mamK*-like [50, 52] and *mamJ*-like [20] homolog, whose independent deletion had no major influence in MC arrangement. These examples reveal incongruences between identical mutants of MSR and AMB, conferring unique processes of chain assembly.

Replacement of key residues within the ATPase active site of MamK (MamK^D161A^) also generated connected cells in which the separation of individual cells was impaired, reminiscent to previous observations where equivalent mutations in bacterial actins [9, 29] or tubulin-like proteins [53] and overexpression of intermediate filament-like proteins [54] resulted in comparable phenotypes. Likely, the growing septum requires higher constricting forces to split these rigid filaments, which eventually might break due to local filament disassembly or turnover.

Following from our study, an intriguing question remains to be answered: what are the cellular factors causing the MamK filaments to first originate at the cell poles and to then position the MC at midcell? To address this, obvious candidates such as other cytoskeletal structures including the actin-like MreB (with functions in cell shape and growth) [55] or the tubulin-like FtsZ (forming the Z-ring and organizing the divisome) [7] need to be investigated with respect to their influence on MC assembly, positioning, and segregation through the MSR cell cycle. In addition, an assessment of MamK protein surfaces involved in direct contact with either MamJ or with other MamK proteins (by putative lateral filaments interaction) is needed to further understand the MamK dynamics. Further, current cutting edge microscopy techniques such as single molecule tracking at nanometer resolution could serve to meticulously study the intracellular motion of MamK.

To date, only few other prokaryotic organelles were studied with respect to their intracellular distribution and segregation. For instance, the intracellular distribution of carboxysomes was found to be directed by the cell cycle-related ParA [11]. The type-I (ParA-based) partitioning system encodes a Walker-type ATPase (ParA), a DNA-binding protein (ParB), and a cis-acting centromeric site, *parS* [12, 13, 56]. Thus, ParA is associated to chromosome-partitioning forming a protein gradient based on monomer-dimer states [57, 58] as well as specific and unspecific interactions with ParB and DNA, respectively. Furthermore, segregation of cytoplasmic chemotactic clusters in *R. sphaeroides* was also attributed to the ParA-like PpfA [14]. These two examples are based on a type-I system somewhat reminiscent of chromosome segregation. In contrast, the mechanism of MC segregation seems entirely dissimilar, as it is based on the MamK actin-like cytomotive protein resembling the type-II partitioning system, which is also defined by dynamic actin-like filaments represented by the ParM family [59]. Thus, magnetosome segregation mechanisms could classify as a type-II-like system, where MamJ could serve as an adaptor between the motor protein (MamK) and cargo (magnetosomes). Our model is also reminiscent of the myosin-actin organelle transport in yeast [60] in which most organelles are segregated by the actin cytoskeleton operating with the motor myosin V protein [60, 61]. Although this comparison currently seems speculative, we hypothesize that the non-motor cargo-associated MamJ does not “walk” onto the MamK filament as myosin does on actin, but instead MamJ is carried by MamK, like “a luggage on a belt”, continuously “hopping on and off” along the MamK filament.

## Conclusion

Our findings provide direct evidence to sustain that, after cell division, MCs are in fact repositioned from the newborn cell pole to the cell center of the daughter cell as hypothesized in previous work. In addition, our data support the concept that MC motion is directly dependent on the dynamics of the MamK filaments suggested to undergo treadmilling. Thus, MamK filaments display a positive growing end located at the cell poles, where MamK units are incorporated to the filament, thereby pushing the units already in the filament towards the cell center. Finally, we demonstrated that the dynamics of MamK filaments is a fundamental feature for proper MC equipartitioning, intracellular motion and, ultimately, faithful organelle segregation. In conclusion, we have dissected here, for the first time, the mechanism of segregation of a cytoskeleton-interacting bacterial organelle, which seems to be independent of endogenous segregation systems and instead utilizes a dedicated mechanism controlled by magnetosome-specific proteins.

## Methods

### Bacterial strains, plasmids and culture conditions

Bacterial strains and plasmids generated and used in this work are listed in Table A and Table B within Additional file 22: Supporting Methods. The plasmid construction procedure is described in Additional file 22: Supporting Methods. Strains of *M. gryphiswaldense* MSR-1 were grown under microoxic conditions in 2 % oxygen aerated modified flask standard medium [62] (FSM) containing 50 μM ferric citrate and low iron media [63] (LIM, modified FSM) at 30 °C and moderate agitation (120 r.p.m.). *E. coli* strains DH5α and BW29427 (Datsenko and Wanner, Purdue University, IN, USA) were grown in LB medium at 37 °C [64]. *E. coli* strain BW29427 used for conjugations of plasmids into MSR was supplemented with 1 mM DL-α,ε-diaminopimelic acid (DAP). For strains carrying recombinant plasmids, media were supplemented with kanamycin at 25 g mL^–1^ for *E. coli* and 5 g mL^–1^ for MSR.

### In vivo time-lapse fluorescence microscopy

For time-lapse imaging, two approaches were used. First, an Olympus BX81 microscope equipped with a 100 X UPLSAPO100XO objective with a numerical aperture of 1.40 and an Orca-ER camera (Hamamatsu) was used. For time-lapse microscopy, synchronized cells (as described by Katzmann et al. [19]) were fixed on agarose pads in a 1:1 ratio of culture:agarose (LMP) 1.5 %. The coverslips with the immobilized cells were then placed into a Ludin chamber (Life Imaging Services, Basel, Switzerland). Cells were imaged at room temperature (25 °C) every 5 or 10 min for up to 10 h and focused manually (in DIC channel) before frame acquisition. Phototoxicity was kept at minimum by using 20 % of the light source power and an exposure of 200 ms.

Alternatively, cells were spotted onto a 1 % agarose pad with a special FSM medium preparation (8 mM nitrate, 1 g L^–1^ peptone, without iron or trace elements, from now on referred to as microscopy MSR agarose pad) and sealed with paraffin wax. In this case, cells were imaged with a Delta Vision Elite (GE Healthcare, Applied Precision) with a CoolSnap HQ2 CCD camera (Photometrics) and using an Olympus IX71 microscope equipped with a four color standard set Insight SSI^TM^ illumination module with the following excitation wavelengths: DAPI 390/18 nm, FITC 475/28 nm, TRITC 542/27 nm, Cy5 632/22 nm; single band pass emission wavelengths DAPI 435/48 nm, FITC 525/48 nm, TRITC 597/45 nm, Cy5 679/34 nm and a suitable polychroic beam splitter. Time-lapse imaging was performed at 30 °C with a hardware based “Ultimate-Focus” autofocus and images were collected with a 100× Oil PSF Objective (U-PLAN S-APO 100X Oil, 1.4NA, 0.12 WD) using the FITC filter set for imaging of MamC-EGFP.

### Scoring of magnetosome chain partitioning

To quantify the uniformity of the MC partitioning, cells were grown under microoxic conditions and then transferred for 5 h to 21 % oxygen. Subsequently, cells were fixed (1 % formaldehyde) and TEM grids were prepared. Only cells undergoing division were taken into account, chain partitioning was scored in terms of length and magnetosome number fractions inherited per each future daughter cell. The total length or crystal numbers of the chain (considering the sum of both chains from the future cells) correspond to 100 %. A linear and exponential function were used to fit to the cumulative distribution of the fraction of the chain in one daughter cell:

$$ F(x)=1- \exp \left(-\frac{x-50\%}{\lambda}\right) $$

This cumulative distribution corresponds to a distribution of the chain fraction that decays exponentially from 50 %, for *x* between 50 and 100 %. The parameter λ represents the accuracy with which segregation happens in the center of the MC. In addition, the half-maximum of the cumulative distribution (F(*x*) = 0.5) corresponds to the median of the distribution and characterizes the deviation from equal partitioning (50:50).

### Scoring of magnetosome positioning

To assign MC position in several strains, cells were grown microoxically, fixed, and absorbed onto TEM grids (see below). Intracellular MC positions were scored based on TEM micrographs. To this end, MSR cells were divided into three main sections: (1) Pole, within 400 nm from the cell pole or a constriction site; (2) Adjacent: approximately 400–800 nm away from nearest cell pole or constriction; and (3) Midcell: between adjacent areas, i.e., more than 800 nm away from the nearest constriction (Additional file 5: Figure S2A). To assign a chain to a section, the chain outermost particle/magnetosome closest to the pole was considered. This particle defined the position of the chain inside the cell. Special consideration to differentiate between Adjacent and Midcell chains was that at least 51 % of the chain must fall into the Adjacent area to be ascribed as such.

### Simulations of magnetosome chain dynamics

Formation of the chain and relocation to midcell after cell division was simulated with the model of Klumpp and Faivre [34]. The friction coefficient γ was chosen independent of the diffusion coefficient *D*, which allows to interpret active transport as having a linear force velocity in relation with stall force F_s_ and zero-force velocity v_0_ = F_s_/γ. For simulations of movements after division, we started simulations with 15 magnetosomes of maximal size and magnetization, regularly spaced at the boundary of the simulation volume representing the cell.

### Induction of *de novo* magnetite crystal formation

Iron induction experiments were carried out as per Scheffel et al. [25]. For details see Additional file 22: Supporting Methods. Briefly, cells were grown in 6-well plates with LIM medium in a microaerobic environment. For induction of magnetite biomineralization 100 μM Fe(III)-citrate was supplemented to cells, which were previously iron-starved by four passages in LIM medium [25, 63].

### Photokinetic analysis

Photokinetic experiments, such as fluorescence recovery after photobleaching (FRAP) and photoconversion, were performed on *M. gryphiswaldense* strains expressing several constructs. For genes under control of the tetracycline promoter, the induction of expression was initiated by addition of anhydrotetracycline to 50 ng mL^–1^. Cells were mounted on microscopy MSR agarose pads. Cells were imaged with a Delta Vision Elite system (GE Healthcare, see above) at 30 ºC. Time-lapse imaging was also performed at 30 °C with a hardware based “Ultimate-Focus” autofocus and images were collected with a 100× Oil PSF Objective using specific settings for each protein fusion as described in Additional file 22: Supporting Methods.

#### Dark-state fluorophore reversal control

Dark-state reversal of each fluorophore fusion was controlled by fixing cells with 1 % formaldehyde for 1 h at room temperature prior to a laser pulse and image analysis.

#### Image and data analysis

Images were aligned (StackReg plugin [65]) and further analyzed with Fiji [66], each region of interest was background subtracted and corrected for bleaching by considering the whole cell fluorescence as described by Gavet and Pines [67], and Kiekebush et al. [68]. Relative values were used in order to allow comparison and further averaging of several cells. A value of 1 corresponds to the quotient of the integrated density corrected by whole cell fluorescence, before the bleaching event per selected regions (bleached and unbleached areas, and whole filament when required). Thus, we did not quantify the recovery of the bleached part of the filamentous structure analyzed here, but we have followed the fluorescence recovery in the zone of the cell were the laser was initially applied. The average values of 20 to 41 MSR cells of several biological replicates were plotted. Recovery rates were determined by fitting the data obtained for the bleached region to the single exponential function:

$$ F(t)=A\ \left[1- \exp \left(-k*t\right)\right]+F(0) $$

Where F(*t*) is the fluorescence at time *t*, A the maximum intensity, *k* the rate constant and F(0) the relative fluorescence intensity at *t* = 0 min.

Quantification of the photoconversion data was obtained as described above for FRAP data. Briefly, after correction by whole cell fluorescence, relative values were used in order to allow comparison and further averaging of several cells. A value of 1 was assigned, however, to integrated density of the photoconverted area at *t* = 0 min (measured immediately after the laser event), as it is the maximum fluorescent signal possibly obtained. Signal decay or increment rates were determined by fitting the data obtained to the photoconverted pole, non-polar and non-photoconverted pole areas using the single exponential function shown above.

### MamK filament treadmilling speed measurement

Fiji software was employed to quantify the MamK filament treadmilling growth speed from the mCherry-MamK FRAP data (imaging performed every 30 s). To this end, only cells bleached at or near the polar area were considered, as the filament growth front line is more conspicuous to visualize as well as giving greater time space to follow it. Thus, treadmilling growth was determined manually by meticulously following the filament recovery front line in each case. The scalar magnitude distance of the advancement of the filament growth front line was determined as a function of the time generating speed in nm/min. Filament treadmilling speed was calculated by assessing a minimum of four consecutive frames, equivalent to a 90 s interval. In order to avoid data misinterpretation, the quantification began only when the filament growing front line was clearly distinguishable, usually 60 s post-bleaching at the cell pole. This period was necessary to discard miscalculations in speed by waiting for the pole to first recover the fluorescence. The average values of 20 to 36 bleached filaments for each strain from several biological replicates were plotted. Graphs were plotted and statistical tests were run with GraphPad Prism 5.0.

### Transmission electron microscopy (TEM)

For conventional TEM analysis, cells were grown at 28 °C under microaerobic conditions, fixed in formaldehyde (1 %) and concentrated. Next, unstained cells were absorbed on carbon coated copper mesh grids (Plano, Wetzlar). Bright field TEM was performed on a FEI CM200 (FEI; Eindhoven, The Netherlands) transmission electron microscope using an accelerating voltage of 160 kV. Images were captured with an Eagle 4 k CCD camera using EMMenu 4.0 (Tietz) and FEI software. For data analysis the Fiji software was used.

### Cryo-electron tomography (CET)

For CET, 5 μL of MSR culture mixed with 5 μL of 15 nm colloidal gold clusters (Sigma, for subsequent alignment purposes) were added on glow-discharged Quantifoil holey carbon copper grids (Quantifoil Micro Tools GmbH, Jena), blotted and embedded in vitreous ice by plunge freezing into liquid ethane (< −170 °C). Samples were assessed with a FEI Tecnai F30 Polara transmission electron microscope (FEI; Eindhoven, the Netherlands), equipped with 300 kV field emission gun, a Gatan GIF 2002 Post-Column Energy Filters, and a 2 K Multiscan CCD Camera (Gatan; Pleasanton, CA). Data collection was performed at 300 kV, with the energy filter operated in the zero-loss mode (slit width of 20 eV). Tilt series were acquired using Serial EM [69] and FEI software. The specimen was tilted about one axis with 1.5° increments over a typical total angular range of ± 65°. To minimize the electron dose applied to the ice-embedded specimen, data were recorded under low-dose conditions. The total dose accumulated during the tilt series was kept below 150 e Å^–2^. To account for the increased specimen thickness at high tilt angles, the exposure time was multiplied by a factor of 1/cos α. The object pixel size in unbinned images was 0.71 at a magnification of 18,000×. Images were recorded at nominal −7 μm defocus.

### CET data analysis

Three-dimensional reconstructions from tilt series were performed with the weighted back-projection method using the TOM toolbox [70]. Membrane segmentation was done using the software TomoSegMemTV and a complementary package, SynapSegTools, both for Matlab [71]. Visualizations of the tomograms were done with Amira software "(FEI company)"on one-time binned volumes.

## Additional files

Additional file 1: Movie S1. In vivo time-lapse imaging of magnetosome chain motion in wildtype strain. Fluorescence microscopy video of a 95 min study with frames every 5 min as shown in Fig. 1a. Movie was performed at 4 fps. Scale bar: 1 μm.
Additional file 2: Figure S1. Alignment of actins ATPase motifs and magnetosome chain parameters: mean-squared displacement, apparent diffusion constant and velocity. (**A**) The Connect 1 and Phosphate 2 motif from the ATPase domain [72] were aligned from several prokaryotic actins and the human actin. Red residues indicate high conservation. The residues of the MSR MamK protein, glutamate E143 and aspartate D161, mutated in this work are indicated. Alignment was performed in CLC Main Workbench software as per Derman et al. [9] using the following sequences: MSR MamK (CAM78025.1), AMB MamK (WP_011383398.1), *E. coli* ParM (WP_000959884.1), *Bacillus subtillis* MreB (WP_003229650.1), *B. subtillis* AlfA (WP_013603336.1), *B. subtillis* Alp7A (WP_013603291.1), *Homo sapiens* Actin (NP_001605.1). (**B**) Magnetosome chain (MC) mean-squared displacement (MSD) as a function of time in the wildtype (WT) (*n* = 24) and *mamK D161A* (*n* = 19) strains. MSD was determined from the MamC-EGFP fluorescence signal. (**C**) MC apparent diffusion constant (D*) as a function of time calculated from MSD data. (**D**) MC velocity (V_MC_) determined from displacement data (time interval: 10 min) for the WT and *mamK D161A* strains. (PDF 141 kb)
Additional file 3: Movie S2. In vivo time-lapse imaging of magnetosome chain motion in *mamK D161A* strain. Fluorescence microscopy video of a 240 min study with frames every 10 min as shown in Fig. 1c. Movie was performed at 4 fps. Scale bar: 1 μm.
Additional file 4: Movie S3. Overview of in vivo time-lapse imaging of magnetosome chain motion in *mamK D161A* strain. Fluorescence microscopy imaging during 13 h with frames every 10 min. Movie was performed at 7 fps. Scale bar: 1 μm.
Additional file 5: Figure S2. Intracellular magnetosome chain position. (**A**) Scheme representing how the intracellular positioning assessment was carried out. Several TEM micrographs were analyzed and the newly formed end of each chain was used to determine its position. For instance, when a chain end was found within a determined area, the chain was assigned to that position. (**B**) TEM micrograph of a *mamK D161A* cell displaying a polar localized magnetosome chain (MC). Scale bar: 1 μm. (**C**) Bar plot representing data of cells with a single or multiple MCs. (**D**) Bar plot representing the same data set as in “B”, but cells having single or multiple MCs are plotted independently. *Magnetospirillum gryphiswaldense* MSR-1 wildtype (*n* = 312), *mamK D161A* (*n* = 929: single = 373, multiple = 556) and ∆*mamK* (*n* = 514: single = 282, multiple = 232). (PDF 847 kb)
Additional file 6: Figure S3. Magnetosome chain (MC) segregation. (**A**) Quantification of the MC segregation in terms of magnetosomes per cell to be inherited by future daughter cells (wildtype (WT): *n* = 83; *mamK D161A*: *n* = 68). Cells were incubated under 21 % oxygen for 5 h. (**B**) Cumulative distribution of MC segregation data in “A”. (**C**) Quantification of the MC segregation length to be inherited by future daughter cells (WT: *n* = 39; *mamK D161A*: *n* = 123). Cells were grown under microoxic conditions (2 % oxygen). (PDF 200 kb)
Additional file 7: Figure S4. Simulations of magnetosome chain (MC) dynamics. (**A**) MC motion after cell division was modeled under low and high diffusive mobility varying (**B**) velocity (V_0_) or (**C**) force (F_s_). (PDF 261 kb)
Additional file 8: Figure S5. Magnetite crystal size and *mamK D161A* strain phenotypes. (**A**) Quantification of magnetosome chains (MCs) per cell under microoxic growth (2 % oxygen) in *Magnetospirillum gryphiswaldense* MSR-1 wildtype (*n* = 298), *mamK D161A* (*n* = 521) and ∆*mamK* (*n* = 469). (**B**) TEM micrographs of *mamK D161A* displaying wildtype-like (left panel) or fragmented (right panel) MCs. Scale bars: 1 μm. (PDF 1828 kb)
Additional file 9: Figure S6. Connected cells of *mamK D161A* strain. TEM micrographs of *mamK D161A* displaying four cells that seem completely divided, albeit still connected by some membranous bridging structures (indicated by *arrows*) and suggested to be held by MamK^D161A^ stable filaments. Scale bars: 1 μm. (PDF 1014 kb)
Additional file 10: Figure S7. Fluorescence microscopy of mCherry-MamK^D161A^ filament in connected cells. Cell membrane was stained with the fluorescent dye CellBrite^TM^ Blue. *Arrows* indicate the cell division site constriction where the mCherry-MamK^D161A^ filament signal appears to cross. Scale bar: 1 μm. (PDF 16 kb)
Additional file 11: Figure S8. Cryo-electron tomography (CET) of cell division sites of *mamK D161A* connected cells. (**A**) CET micrograph of three connected cells. The indicated cell division site (placed on the grid’s hole) was selected for tomography (imaged cell division site in Fig. 4b–d). (**B**) CET micrograph of three connected cells. Both cell division sites (*arrowheads*) were suitable for tomography. (**Ci-Civ**) CET sections of cell division site 1, (**Di-Dv**) and cell division site 2 as indicated in “B”. *White* *arrowheads* indicate MamK filaments entering and exiting the membranous bridge. *Blue arrows* indicate inner (IM) and outer (OM) membranes. (**Ei-Eiv**) CET 3D rendering of the cell division site 1, (**Fi-Fiii**) and cell division site 2 indicated in “B”. Magnetite crystals: *red*. Vesicles: *yellow*. MamK filament: *green*. Cellular envelope, inner and outer membrane: *blue*. Flagella: *gold*. (PDF 4458 kb)
Additional file 12: Movie S4. Cryo-electron tomography of *mamK D161A* connected cells. View through the z-stack tomogram of the division site of *mamK D161A* connected cells at 18,000× magnification from Fig. 4b–d. Vesicles (*yellow*) and magnetite (*red*) associated with the MamK filament structure (*green*), which traverses the cell division site. Cellular envelope (*blue*).
Additional file 13: Movie S5. Cryo-electron tomography of *mamK D161A* connected cells (Cell 2, division site 1). View through the z-stack tomogram of a cell division site of *mamK D161A* connected cells at 18,000× magnification from Additional file 11: Figure S8B, S8C and S8E. Vesicles (*yellow*) and magnetite (*red*) associated with the MamK filament structure (*green*), which traverses the cell division site. Cellular envelope (*blue*).
Additional file 14: Movie S6. Cryo-electron tomography of *mamK D161A* connected cells (Cell 2, division site 2). View through the z-stack tomogram of a cell division site of *mamK D161A* connected cells at 18,000× magnification from Additional file 11: Figure S8B, S8D and S8F. Vesicles (*yellow*) and magnetite (*red*) associated with the MamK filament structure (*green*), which traverses the cell division site. Cellular envelope (*blue*).
Additional file 15: Figure S9. Controls and curves of fluorescence recovery after photobleaching. Dark-state fluorophore reversal was controlled by fixing the cells with 1 % formaldehyde for 1 h and then FRAP was performed. Therefore, the translational fusion mCherry-MamK expressed from (**A**) the *mamK* locus or (**B**) a replicative plasmid in ∆*mamJK* background was evaluated in fixed cells. (**C–F**) Comparison of fluorescence recovery curves over the time after the laser application for different strains. Only bleached areas were plotted for each strain. Zero time was measured immediately after laser pulse. SD corresponding to each time point is not showed for a better comparison of the curves. (PDF 455 kb)
Additional file 16: Figure S10. Half-time fluorescence recovery of MamK. (**A**) Half-time recovery of the fluorescence (t_½_) of mCherry-MamK in several strains is presented as bar chart highlighting significant differences. An unpaired Student’s *t*-test was carried out. * Significant: *P* = 0.01 to 0.05. ** Very significant *P* = 0.001 to 0.01. *** Extremely significant *P* < 0.001. ns: not significant. (**B**) Half-time recovery of the fluorescence (t_½_) box and whiskers plot for a better comparison of data distribution among strains. Error bars in “A” represent SEM and in “B” the 10–90 percentile. Lines inside the box specify the median. (**C**) Detailed half-time recovery of the fluorescence (t_½_) with SEM per strain. (PDF 273 kb)
Additional file 17: Movie S7. MamK filament treadmilling. Fluorescence microscopy imaging of mCherry-MamK after photobleaching of the filament by a 561 nm laser pulse applied between the first and second frame. Imaging was carried out for 10 min with frames every 30 s. Upper panel shows *mCherry-mamK* expressed in the wildtype (chromosomally, Fig. 5a), Δ*mamK* and MSR-1B, respectively. Lower panel (left to right): *mCherry-mamK* expressed in MSR Δ*mamJK* and *E. coli* (as shown in Additional file 18: Figure S11B). Movie was performed at 7 fps. Scale bar: 1 μm.
Additional file 18: Figure S11. MamK filament treadmilling and dynamics. Bleach-marked mCherry-MamK filaments were used to follow the treadmilling in (**Ai**) MSR (chromosomal expression from the *mamK* locus. Fig. 5a) and (**Bi**) *E. coli* (expression from the *lac* promoter induced for 9 h, 1 mM IPTG). (**Aii and Bii**) Kymographs displaying fluorescence signal intensity (*x-*axis) of bleached MamK filaments over the time (*y-*axis) in the respective strains. The corresponding duplicated kymograph indicates the bleaching time/area (*red box*) and filament fluorescent signal progression (*white dashed line*). The bleach-marked filaments were followed for 5 min with imaging every 30 s. (**C**) Two kymographs of the *red* photoconverted Dendra2-MamK signal (*x-*axis) over time (*y-*axis). The corresponding duplicated kymograph below indicates the photoconversion time/area (*red box*) and MamK filament fluorescent signal appearance/progression from the cell pole (*red dashed line*). Right panel: cell shown in Fig. 7bi. (**D**) Quantification of photoconverted Dendra2-MamK^D161A^ signal in the WT strain (*n* = 16). (**E**) Photoconversion of Dendra2-MamK (*n* = 31) or (**F**) Dendra2-MamK E143A (*n* = 39) expressed from the tetracycline-inducible promoter (P_*tet*_), 6 h induced. *Green channel*: filament prior to photoconversion. *Red channel*: photoconverted protein after a 405 nm laser pulse. *White dashed circles:* photoconverted areas. *Arrowhead* indicates appearance of MamK signal at the cell pole. *White dashed lines* act as reference point. *Red dashed lines: *filament growth progression. (**G**) Dot plot of MamK filament treadmilling speed distribution in several strains, quantified from mCherry-MamK photobleaching data. Scale bars: 1 μm. (**H**) Detail of MamK filament treadmilling speeds. (**I**) Correlation plot of MamK filament growth speed versus the corresponding half-time fluorescence recovery. A linear regression (*r* ^2^ = 0.8275) shows negative correlation between speed and fluorescence recovery. Pearson correlation coefficient (*r*) shows a significant (*P* = 0.01 to 0.05) negative linear relationship (*r* = –0.9097). *E. coli* data was excluded of this analysis. (PDF 521 kb)
Additional file 19: Figure S12. MamJ dynamics. (**A**) Photobleaching of MamJ-mCherry co-expressing *mamK* and evaluation of the fluorescence recovery in ∆*mamJK*. The right panel shows the quantification of the MamJ-mCherry fluorescence recovery over the time. Zero time was measured immediately after laser pulse. Half-time recovery of the fluorescence is presented as t_½_ in the plot. (**B**) Photoconversion of Dendra2 fused to MamJ in MSR wildtype. *Arrow *indicates MamJ signal progression. Similar behavior was observed in other analyzed cells (*n* = 22). (**C**) Photoconversion of MamJ-Dendra2 (*n* = 10) co-expressed with *mamK* in ∆*mamJK*. *Arrow* indicates appearance and progression of MamJ signal at the cell pole. (**D**) Photobleaching of MamJ-mCherry co-expressing *mamK D161A* under the control of the tetracycline-inducible promoter (P_*tet*_) (24 h induced) and evaluation of the fluorescence recovery in MSR ∆*mamK* and (**E**) ∆*mamJK* strains. The plots show quantification of the MamJ-mCherry fluorescence recovery over the time. Zero time was measured immediately after laser pulse. Half-time recovery of the fluorescence is presented as t_½_ in the plot. (**F**) Expression of *mamJ-dendra2* co-expressing *mamK D161A* (*n* = 15) from the tetracycline-inducible promoter (P_*tet*_) (24 h induced) and posterior photoconversion in MSR ∆*mamJK* strain. *Green channel* displays the filament prior to photoconversion. *Red channel* shows photoconverted protein after a laser line 405 nm pulse application. (PDF 573 kb)
Additional file 20: Figure S13. Controls of MamJ dynamics assays. (**A**) Representative cell of a fixed control of MamJ-Dendra2 photoconversion co-expressing *mamK* (*n* = 4) in MSR wildtype (WT) strain. *Green channel* displays the filament prior to photoconversion. *Red channel* shows photoconverted protein after a laser line 405 nm pulse application. (**B**) Control for dark-state-reversal of mCherry fluorophore fused to MamJ and co-expressing *mamK* in MSR WT (*n* = 15) and (**C**) ∆*mamJK* strains (*n* = 15). (**D**) Control for dark-state-reversal of MamJ-EGFP fusion expressed under the control of the *mamGFDC* operon (located in the MAI) promoter (P_*mamDC*_) from a Tn5-based chromosomal insertion in the MSR WT strain (*n* = 12). Zero time was measured immediately after laser pulse. Scale bars: 1 μm. (PDF 322 kb)
Additional file 21: Figure S14. MamK and MamJ functional complementation. (**A**) ∆*mamJK* cells forming clustered magnetosomes were used for evaluation of functionality of the constructs. (**B**) ∆*mamJK* strain co-expressing *mamK* and *mamJ*-*mCherry* from a replicative plasmid. (**C**) ∆*mamJK* strain expressing *mamJ*-*dendra2* from a replicative plasmid. (**D**) ∆*mamJK* strain co-expressing *mamK* and *mamJ*-*dendra2* from a replicative plasmid. (**E**) ∆*mamJK* strain co-expressing *mamK D161A* together with either *mamJ*-*mCherry* or (**F**) *mamJ*-*dendra2* from a replicative plasmid under the control of the tetracycline-inducible promoter (P_*tet*_) (24 h induced). Reconstitution of linear magnetosome chains (MCs) indicated successful gene expression and complementation of the phenotype. Notably, in (E) and (F), the *mamK D161A* phenotype of MC mispartitioning, polar localization and segmented chains were also observed in the complemented strains devoid of the *mamK* gene. Scale bars: 1 μm. (PDF 3422 kb)
Additional file 22: Supporting Methods. Text including details of methods that were not included in the main text (Additional file 23: Figure S15). (DOCX 190 kb)
Additional file 23: Figure S15. MamK immunoblot. MamK protein presence was evaluated by western blot in the MSR WT, *mamK D161A mamC-egfp*, *mCherry-mamK D161A* and ∆*mamK* strains. MamK: ~37 KDa (*lower arrowhead*). mCherry-MamK: ~68 kDa (*upper arrowhead*). (PDF 50 kb)

## Abbreviations

AMB: *Magnetospirillum magneticum* AMB-1
CET: cryo-electron tomography
C_mag_: magnetic response
MC(s): magnetosome chain(s)
MSR: *Magnetospirillum gryphiswaldense* MSR-1
TEM: Transmission electron microscopy
