# Supplementary material for: Segregation of prokaryotic magnetosomes organelles is driven by treadmilling of a dynamic actin-like MamK filament
Source: BMC Biol. 2016 Oct 12;14:88. doi: 10.1186/s12915-016-0290-1 (PMC5059902; doi:10.1186/s12915-016-0290-1)
Supplement: Additional file 2: Figure S1. — Alignment of actins ATPase motifs and magnetosome chain parameters: mean-squared displacement, apparent diffusion constant and velocity. (A) The Connect 1 and Phosphate 2 motif from the ATPase domain [72] were aligned from several prokaryotic actins and the human actin. Red residues indicate high conservation. The residues of the MSR MamK protein, glutamate E143 and aspartate D161, mutated in this work are indicated. Alignment was performed in CLC Main Workbench software as per Derman et al. [9] using the following sequences: MSR MamK (CAM78025.1), AMB MamK (WP_011383398.1), E. coli ParM (WP_000959884.1), Bacillus subtillis MreB (WP_003229650.1), B. subtillis AlfA (WP_013603336.1), B. subtillis Alp7A (WP_013603291.1), Homo sapiens Actin (NP_001605.1). (B) Magnetosome chain (MC) mean-squared displacement (MSD) as a function of time in the wildtype (WT) (n = 24) and mamK D161A (n = 19) strains. MSD was determined from the MamC-EGFP fluorescence signal. (C) MC apparent diffusion constant (D*) as a function of time calculated from MSD data. (D) MC velocity (VMC) determined from displacement data (time interval: 10 min) for the WT and mamK D161A strains. (PDF 141 kb) [file 12915_2016_290_MOESM2_ESM.pdf]

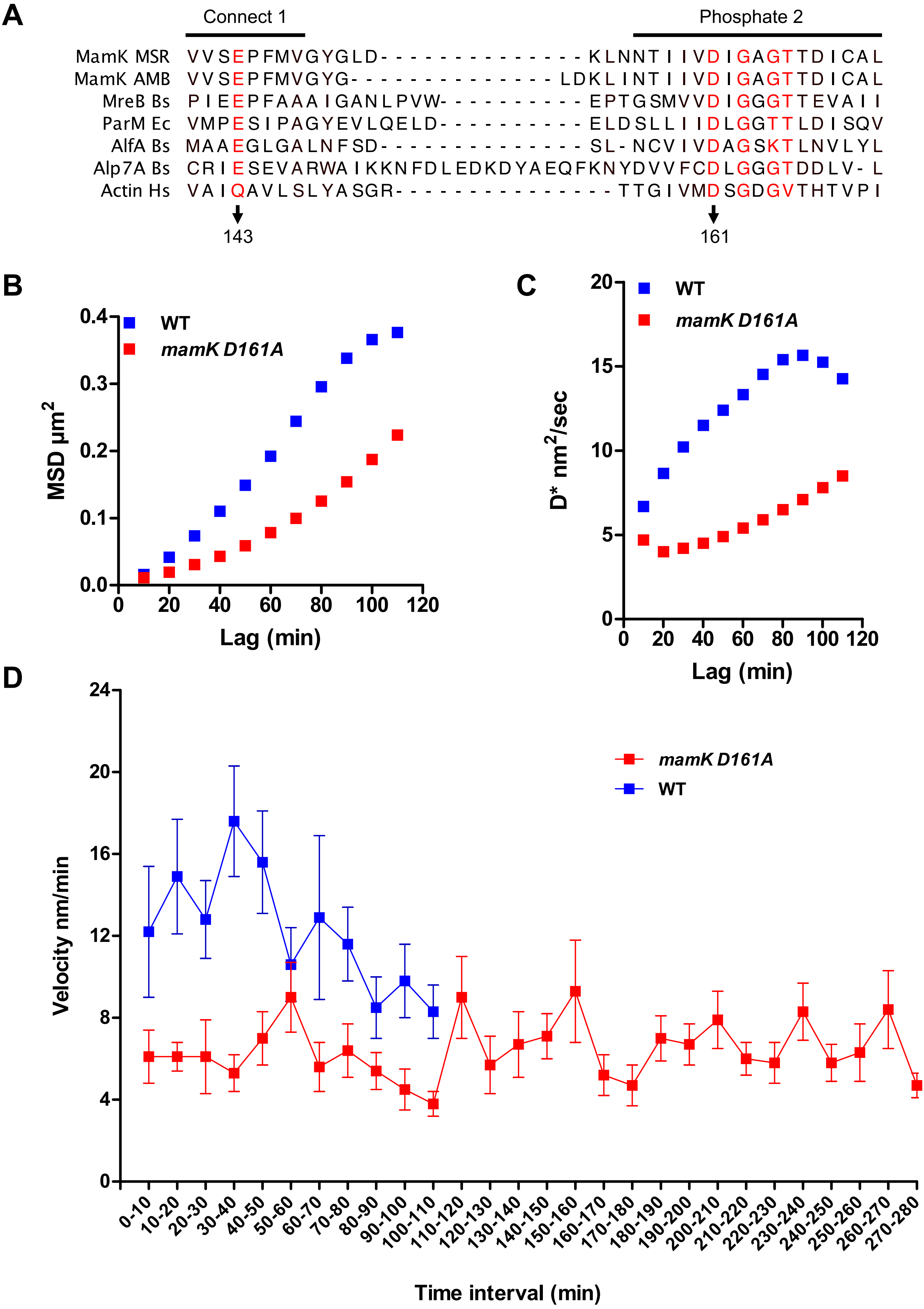

A
Connect 1
Phosphate 2
MamK MSR VVSEPFMVGYGLD---------------KLNNTIIVDIGAGTTDICAL
MamK AMB VVSEPFMVGYG---------------LDKLINTIIVDIGAGTTDICAL
MreB Bs PIEEPFAAAIGANLPVW-----------EPTGSMVVDIGGGTTEVAII
ParM Ec VMPESIPAGYEVLQELD-----------ELDSLLIIDLGGTTLDISQV
AlfA Bs MAAEGLGALNFSD---------------SL-NCVIVDAGSKTLNVLYL
Alp7A Bs CRIESEVARWAIKKNFDLEDKDYAEQFKNYDVVFCDLGGGTDDLV-L
Actin Hs VAIQAVLSLYASGR----------------TTGIVMDSGDGVTHTVPI
143
161
B
WT
mamK D161A
MSD µm²
0.0
0.1
0.2
0.3
0.4
0
20
40
60
80
100
120
Lag (min)
C
WT
mamK D161A
D* nm²/sec
0
5
10
15
20
0
20
40
60
80
100
120
Lag (min)
D
mamK D161A
WT
Velocity nm/min
0
4
8
12
16
20
24
0-10
10-20
20-30
30-40
40-50
50-60
60-70
70-80
80-90
90-100
100-110
110-120
120-130
130-140
140-150
150-160
160-170
170-180
180-190
190-200
200-210
210-220
220-230
230-240
240-250
250-260
260-270
270-280
Time interval (min)
