# Supplementary figures and images for: Segregation of prokaryotic magnetosomes organelles is driven by treadmilling of a dynamic actin-like MamK filament
Source: BMC Biol. 2016 Oct 12;14:88. doi: 10.1186/s12915-016-0290-1 (PMC5059902; doi:10.1186/s12915-016-0290-1)

**A**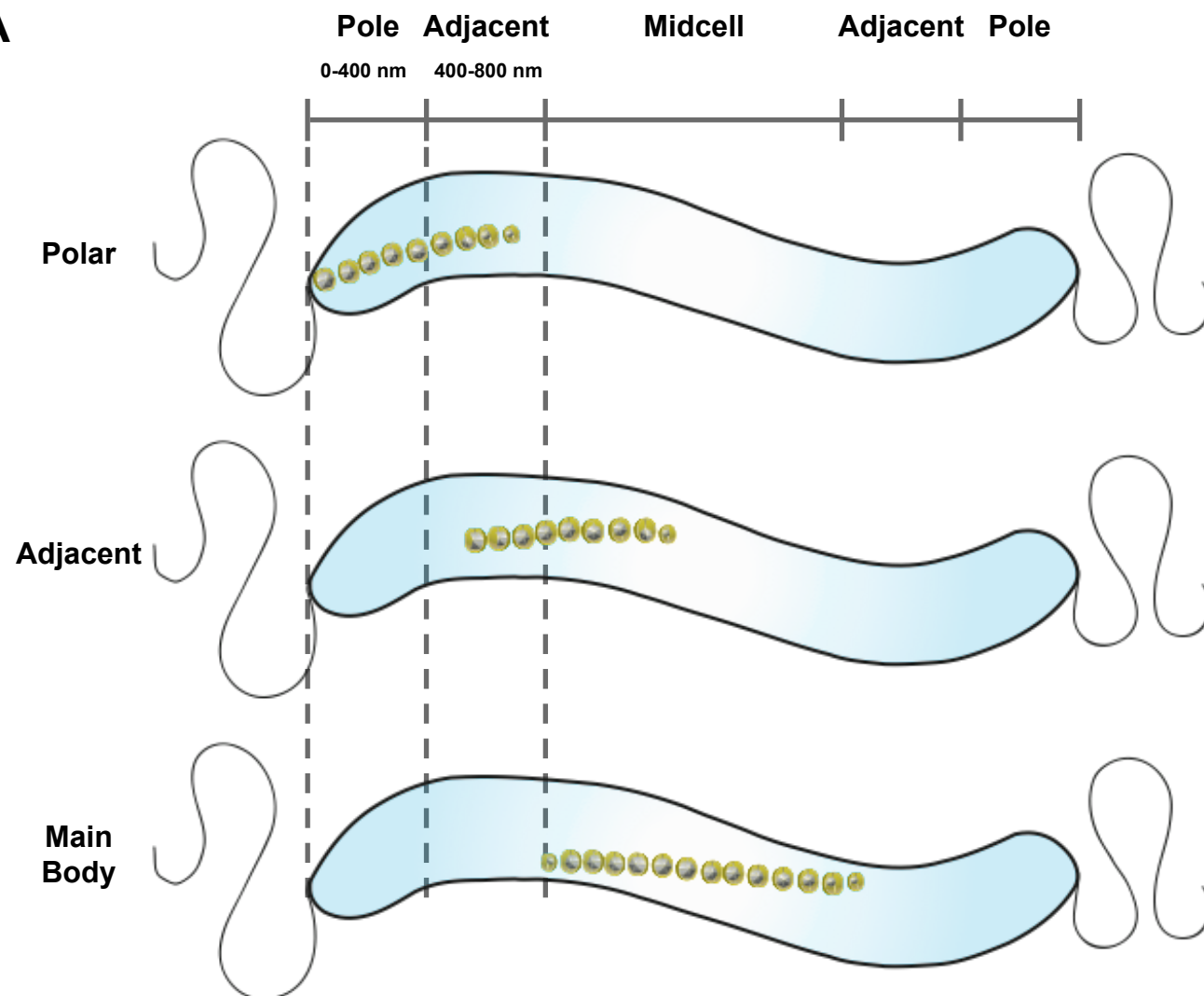**B**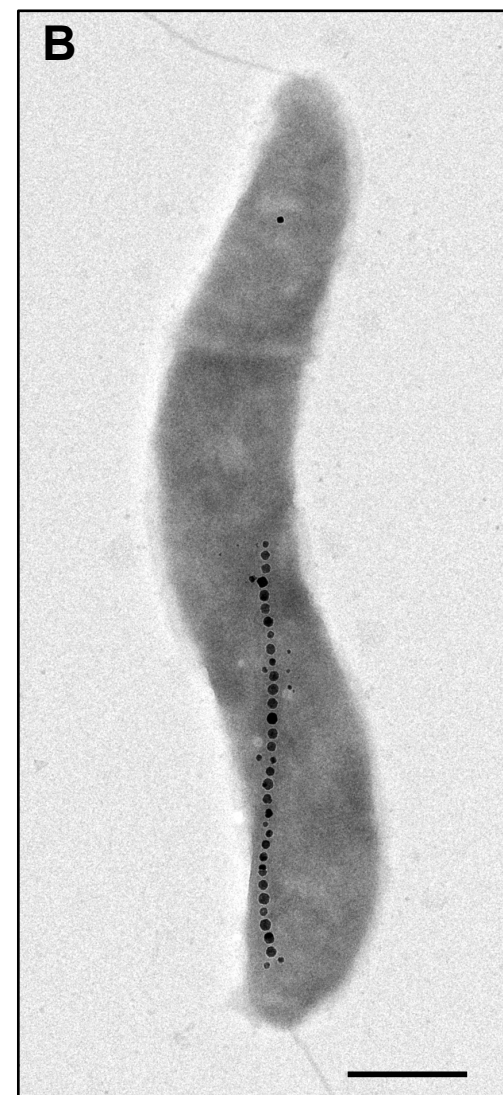**C**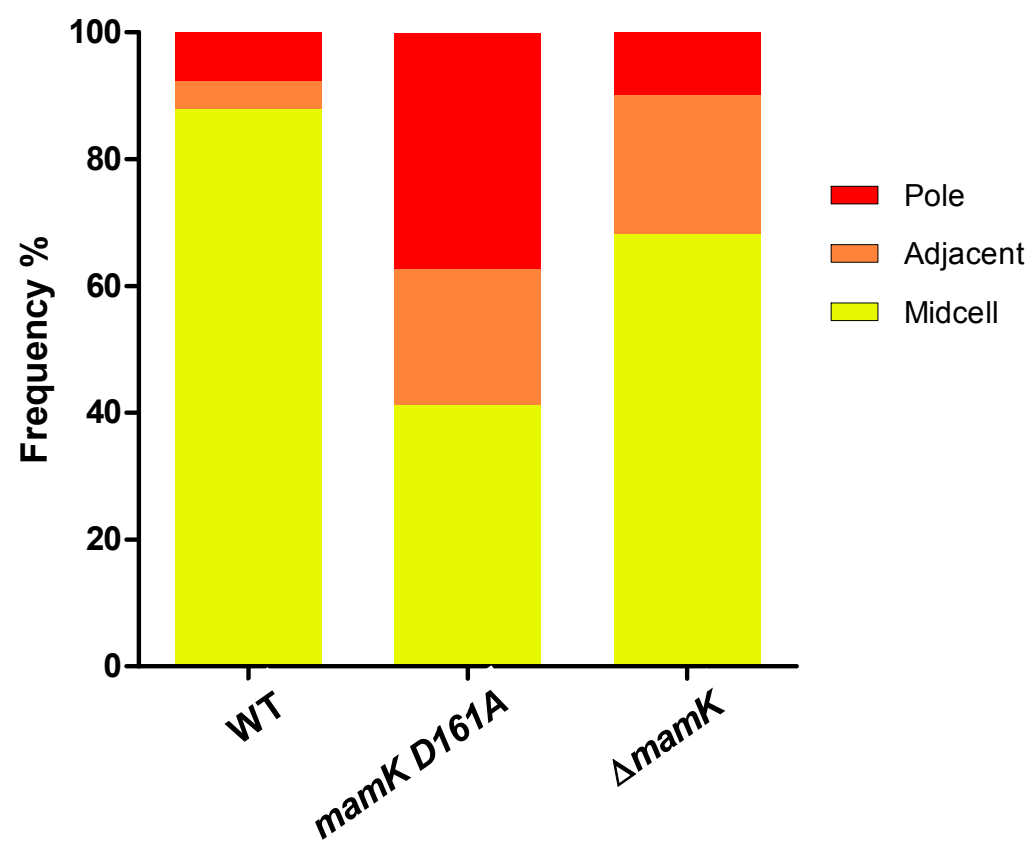**D**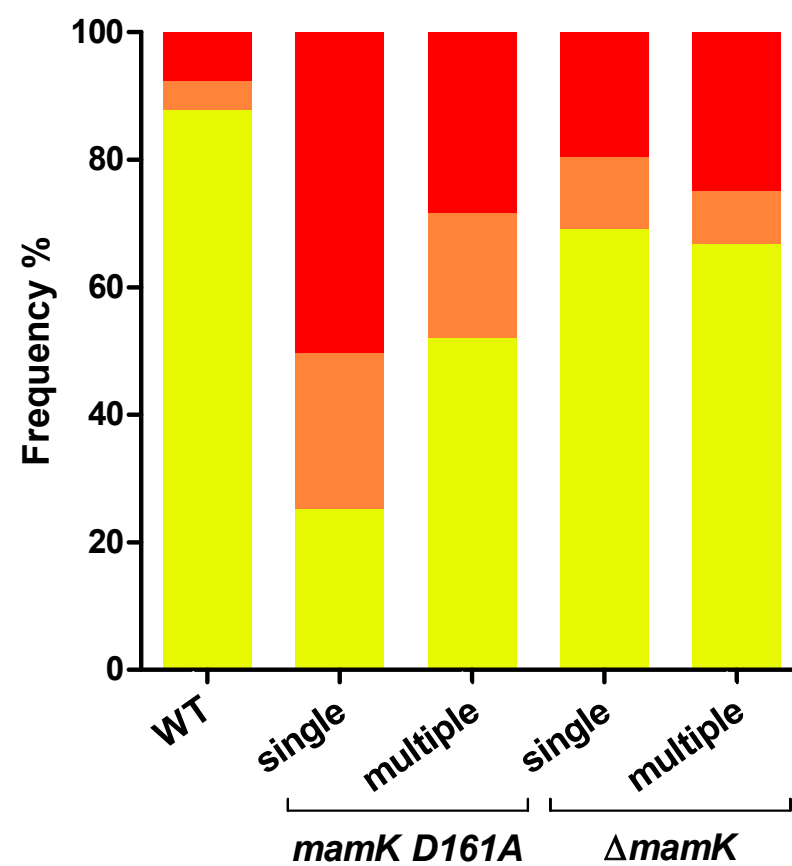

Supplement: Additional file 5: Figure S2. — Intracellular magnetosome chain position. (A) Scheme representing how the intracellular positioning assessment was carried out. Several TEM micrographs were analyzed and the newly formed end of each chain was used to determine its position. For instance, when a chain end was found within a determined area, the chain was assigned to that position. (B) TEM micrograph of a mamK D161A cell displaying a polar localized magnetosome chain (MC). Scale bar: 1 μm. (C) Bar plot representing data of cells with a single or multiple MCs. (D) Bar plot representing the same data set as in “B”, but cells having single or multiple MCs are plotted independently. Magnetospirillum gryphiswaldense MSR-1 wildtype (n = 312), mamK D161A (n = 929: single = 373, multiple = 556) and ∆mamK (n = 514: single = 282, multiple = 232). (PDF 847 kb) [file 12915_2016_290_MOESM5_ESM.pdf]

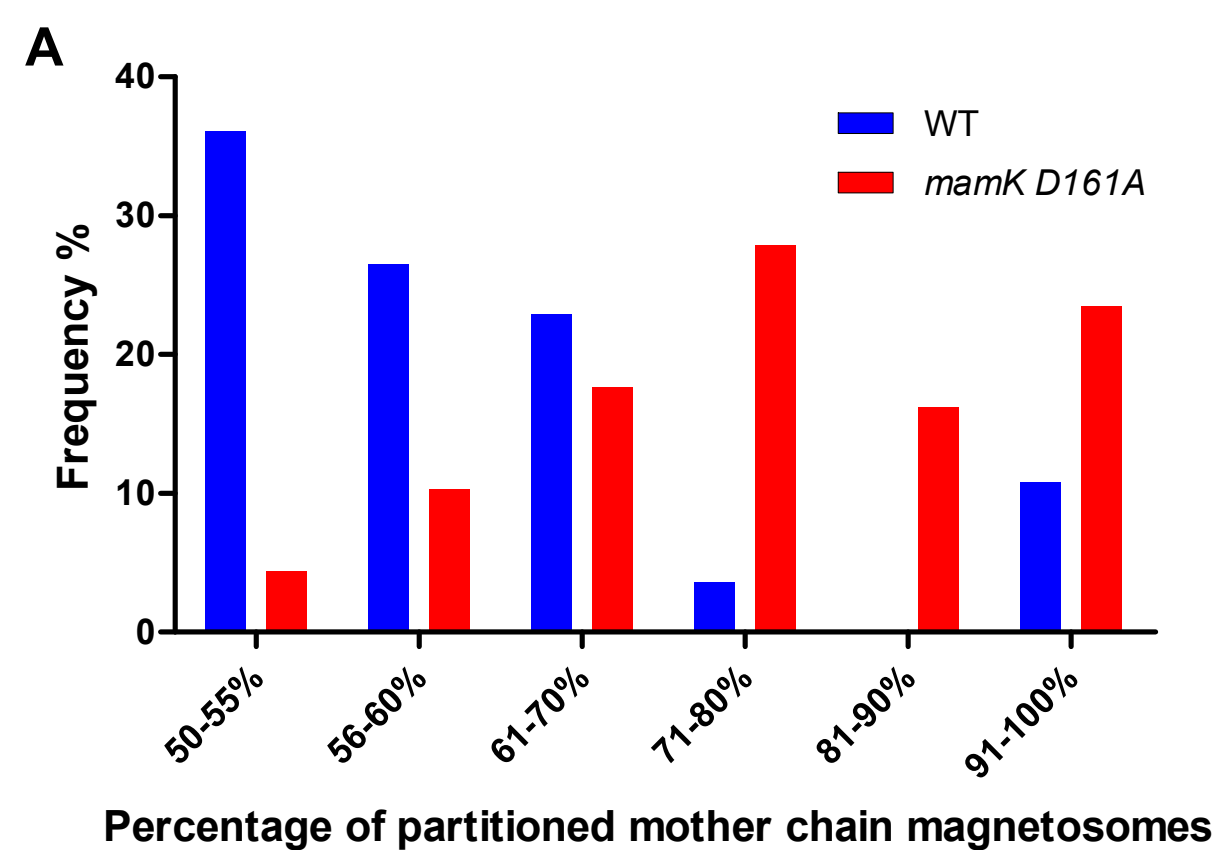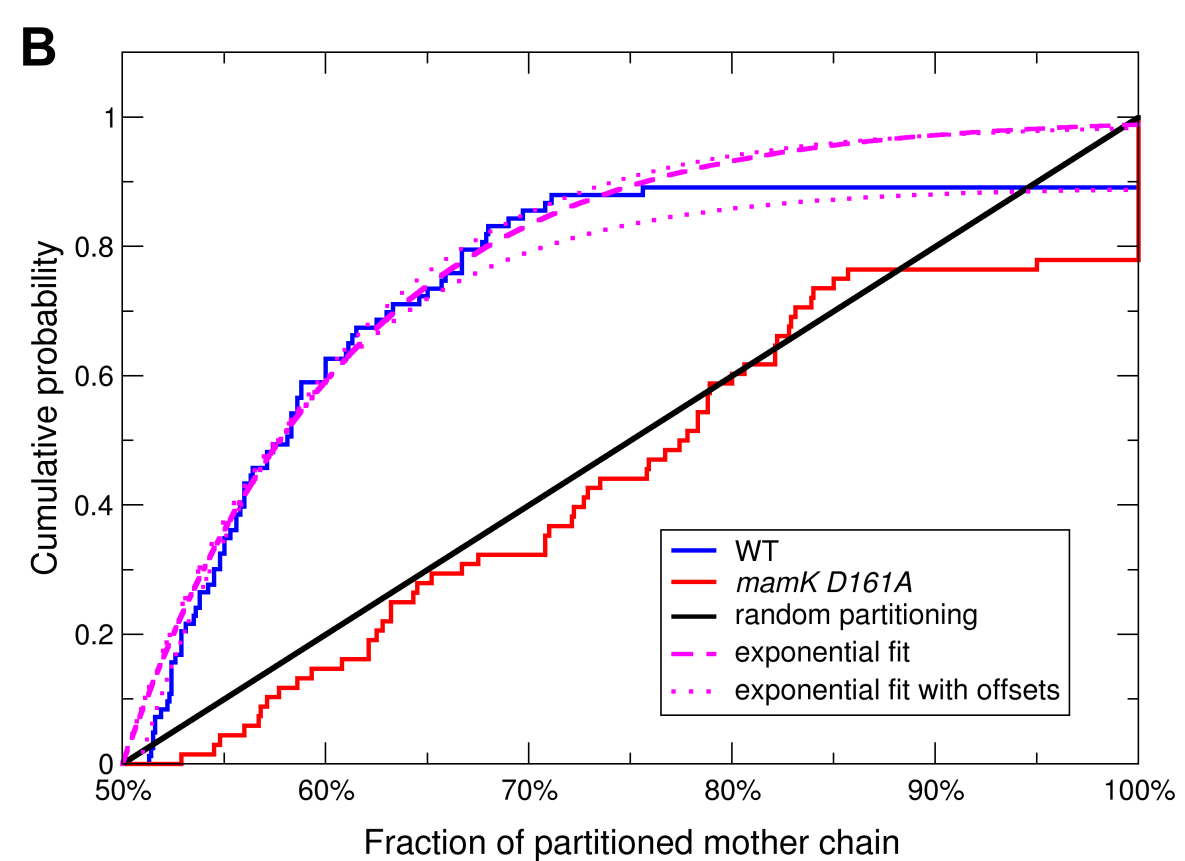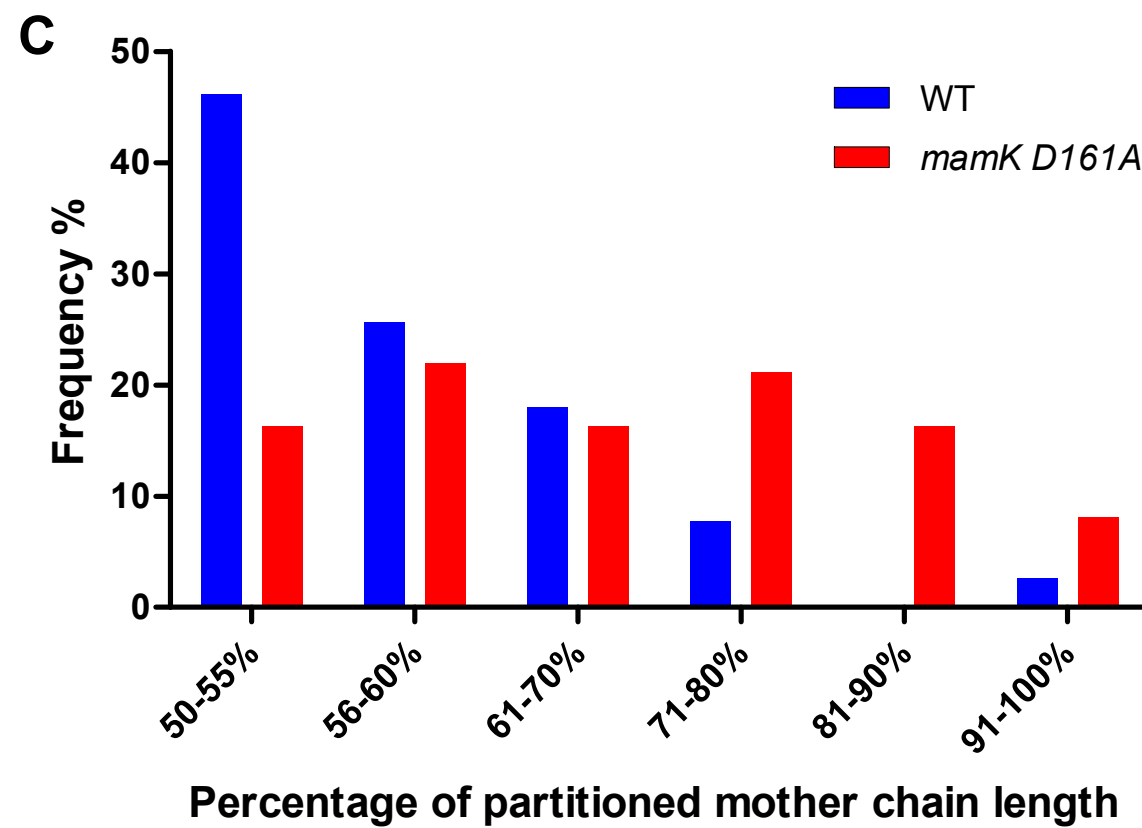

Supplement: Additional file 6: Figure S3. — Magnetosome chain (MC) segregation. (A) Quantification of the MC segregation in terms of magnetosomes per cell to be inherited by future daughter cells (wildtype (WT): n = 83; mamK D161A: n = 68). Cells were incubated under 21 % oxygen for 5 h. (B) Cumulative distribution of MC segregation data in “A”. (C) Quantification of the MC segregation length to be inherited by future daughter cells (WT: n = 39; mamK D161A: n = 123). Cells were grown under microoxic conditions (2 % oxygen). (PDF 200 kb) [file 12915_2016_290_MOESM6_ESM.pdf]

**A**

low diffusive mobility

high diffusive mobility

WT

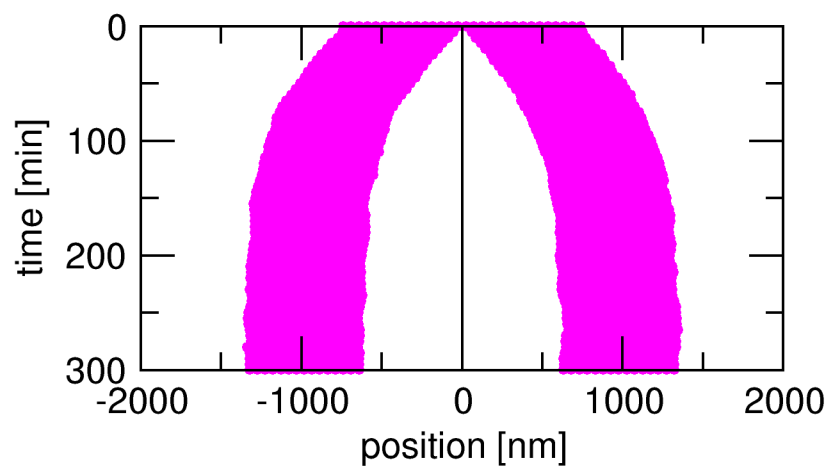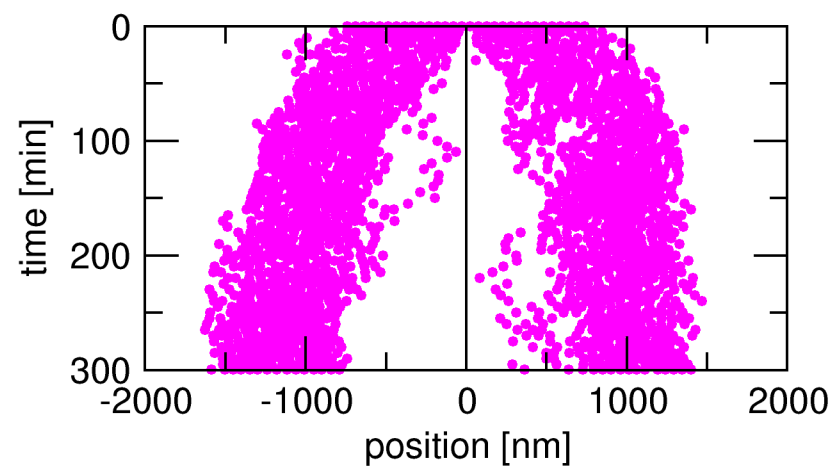**B**reduced  $v_0$ 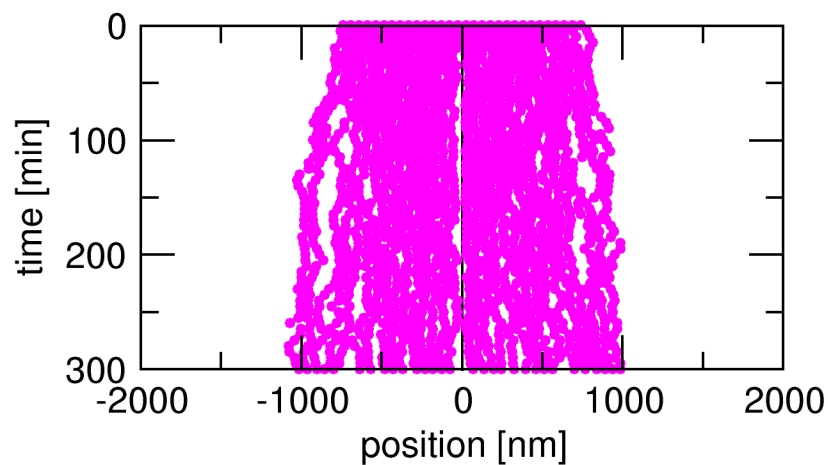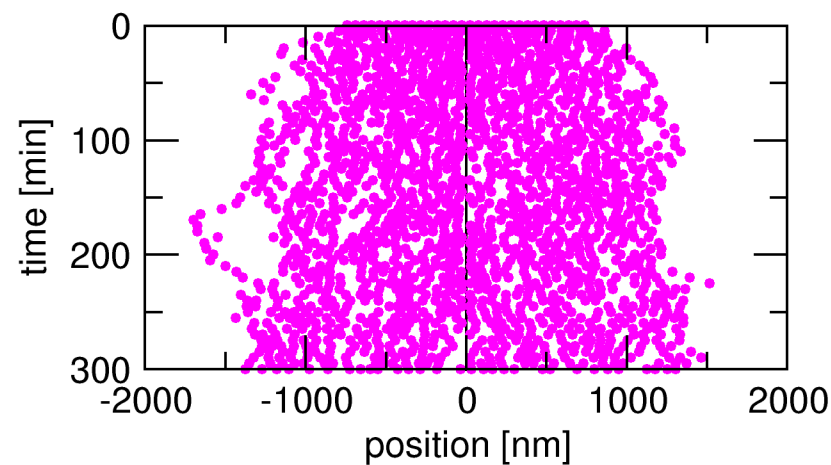**C**reduced  $F_s$ 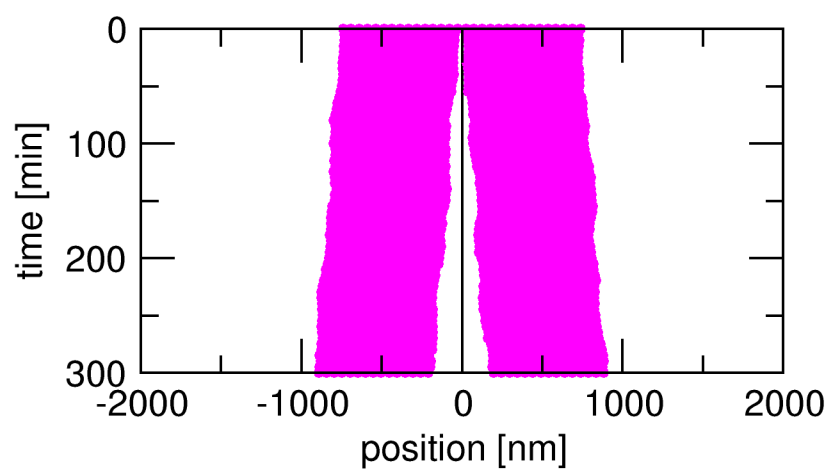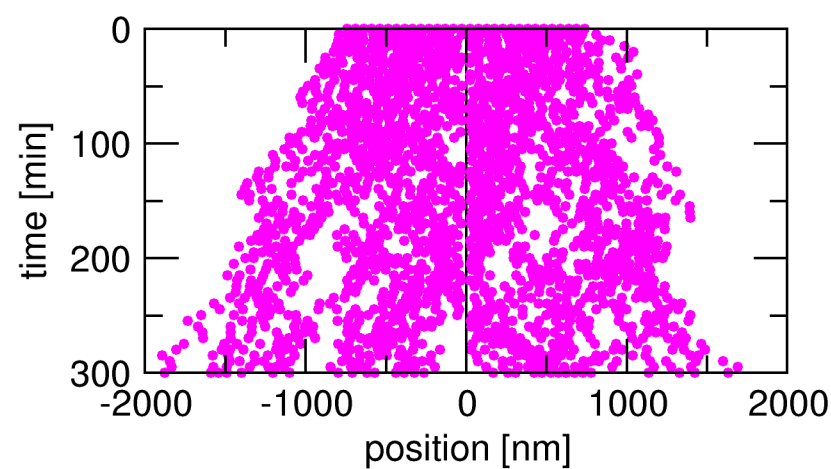

Supplement: Additional file 7: Figure S4. — Simulations of magnetosome chain (MC) dynamics. (A) MC motion after cell division was modeled under low and high diffusive mobility varying (B) velocity (V0) or (C) force (Fs). (PDF 261 kb) [file 12915_2016_290_MOESM7_ESM.pdf]

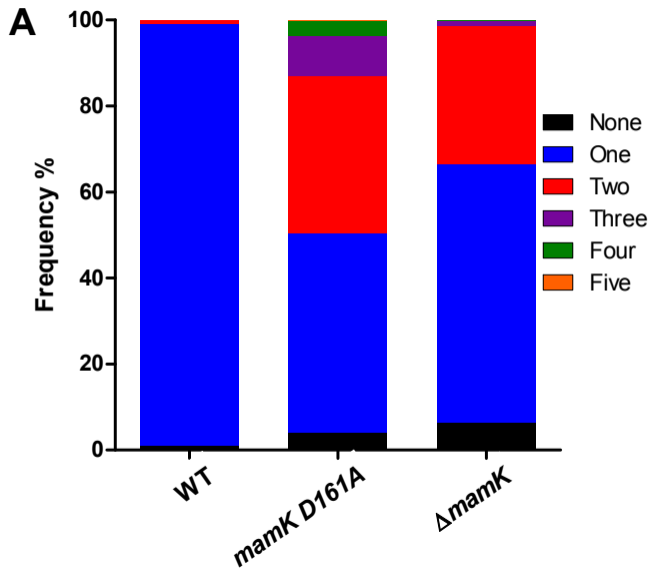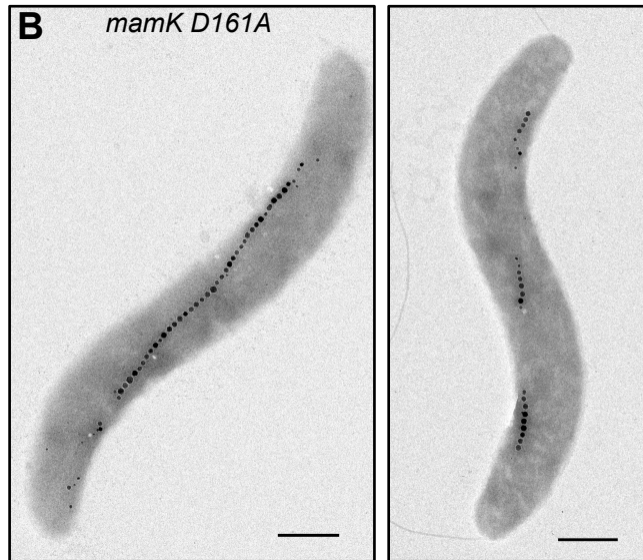

Supplement: Additional file 8: Figure S5. — Magnetite crystal size and mamK D161A strain phenotypes. (A) Quantification of magnetosome chains (MCs) per cell under microoxic growth (2 % oxygen) in Magnetospirillum gryphiswaldense MSR-1 wildtype (n = 298), mamK D161A (n = 521) and ∆mamK (n = 469). (B) TEM micrographs of mamK D161A displaying wildtype-like (left panel) or fragmented (right panel) MCs. Scale bars: 1 μm. (PDF 1828 kb) [file 12915_2016_290_MOESM8_ESM.pdf]

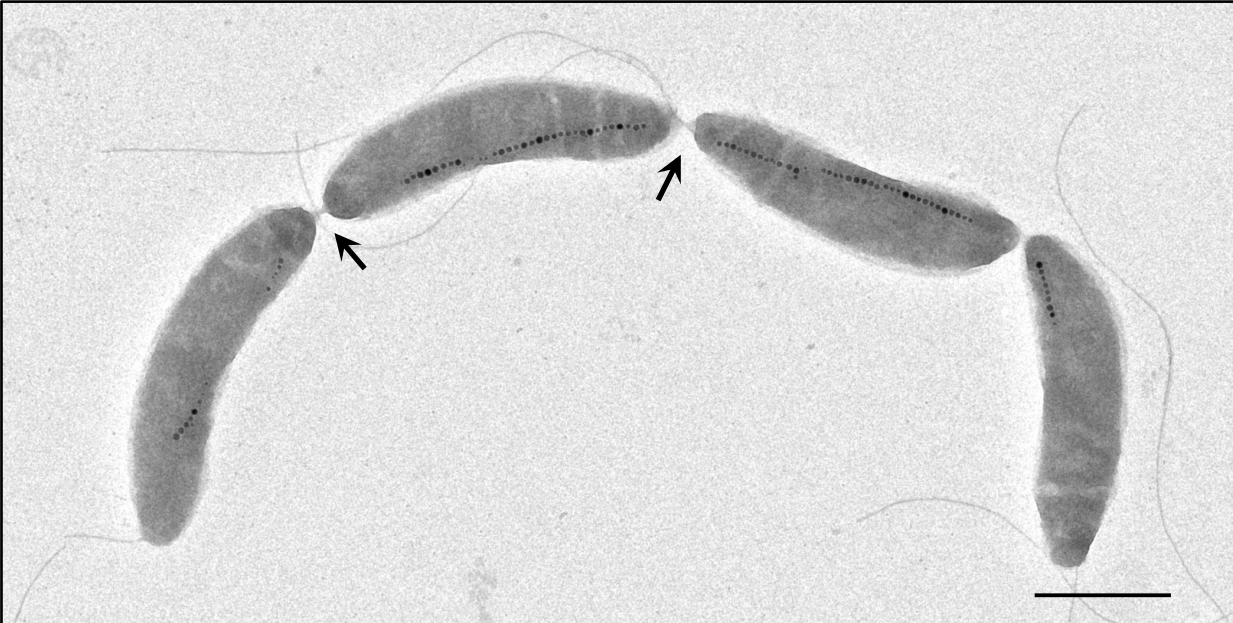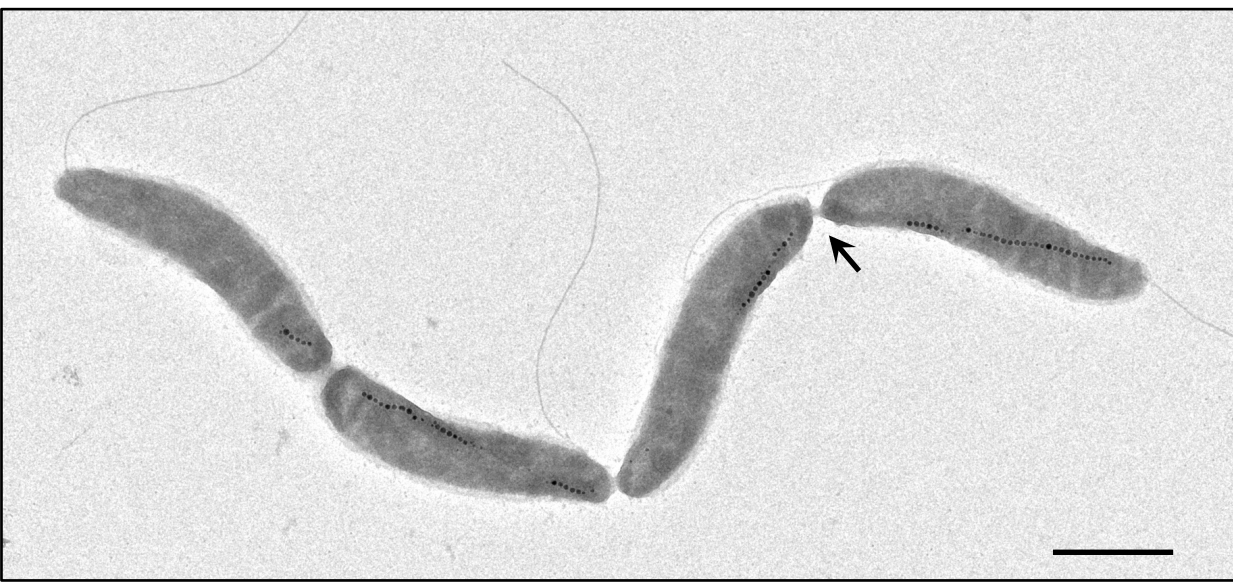

Supplement: Additional file 9: Figure S6. — Connected cells of mamK D161A strain. TEM micrographs of mamK D161A displaying four cells that seem completely divided, albeit still connected by some membranous bridging structures (indicated by arrows) and suggested to be held by MamKD161A stable filaments. Scale bars: 1 μm. (PDF 1014 kb) [file 12915_2016_290_MOESM9_ESM.pdf]

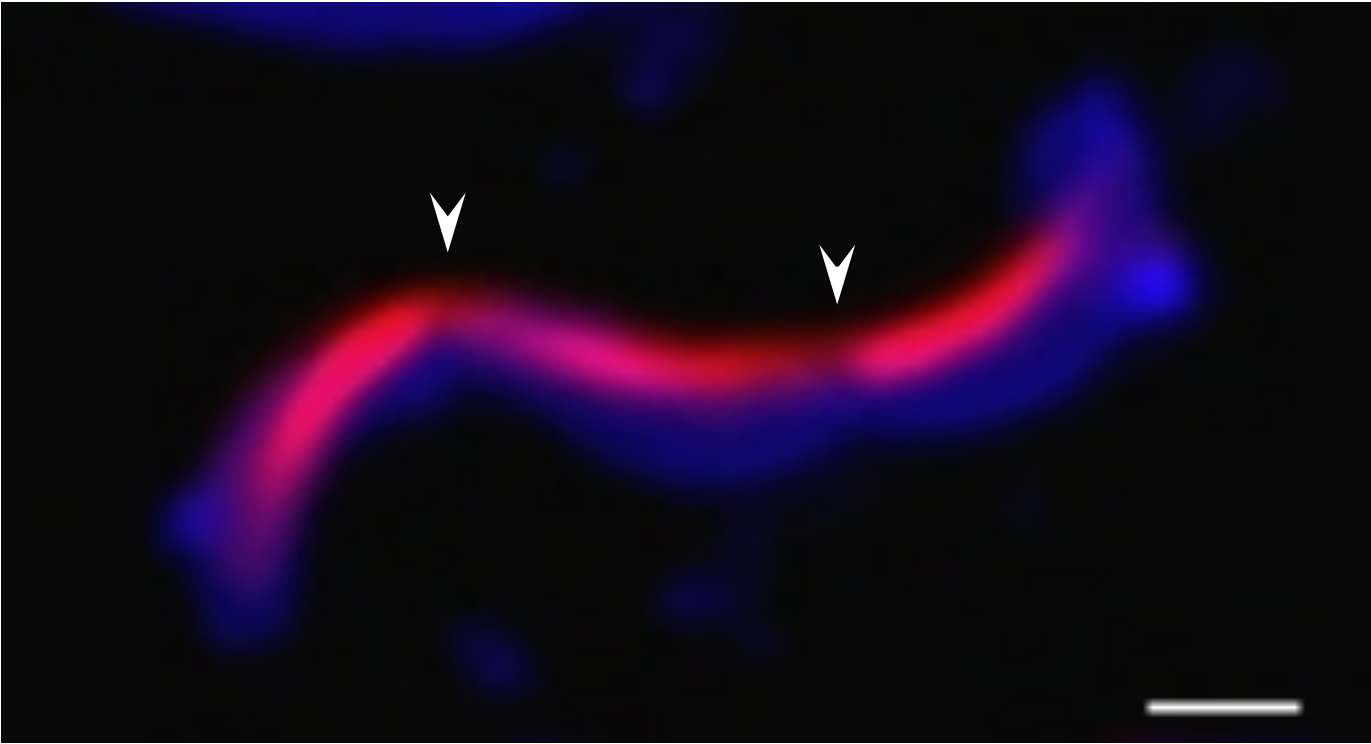

Supplement: Additional file 10: Figure S7. — Fluorescence microscopy of mCherry-MamKD161A filament in connected cells. Cell membrane was stained with the fluorescent dye CellBriteTM Blue. Arrows indicate the cell division site constriction where the mCherry-MamKD161A filament signal appears to cross. Scale bar: 1 μm. (PDF 16 kb) [file 12915_2016_290_MOESM10_ESM.pdf]

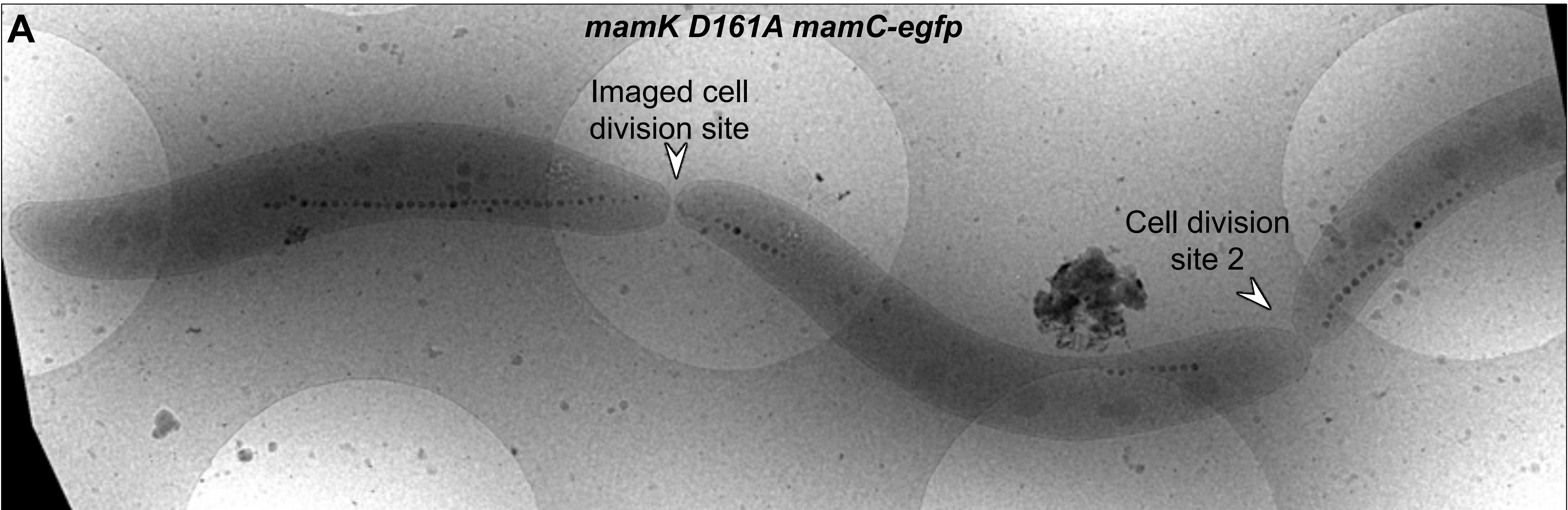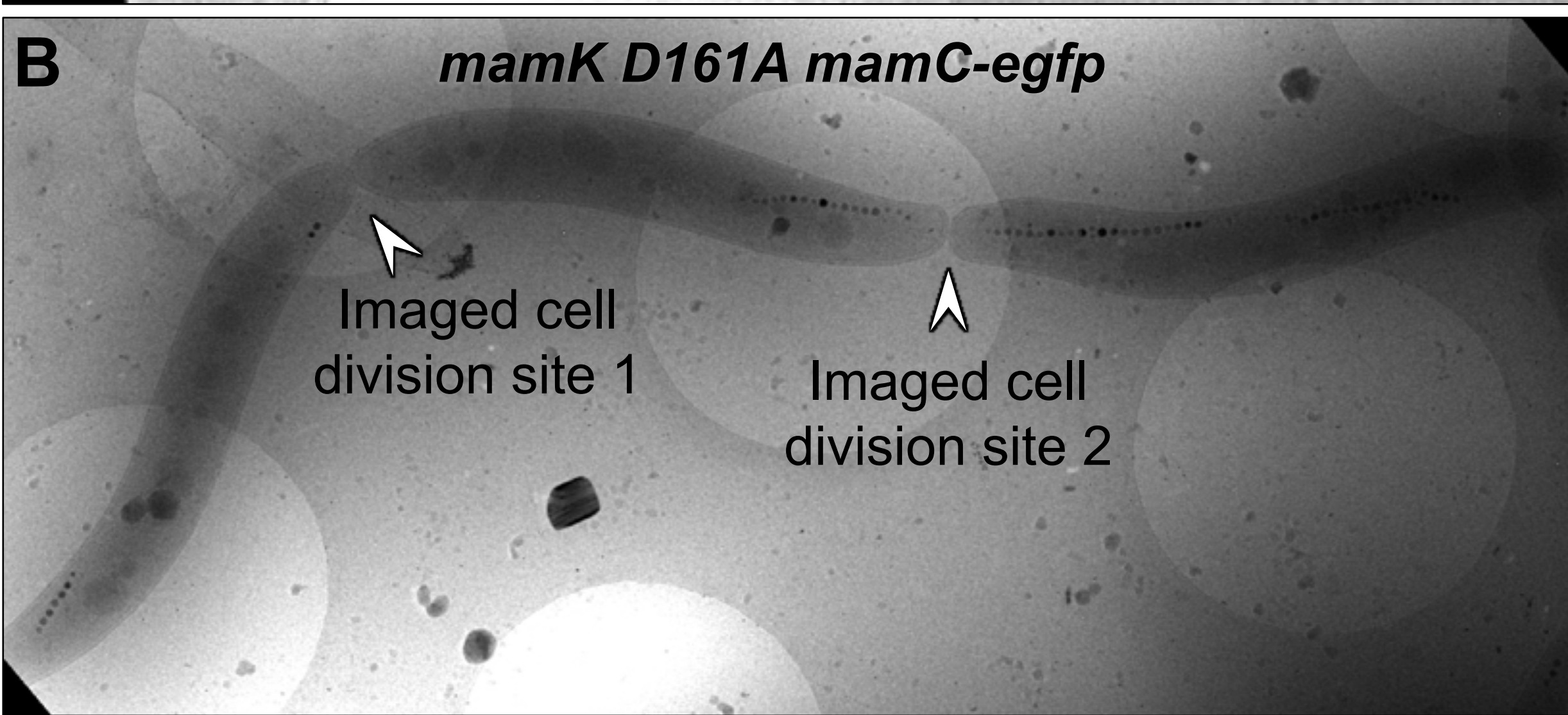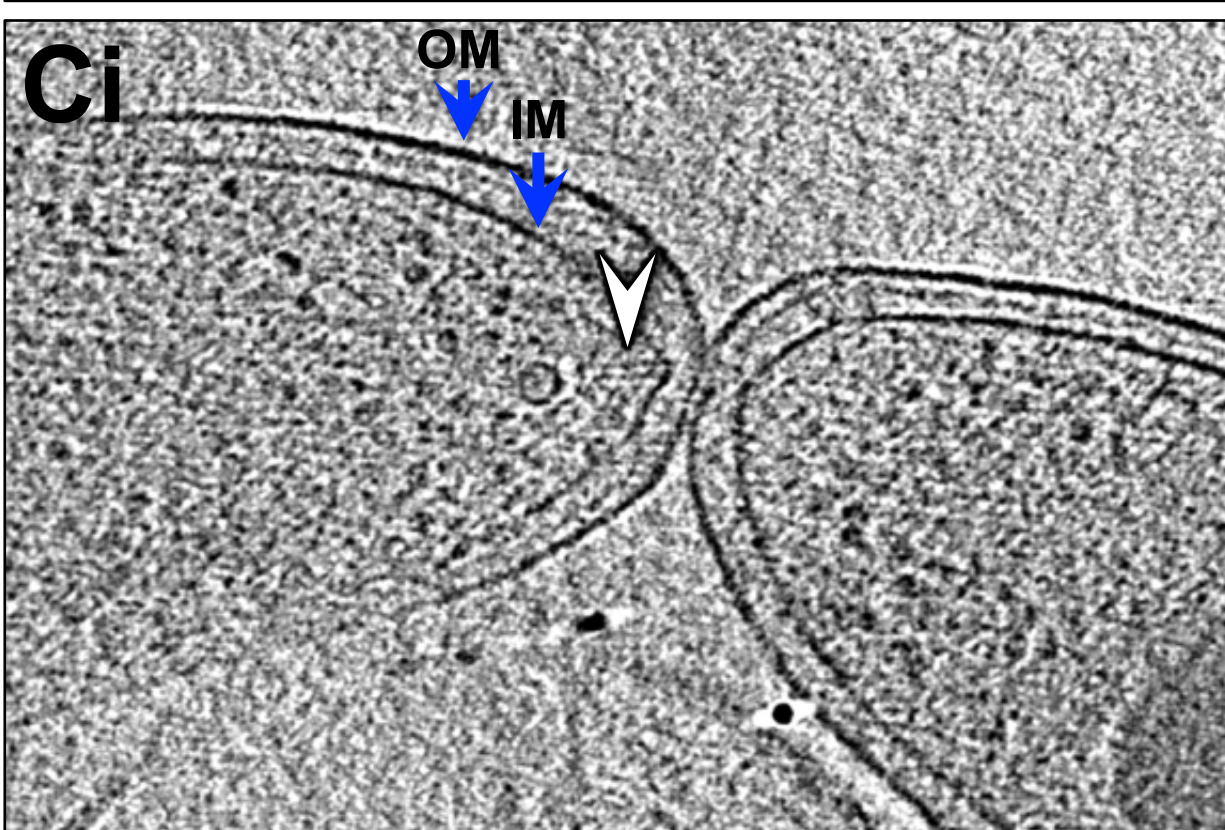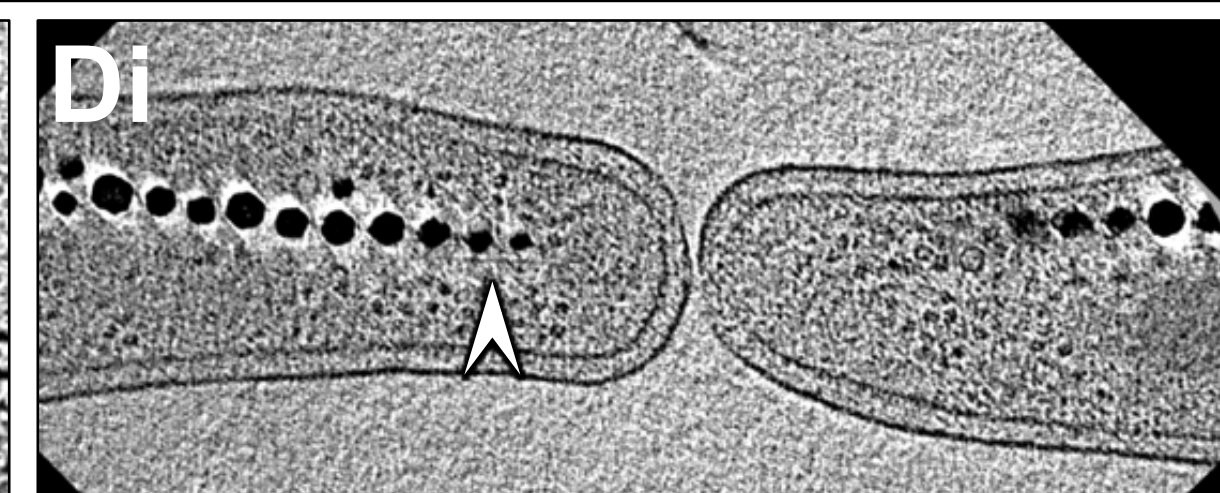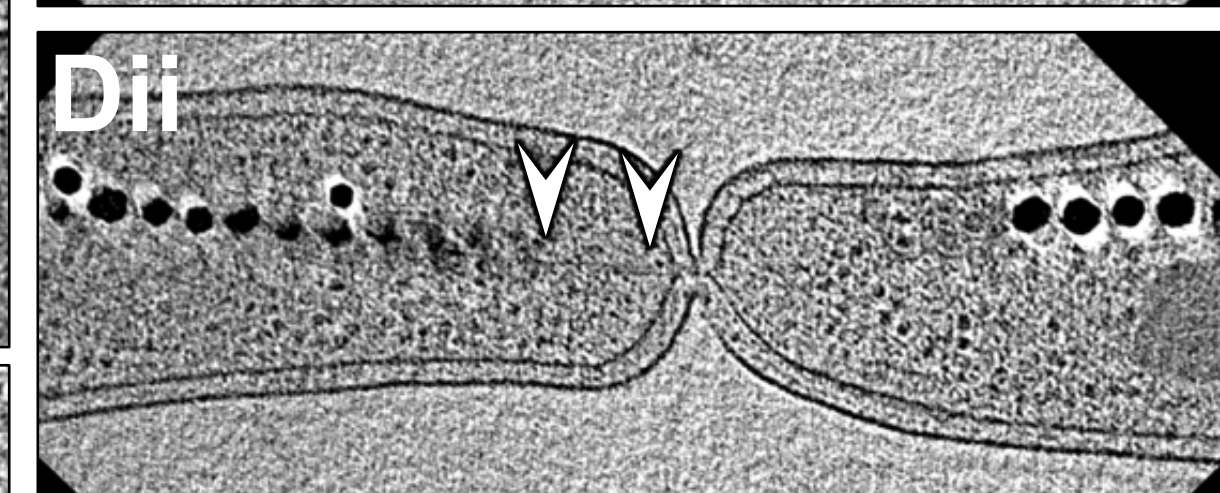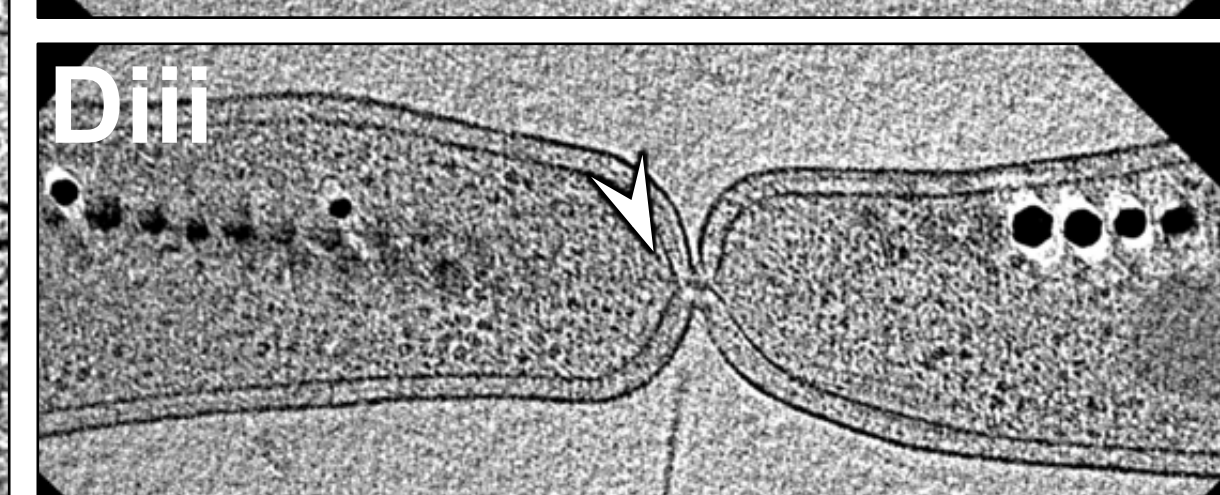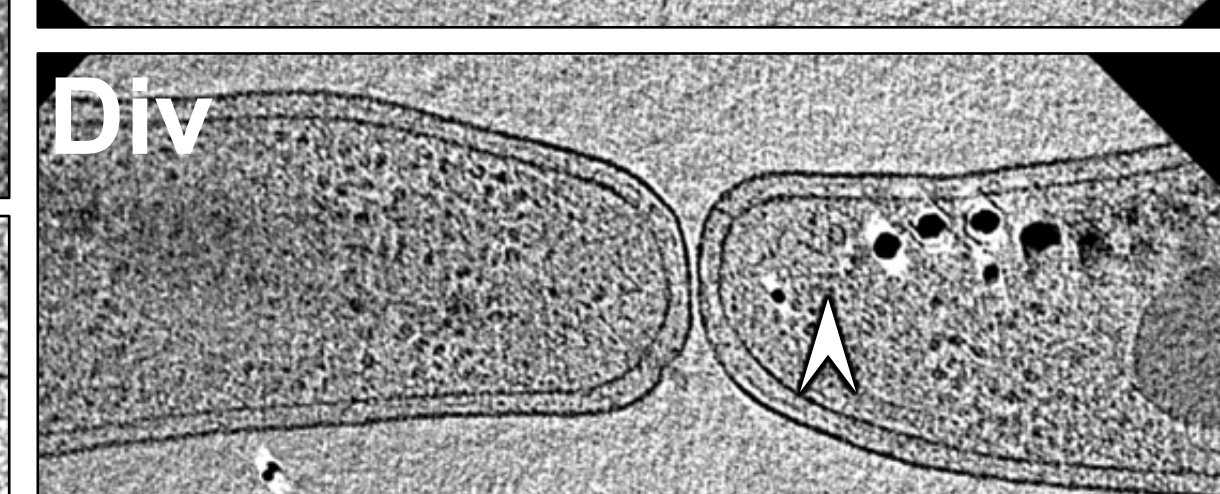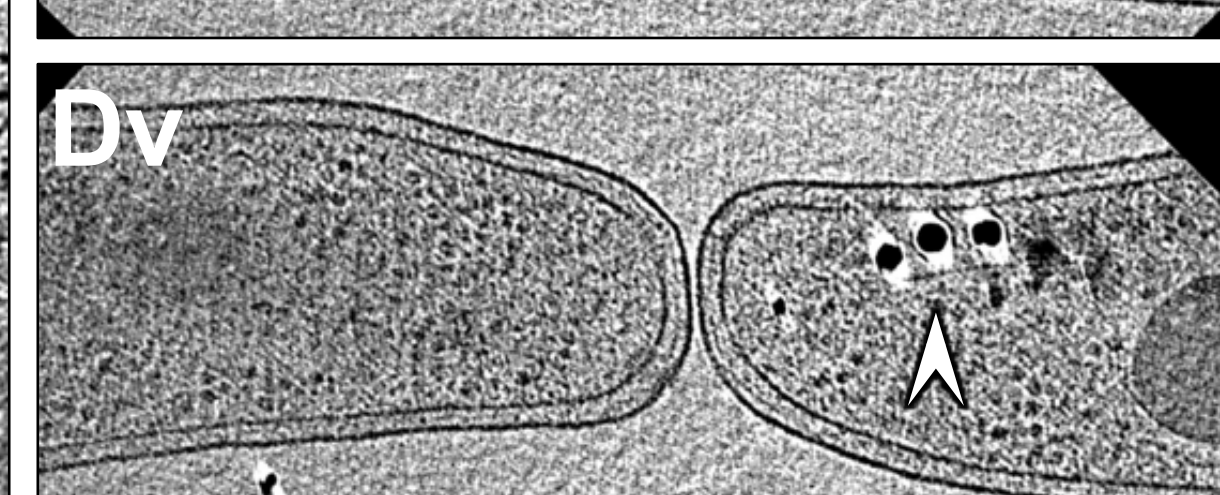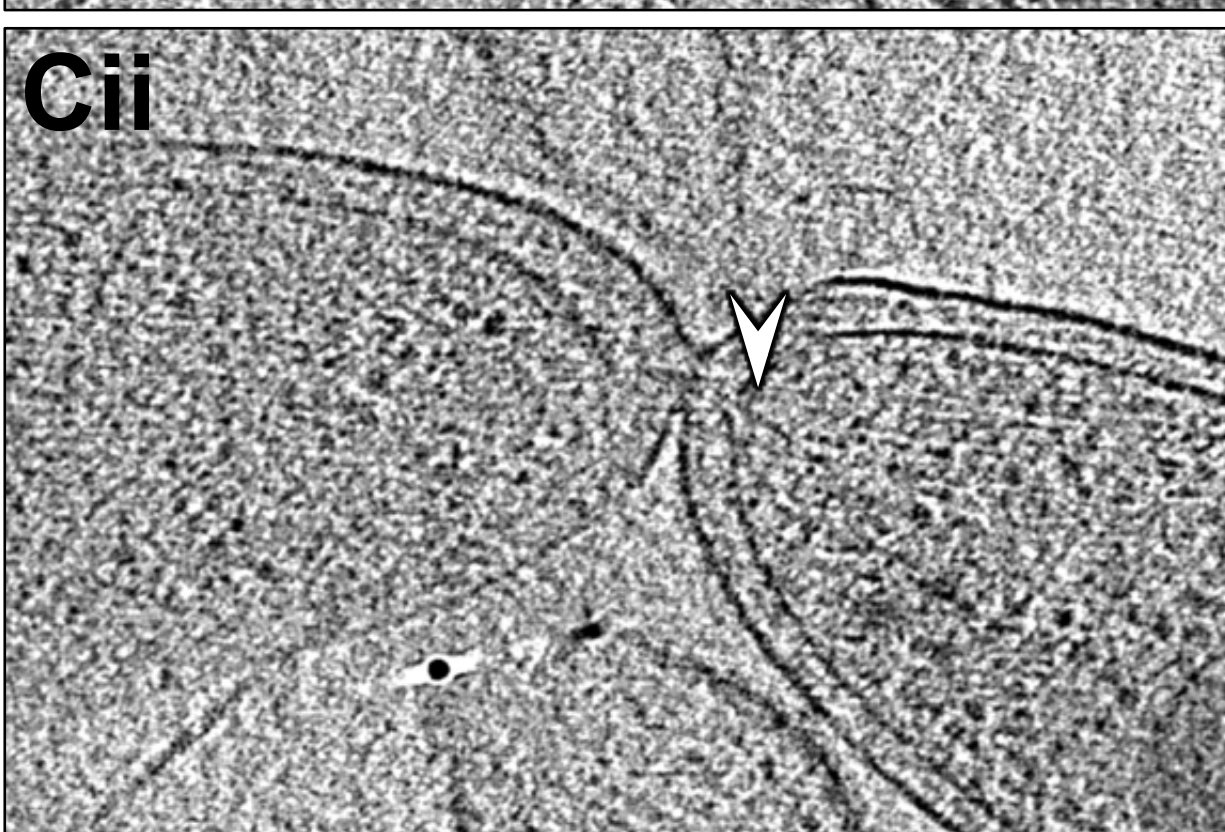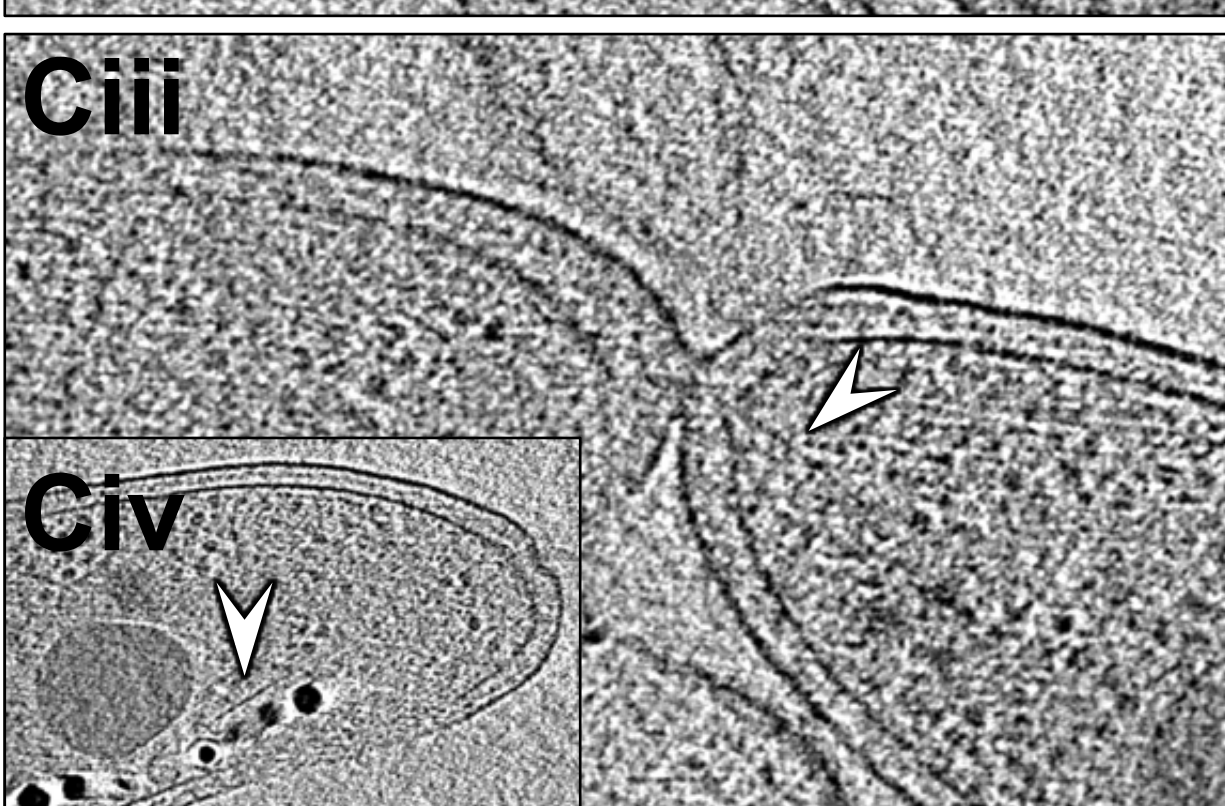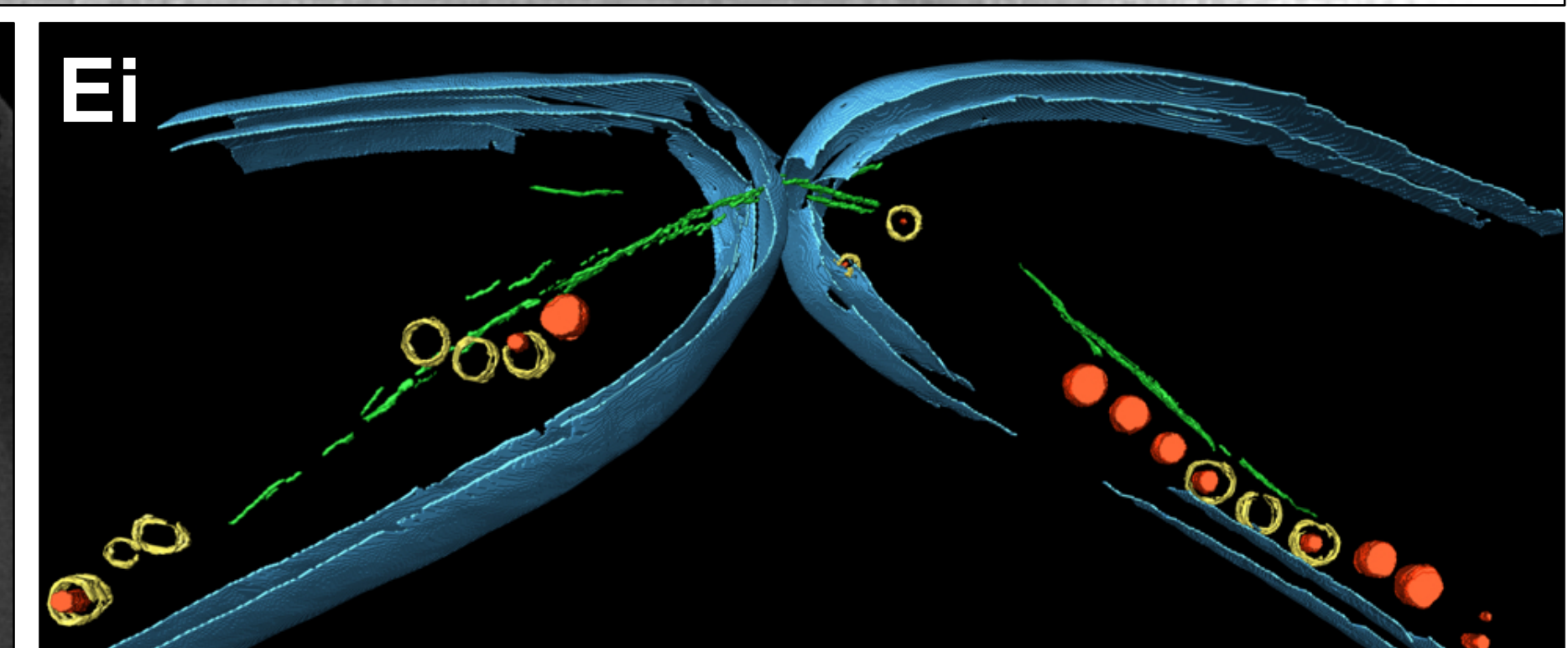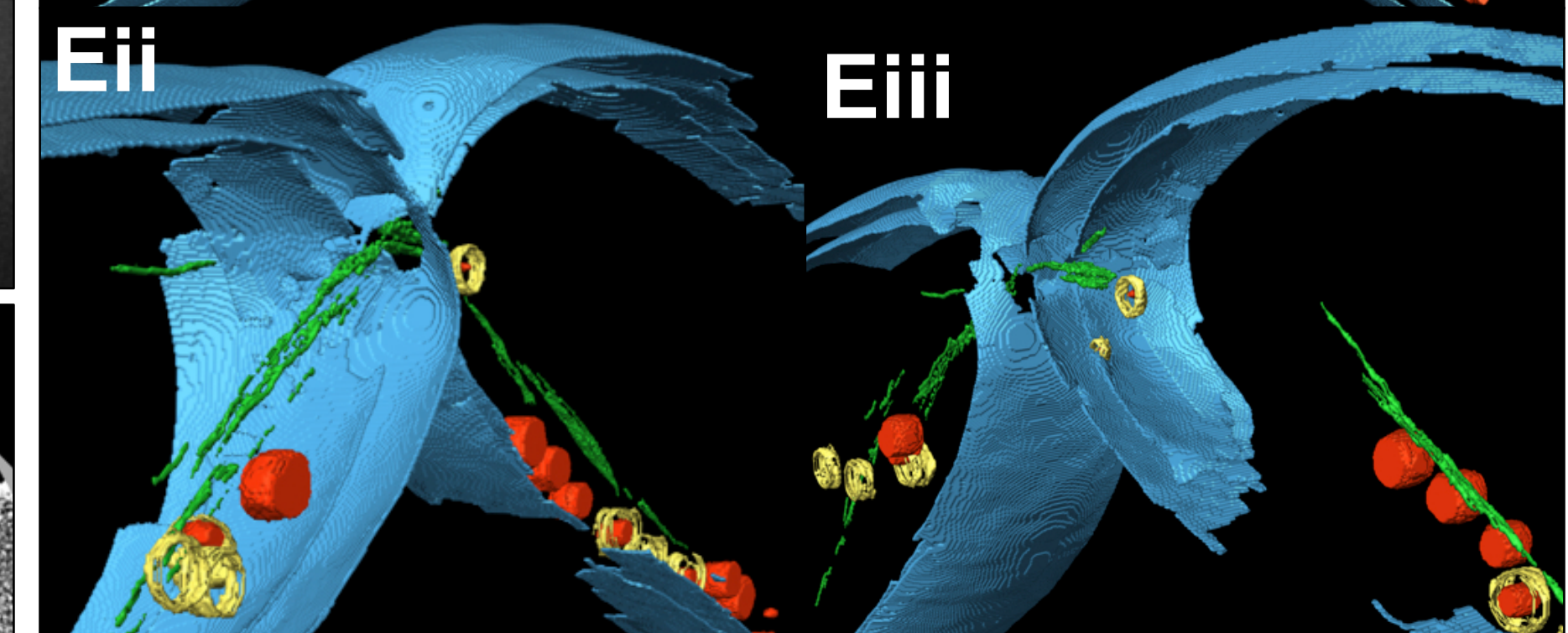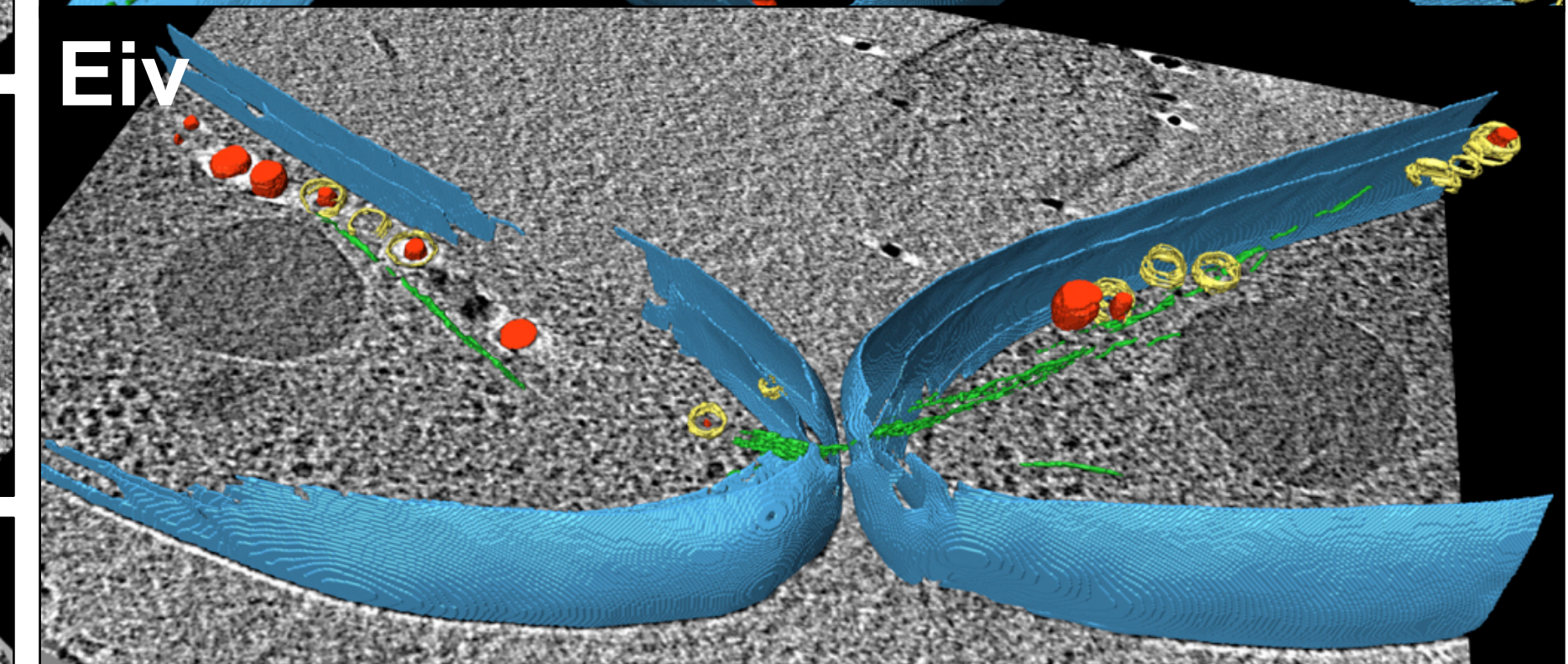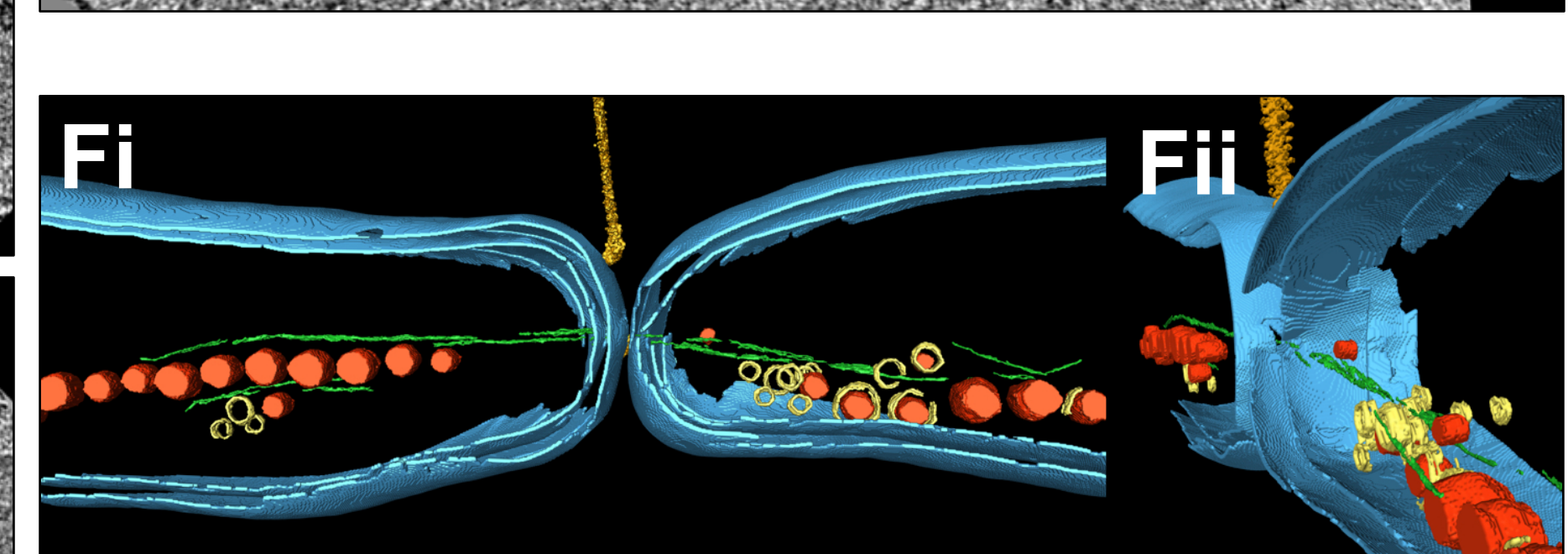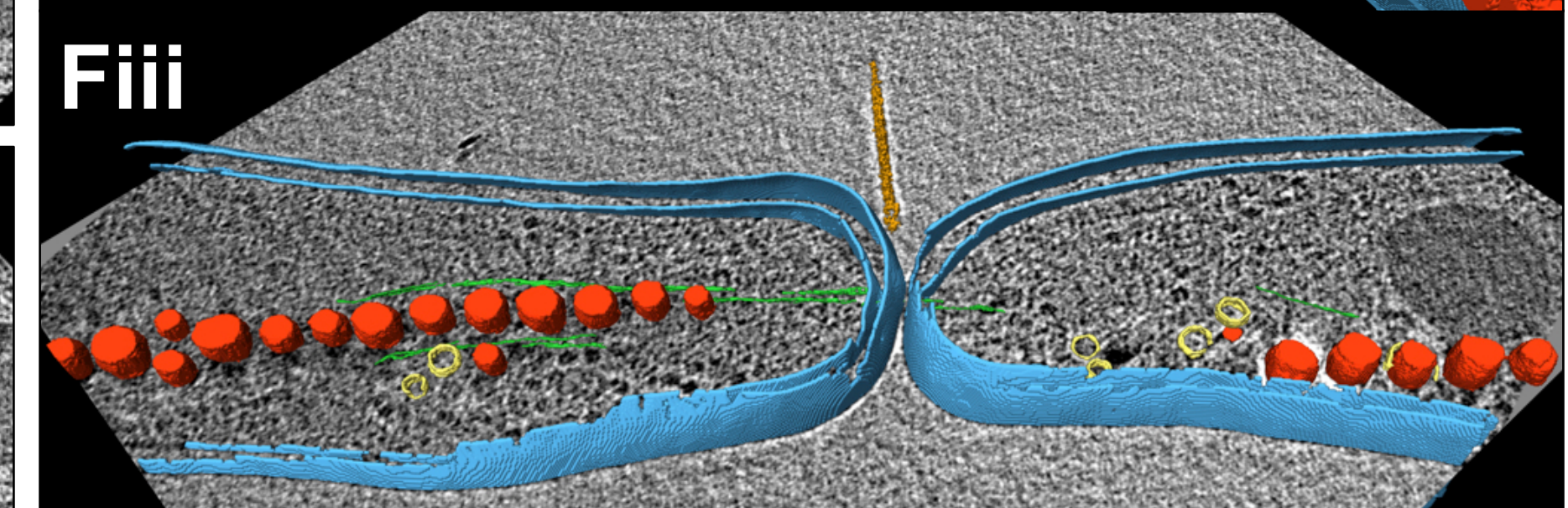

Supplement: Additional file 11: Figure S8. — Cryo-electron tomography (CET) of cell division sites of mamK D161A connected cells. (A) CET micrograph of three connected cells. The indicated cell division site (placed on the grid’s hole) was selected for tomography (imaged cell division site in Fig. 4b–d). (B) CET micrograph of three connected cells. Both cell division sites (arrowheads) were suitable for tomography. (Ci-Civ) CET sections of cell division site 1, (Di-Dv) and cell division site 2 as indicated in “B”. White arrowheads indicate MamK filaments entering and exiting the membranous bridge. Blue arrows indicate inner (IM) and outer (OM) membranes. (Ei-Eiv) CET 3D rendering of the cell division site 1, (Fi-Fiii) and cell division site 2 indicated in “B”. Magnetite crystals: red. Vesicles: yellow. MamK filament: green. Cellular envelope, inner and outer membrane: blue. Flagella: gold. (PDF 4458 kb) [file 12915_2016_290_MOESM11_ESM.pdf]

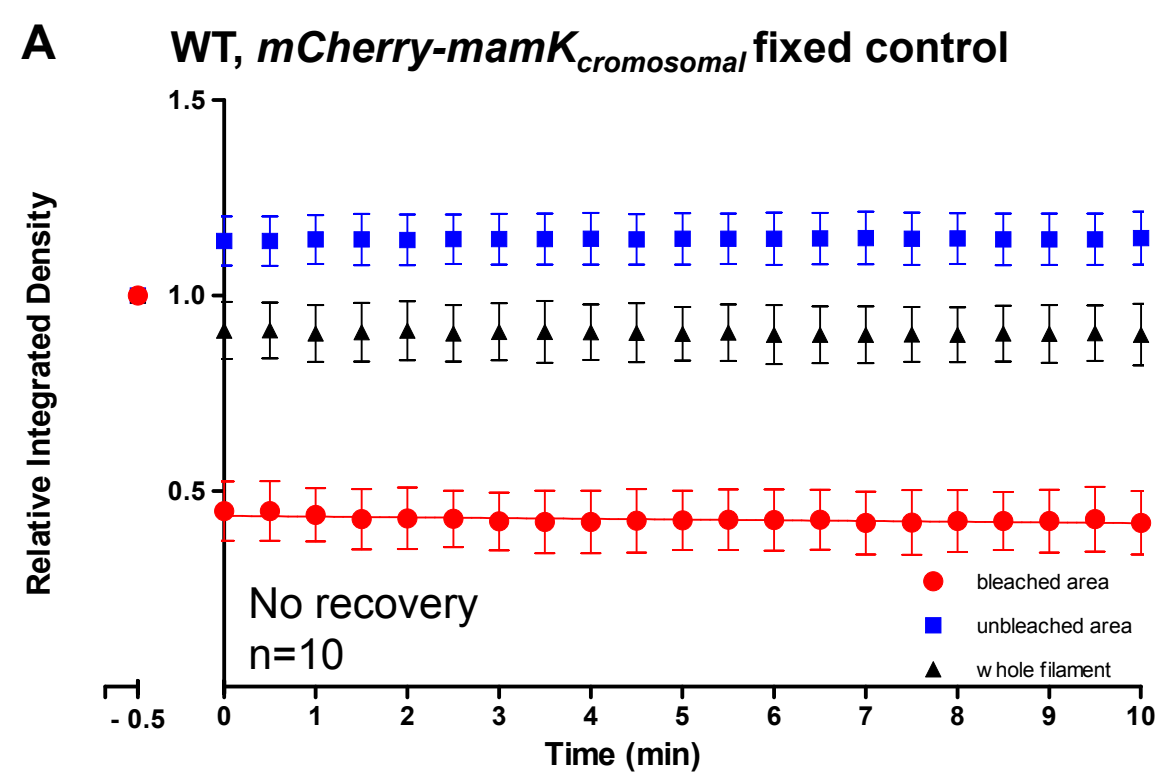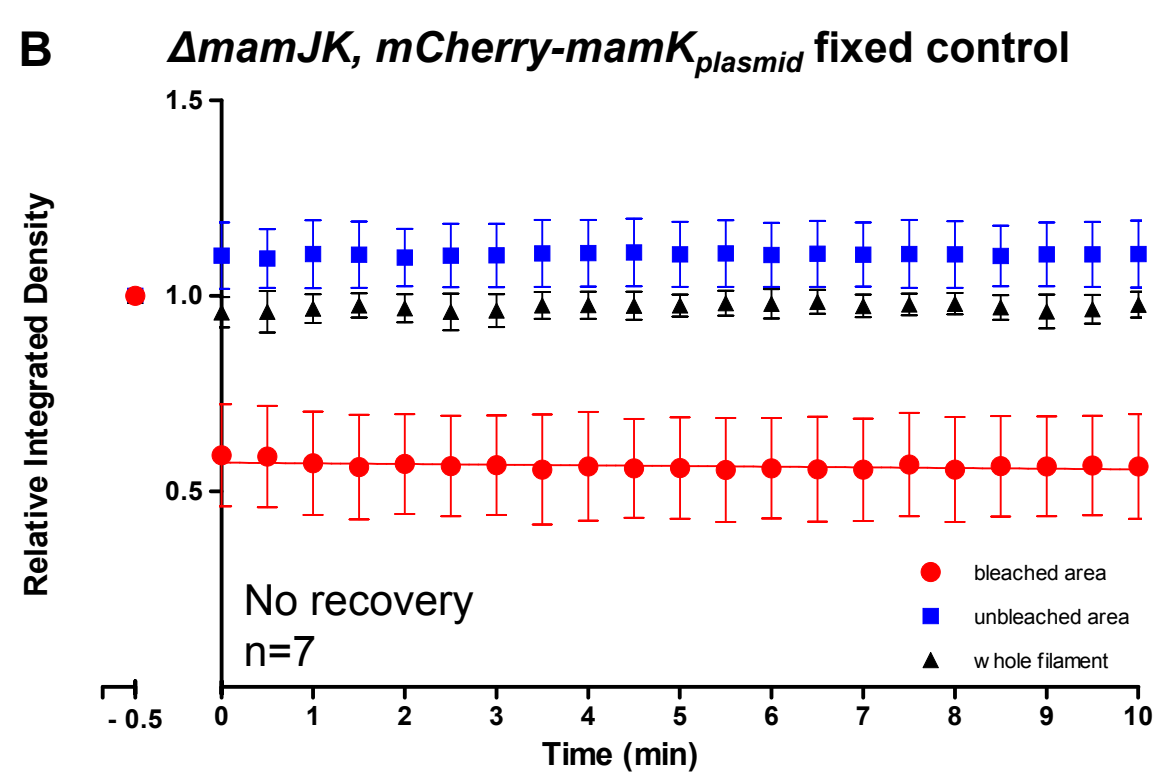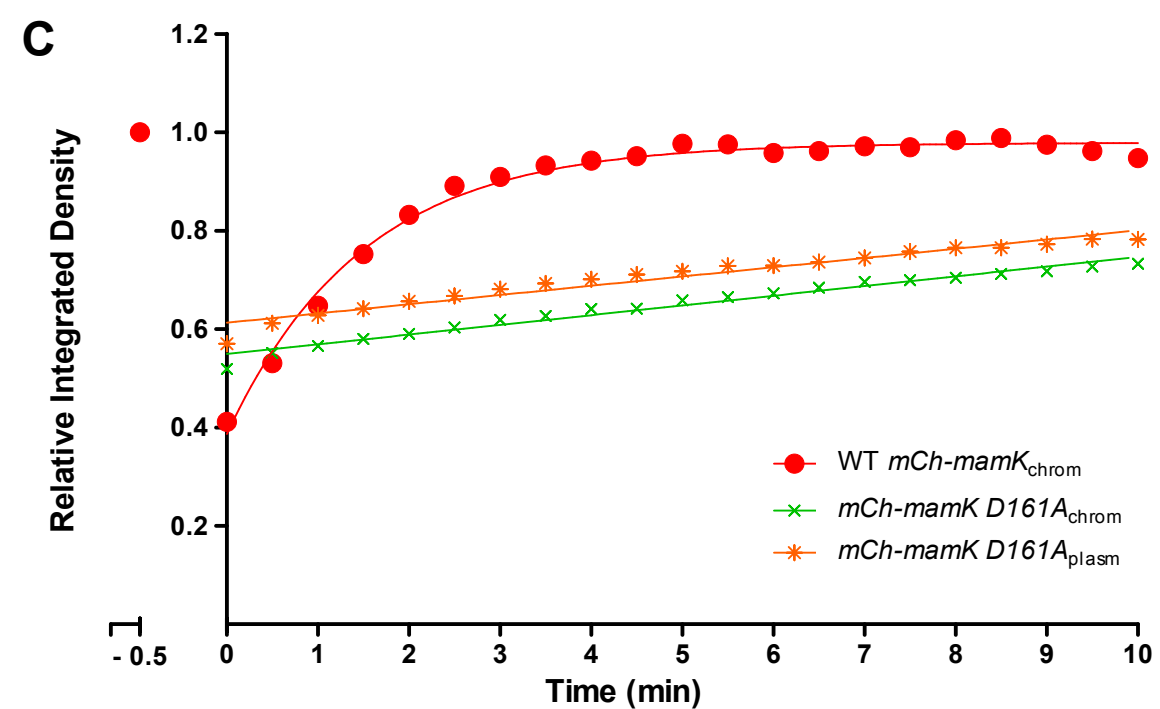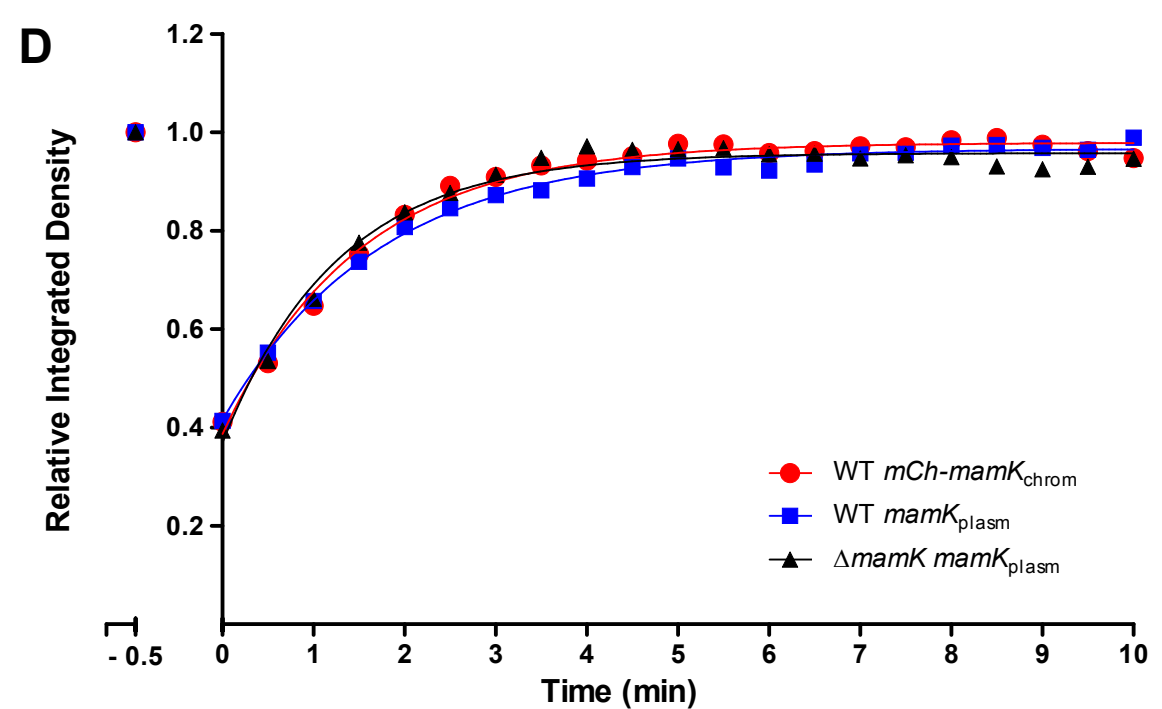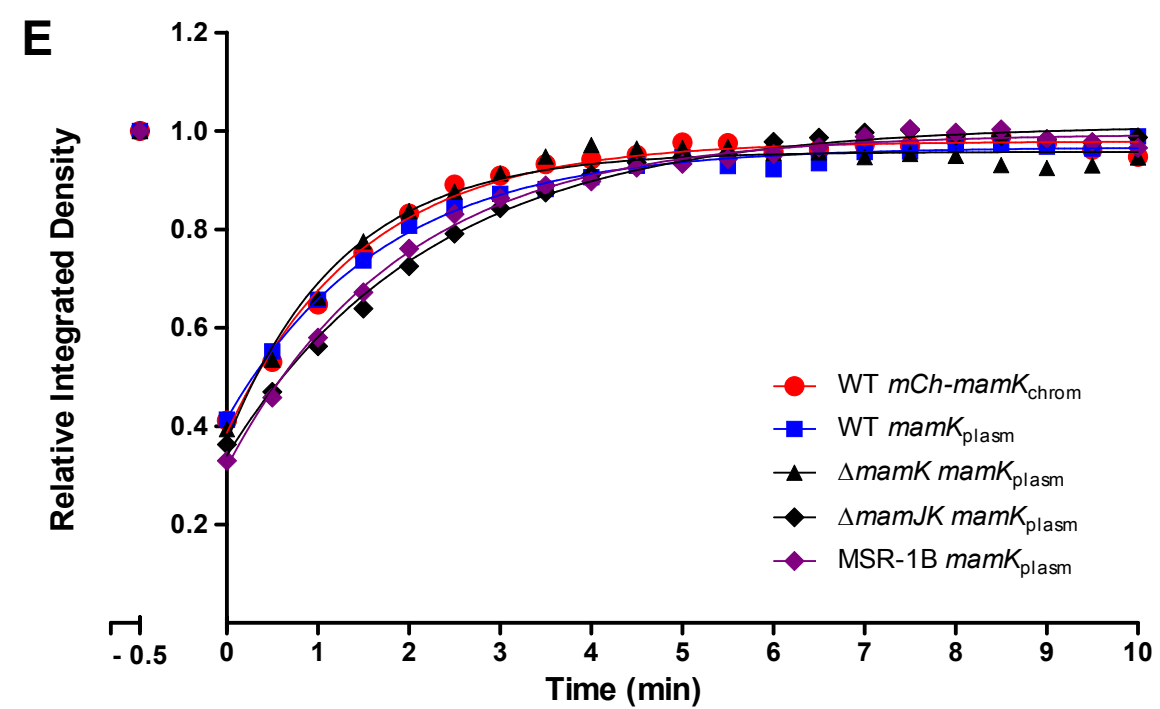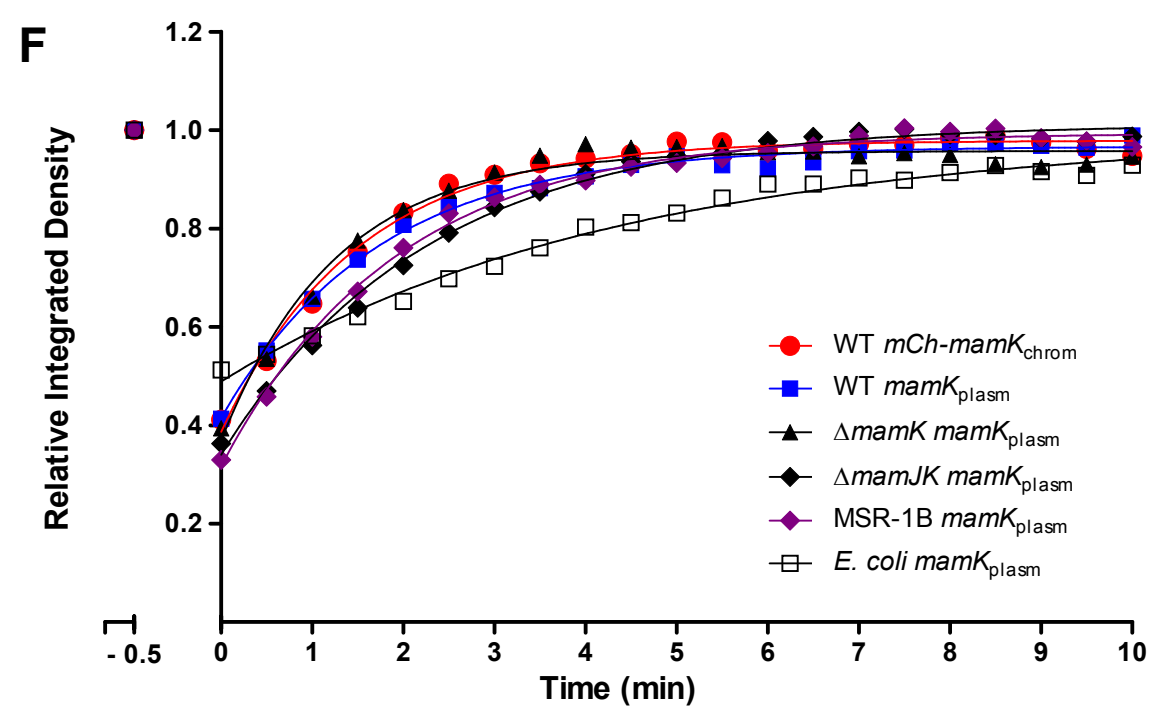

Supplement: Additional file 15: Figure S9. — Controls and curves of fluorescence recovery after photobleaching. Dark-state fluorophore reversal was controlled by fixing the cells with 1 % formaldehyde for 1 h and then FRAP was performed. Therefore, the translational fusion mCherry-MamK expressed from (A) the mamK locus or (B) a replicative plasmid in ∆mamJK background was evaluated in fixed cells. (C–F) Comparison of fluorescence recovery curves over the time after the laser application for different strains. Only bleached areas were plotted for each strain. Zero time was measured immediately after laser pulse. SD corresponding to each time point is not showed for a better comparison of the curves. (PDF 455 kb) [file 12915_2016_290_MOESM15_ESM.pdf]

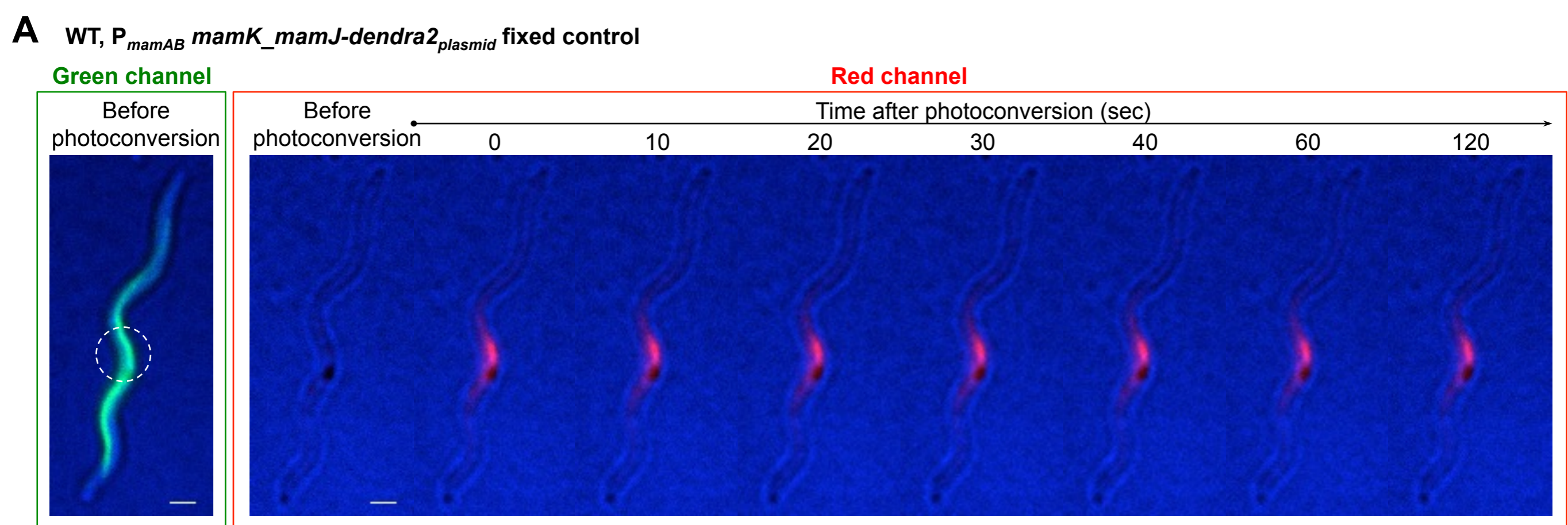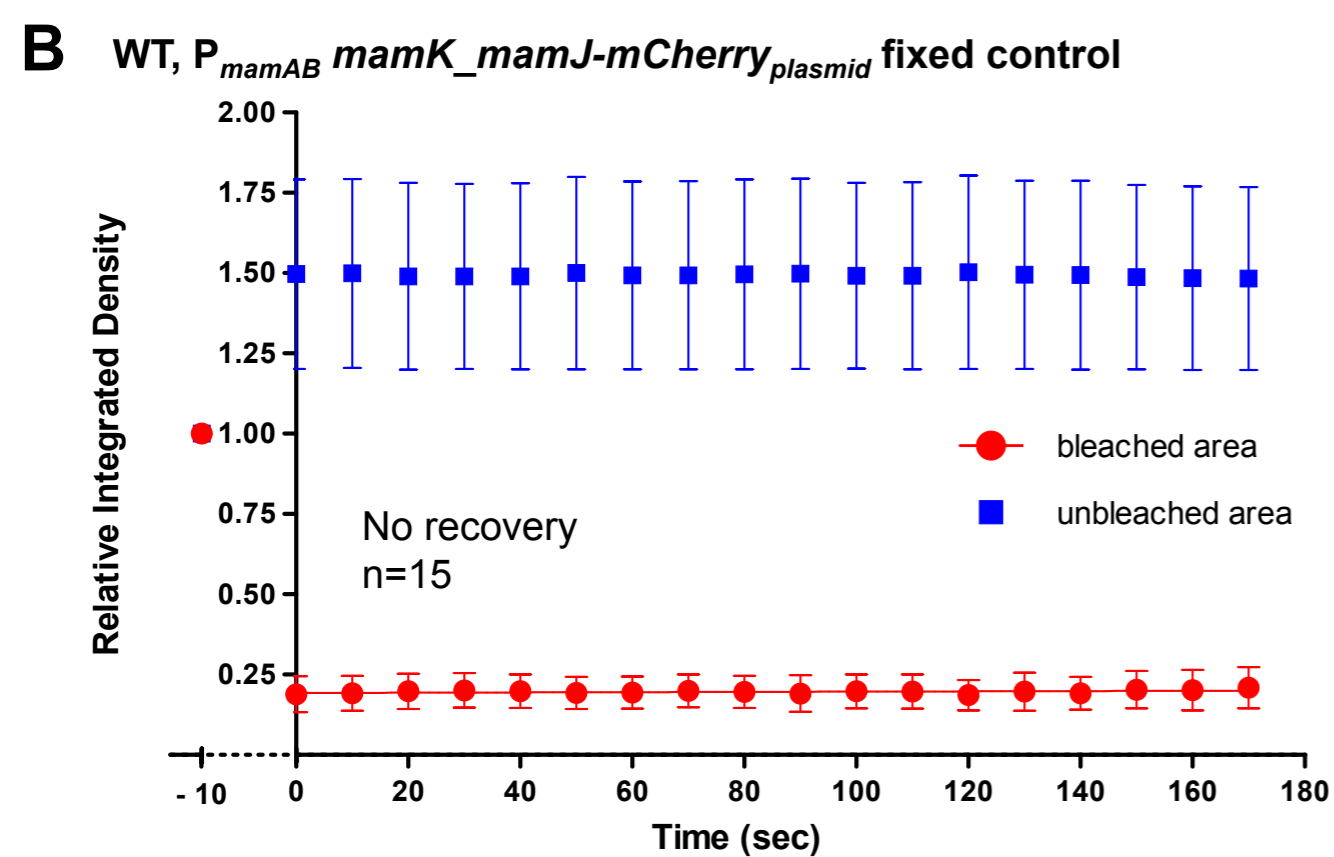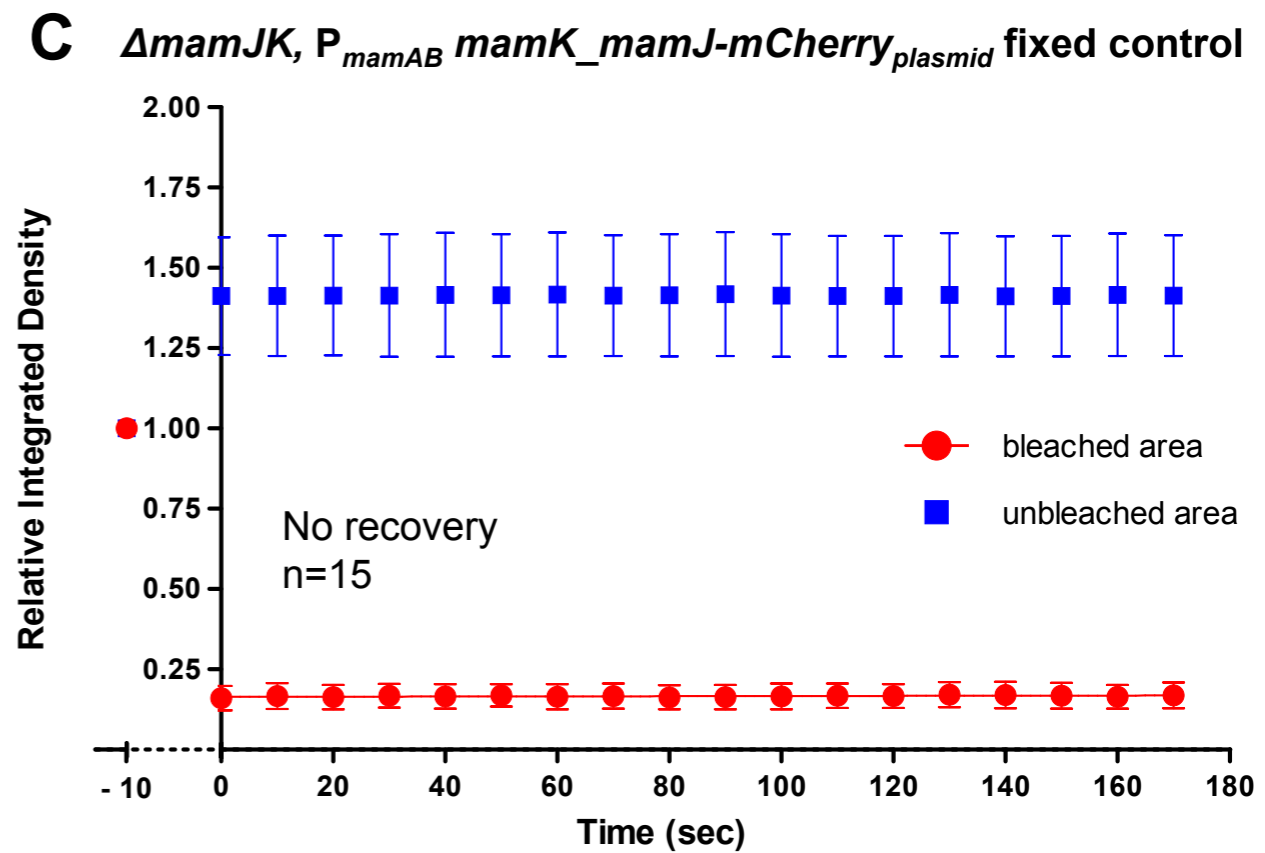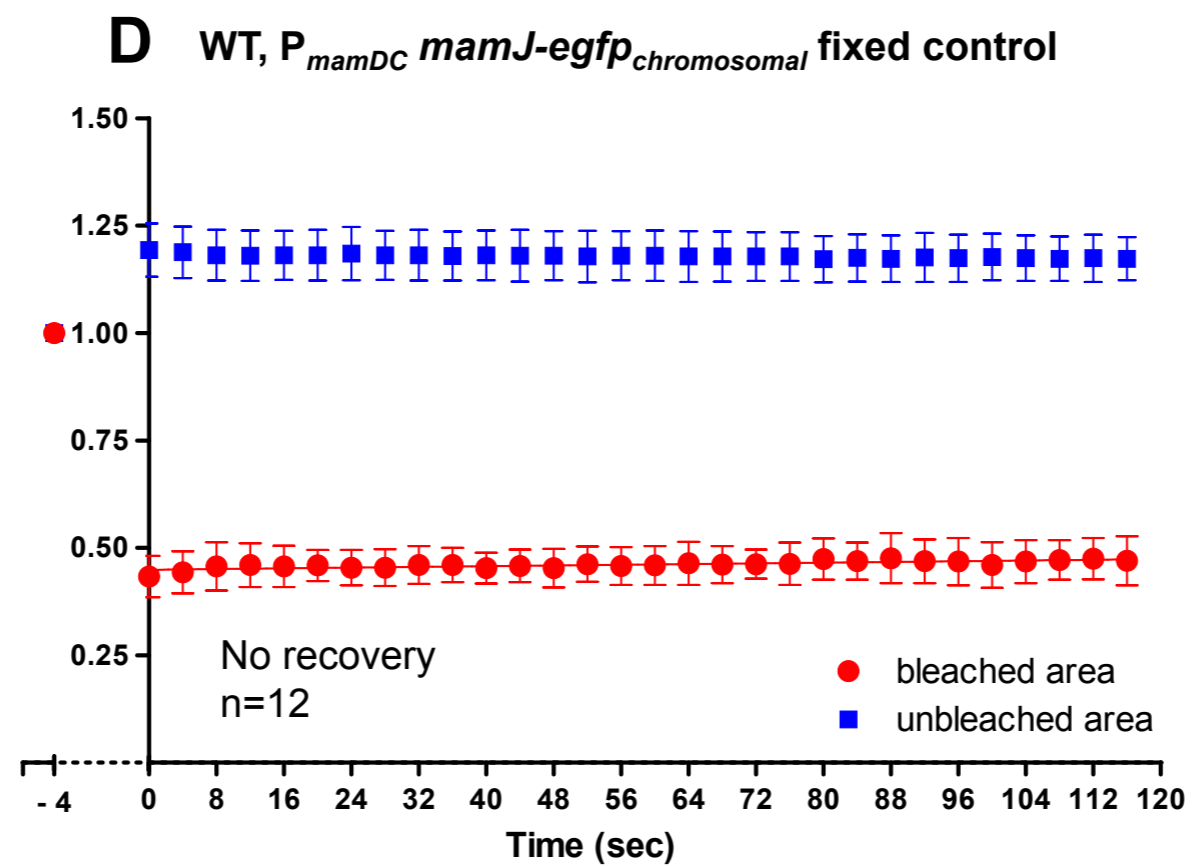

Supplement: Additional file 20: Figure S13. — Controls of MamJ dynamics assays. (A) Representative cell of a fixed control of MamJ-Dendra2 photoconversion co-expressing mamK (n = 4) in MSR wildtype (WT) strain. Green channel displays the filament prior to photoconversion. Red channel shows photoconverted protein after a laser line 405 nm pulse application. (B) Control for dark-state-reversal of mCherry fluorophore fused to MamJ and co-expressing mamK in MSR WT (n = 15) and (C) ∆mamJK strains (n = 15). (D) Control for dark-state-reversal of MamJ-EGFP fusion expressed under the control of the mamGFDC operon (located in the MAI) promoter (PmamDC) from a Tn5-based chromosomal insertion in the MSR WT strain (n = 12). Zero time was measured immediately after laser pulse. Scale bars: 1 μm. (PDF 322 kb) [file 12915_2016_290_MOESM20_ESM.pdf]

WT

mamK D161A  
mamC::egfp

mCherry::mamK  
D161A

$\Delta$ mamK

M  
(kDa)

70

55

40

35

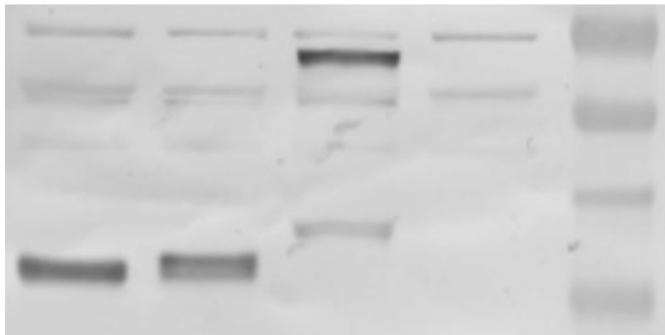

Supplement: Additional file 23: Figure S15. — MamK immunoblot. MamK protein presence was evaluated by western blot in the MSR WT, mamK D161A mamC-egfp, mCherry-mamK D161A and ∆mamK strains. MamK: ~37 KDa (lower arrowhead). mCherry-MamK: ~68 kDa (upper arrowhead). (PDF 50 kb) [file 12915_2016_290_MOESM23_ESM.pdf]
