# Supplementary material for: Segregation of prokaryotic magnetosomes organelles is driven by treadmilling of a dynamic actin-like MamK filament
Source: BMC Biol. 2016 Oct 12;14:88. doi: 10.1186/s12915-016-0290-1 (PMC5059902; doi:10.1186/s12915-016-0290-1)
Supplement: Additional file 16: Figure S10. — Half-time fluorescence recovery of MamK. (A) Half-time recovery of the fluorescence (t½) of mCherry-MamK in several strains is presented as bar chart highlighting significant differences. An unpaired Student’s t-test was carried out. * Significant: P = 0.01 to 0.05. ** Very significant P = 0.001 to 0.01. *** Extremely significant P < 0.001. ns: not significant. (B) Half-time recovery of the fluorescence (t½) box and whiskers plot for a better comparison of data distribution among strains. Error bars in “A” represent SEM and in “B” the 10–90 percentile. Lines inside the box specify the median. (C) Detailed half-time recovery of the fluorescence (t½) with SEM per strain. (PDF 273 kb) [file 12915_2016_290_MOESM16_ESM.pdf]

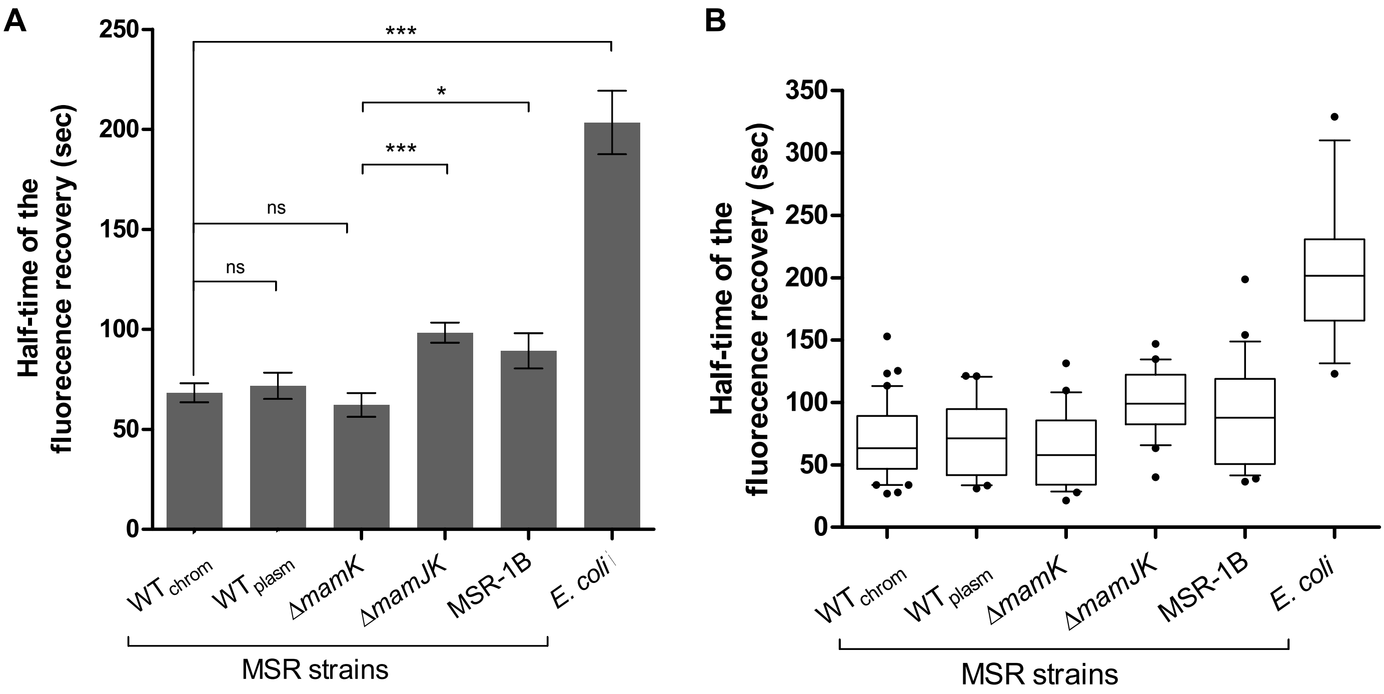

C

WT_{chrom}	$t_{1/2}$ = 68.3 ± 4.8 s	n= 40
WT_{plasm}	$t_{1/2}$ = 71.8 ± 6.6 s	n= 23
Δ*mamK*	$t_{1/2}$ = 62.2 ± 6.0 s	n= 26
Δ*mamJK*	$t_{1/2}$ = 98.4 ± 5.1 s	n= 26
MSR-1B	$t_{1/2}$ = 89.3 ± 8.8 s	n= 23
E. coli	$t_{1/2}$ = 203.4 ± 15.9 s	n=12
mamK D161A$_{chrom}$	t ½ = 12.5 ± 0.8 min	n=31
mamK D161A$_{plasm}$	t ½ = 10.8 ± 0.7 min	n=20
