# Supplementary material for: Segregation of prokaryotic magnetosomes organelles is driven by treadmilling of a dynamic actin-like MamK filament
Source: BMC Biol. 2016 Oct 12;14:88. doi: 10.1186/s12915-016-0290-1 (PMC5059902; doi:10.1186/s12915-016-0290-1)
Supplement: Additional file 18: Figure S11. — MamK filament treadmilling and dynamics. Bleach-marked mCherry-MamK filaments were used to follow the treadmilling in (Ai) MSR (chromosomal expression from the mamK locus. Fig. 5a) and (Bi) E. coli (expression from the lac promoter induced for 9 h, 1 mM IPTG). (Aii and Bii) Kymographs displaying fluorescence signal intensity (x-axis) of bleached MamK filaments over the time (y-axis) in the respective strains. The corresponding duplicated kymograph indicates the bleaching time/area (red box) and filament fluorescent signal progression (white dashed line). The bleach-marked filaments were followed for 5 min with imaging every 30 s. (C) Two kymographs of the red photoconverted Dendra2-MamK signal (x-axis) over time (y-axis). The corresponding duplicated kymograph below indicates the photoconversion time/area (red box) and MamK filament fluorescent signal appearance/progression from the cell pole (red dashed line). Right panel: cell shown in Fig. 7bi. (D) Quantification of photoconverted Dendra2-MamKD161A signal in the WT strain (n = 16). (E) Photoconversion of Dendra2-MamK (n = 31) or (F) Dendra2-MamK E143A (n = 39) expressed from the tetracycline-inducible promoter (Ptet), 6 h induced. Green channel: filament prior to photoconversion. Red channel: photoconverted protein after a 405 nm laser pulse. White dashed circles: photoconverted areas. Arrowhead indicates appearance of MamK signal at the cell pole. White dashed lines act as reference point. Red dashed lines: filament growth progression. (G) Dot plot of MamK filament treadmilling speed distribution in several strains, quantified from mCherry-MamK photobleaching data. Scale bars: 1 μm. (H) Detail of MamK filament treadmilling speeds. (I) Correlation plot of MamK filament growth speed versus the corresponding half-time fluorescence recovery. A linear regression (r 2 = 0.8275) shows negative correlation between speed and fluorescence recovery. Pearson correlation coefficient (r) shows a significant (P = 0.01 to 0.05) [file 12915_2016_290_MOESM18_ESM.pdf]

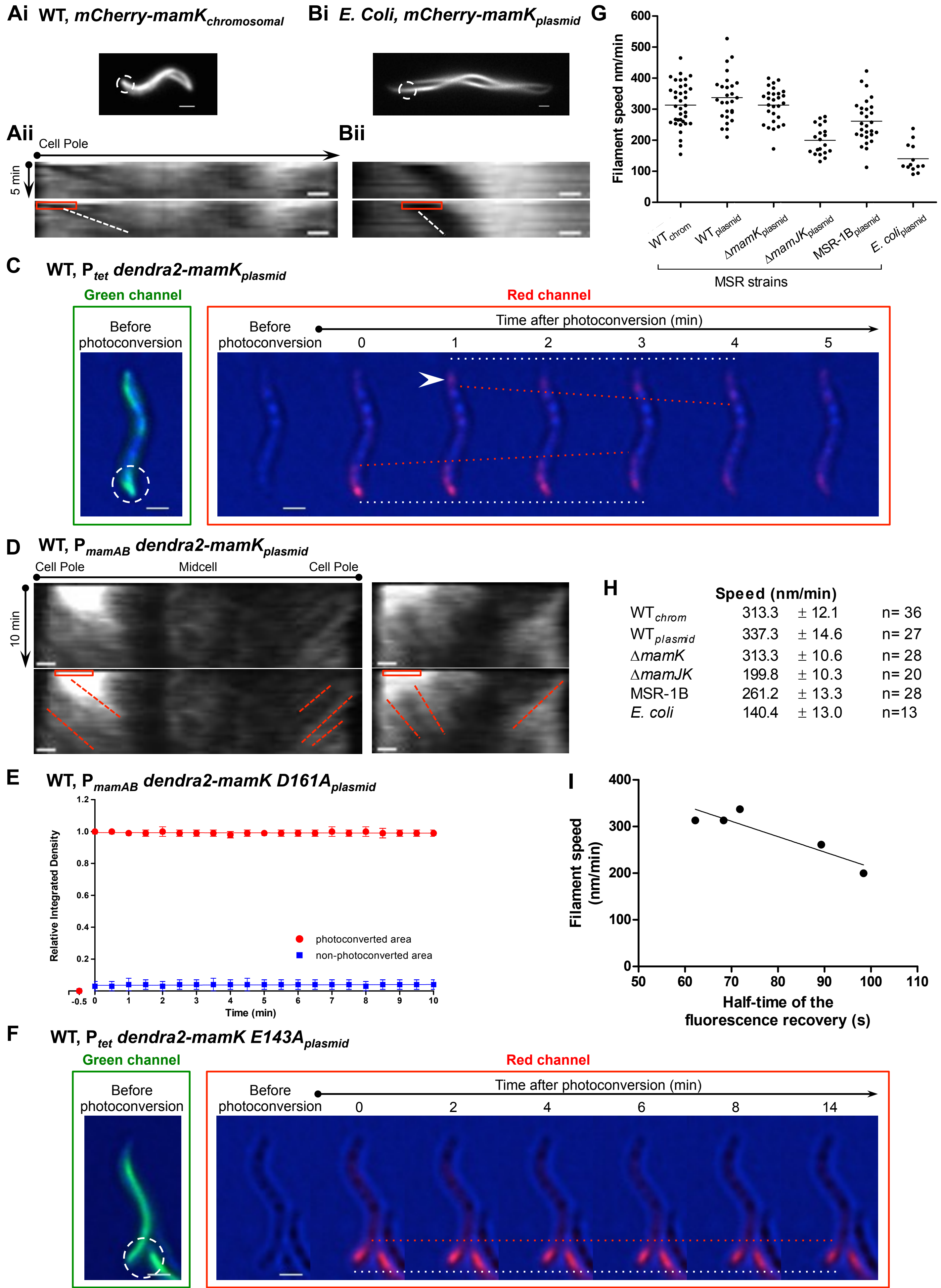
Ai WT, mCherry-mamK chromosomal
Bi E. Coli, mCherry-mamK plasmid
G
Aii
Cell Pole
5 min
Bii
Filament speed nm/min
600
500
400
300
200
100
0
WT chrom
WT plasmid
ΔmamK plasmid
ΔmamJK plasmid
MSR-1B plasmid
E. coli plasmid
MSR strains
C WT, P tet dendra2-mamK plasmid
Green channel
Red channel
Before photoconversion
Time after photoconversion (min)
0
1
2
3
4
5
D WT, P mamAB dendra2-mamK plasmid
Cell Pole
Midcell
Cell Pole
10 min
H
Speed (nm/min)
WT chrom 313.3 ± 12.1 n= 36
WT plasmid 337.3 ± 14.6 n= 27
ΔmamK 313.3 ± 10.6 n= 28
ΔmamJK 199.8 ± 10.3 n= 20
MSR-1B 261.2 ± 13.3 n= 28
E. coli 140.4 ± 13.0 n=13
E WT, P mamAB dendra2-mamK D161A plasmid
Relative Integrated Density
1.2
1.0
0.8
0.6
0.4
0.2
-0.5
Time (min)
photoconverted area
non-photoconverted area
I
Filament speed (nm/min)
400
300
200
100
0
50
60
70
80
90
100
110
Half-time of the fluorescence recovery (s)
F WT, P tet dendra2-mamK E143A plasmid
Green channel
Red channel
Before photoconversion
Time after photoconversion (min)
0
2
4
6
8
14
