# Supplementary material for: Segregation of prokaryotic magnetosomes organelles is driven by treadmilling of a dynamic actin-like MamK filament
Source: BMC Biol. 2016 Oct 12;14:88. doi: 10.1186/s12915-016-0290-1 (PMC5059902; doi:10.1186/s12915-016-0290-1)
Supplement: Additional file 19: Figure S12. — MamJ dynamics. (A) Photobleaching of MamJ-mCherry co-expressing mamK and evaluation of the fluorescence recovery in ∆mamJK. The right panel shows the quantification of the MamJ-mCherry fluorescence recovery over the time. Zero time was measured immediately after laser pulse. Half-time recovery of the fluorescence is presented as t½ in the plot. (B) Photoconversion of Dendra2 fused to MamJ in MSR wildtype. Arrow indicates MamJ signal progression. Similar behavior was observed in other analyzed cells (n = 22). (C) Photoconversion of MamJ-Dendra2 (n = 10) co-expressed with mamK in ∆mamJK. Arrow indicates appearance and progression of MamJ signal at the cell pole. (D) Photobleaching of MamJ-mCherry co-expressing mamK D161A under the control of the tetracycline-inducible promoter (Ptet) (24 h induced) and evaluation of the fluorescence recovery in MSR ∆mamK and (E) ∆mamJK strains. The plots show quantification of the MamJ-mCherry fluorescence recovery over the time. Zero time was measured immediately after laser pulse. Half-time recovery of the fluorescence is presented as t½ in the plot. (F) Expression of mamJ-dendra2 co-expressing mamK D161A (n = 15) from the tetracycline-inducible promoter (Ptet) (24 h induced) and posterior photoconversion in MSR ∆mamJK strain. Green channel displays the filament prior to photoconversion. Red channel shows photoconverted protein after a laser line 405 nm pulse application. (PDF 573 kb) [file 12915_2016_290_MOESM19_ESM.pdf]

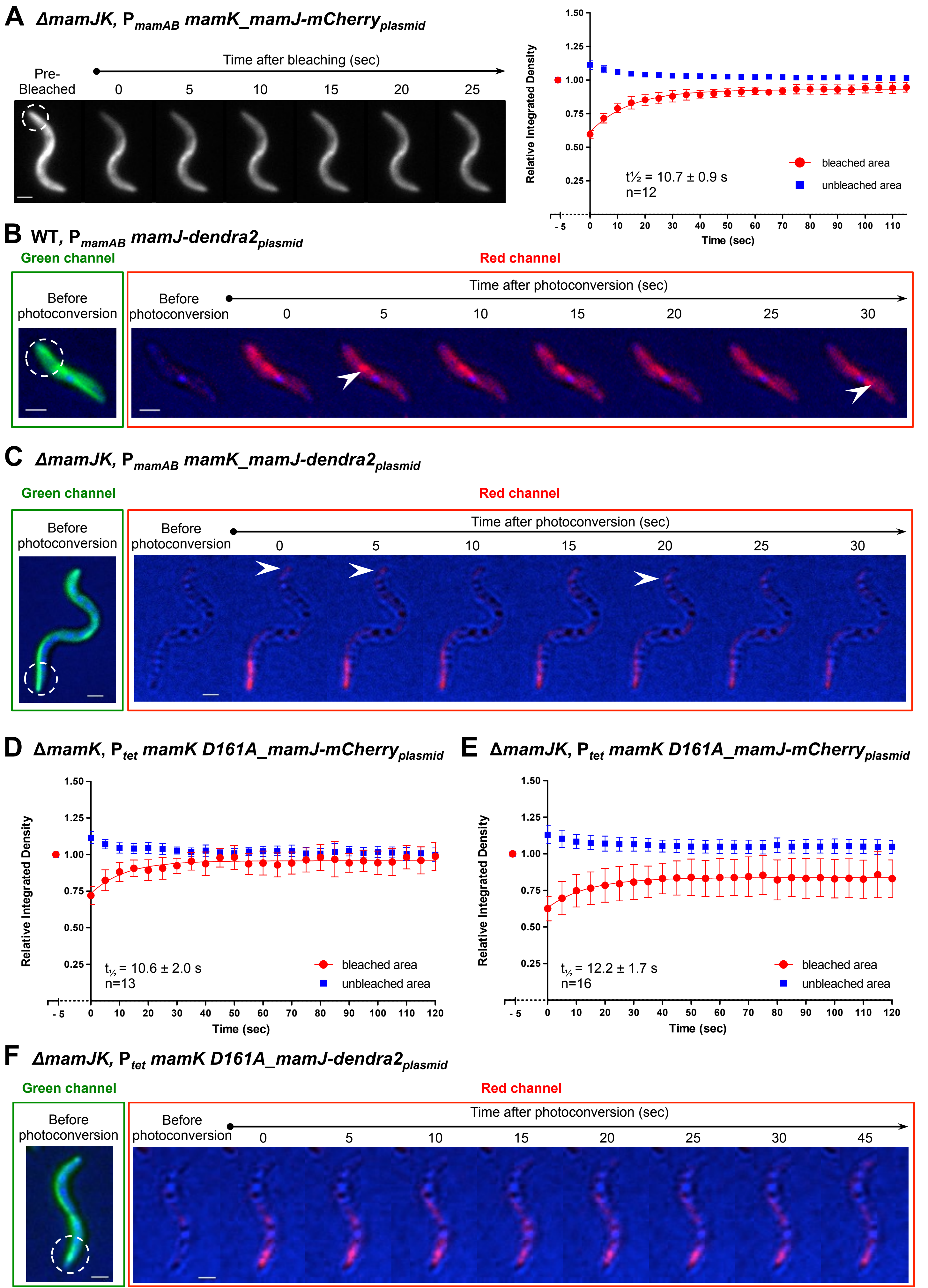

A
ΔmamJK, P_{mamAB} mamK_mamJ-mCherry$_{plasmid}$
Pre-Bleached
Time after bleaching (sec)
0 5 10 15 20 25
Relative Integrated Density
Time (sec)
t½ = 10.7 ± 0.9 s
n=12
bleached area
unbleached area
B
WT, P_{mamAB} mamJ-dendra2$_{plasmid}$
Green channel
Red channel
Before photoconversion
Time after photoconversion (sec)
0 5 10 15 20 25 30
C
ΔmamJK, P_{mamAB} mamK_mamJ-dendra2$_{plasmid}$
Green channel
Red channel
Before photoconversion
Time after photoconversion (sec)
0 5 10 15 20 25 30
D
ΔmamK, P_{tet} mamK D161A_mamJ-mCherry$_{plasmid}$
Relative Integrated Density
Time (sec)
$t_{½}$ = 10.6 ± 2.0 s
n=13
bleached area
unbleached area
E
ΔmamJK, P_{tet} mamK D161A_mamJ-mCherry$_{plasmid}$
Relative Integrated Density
Time (sec)
$t_{½}$ = 12.2 ± 1.7 s
n=16
bleached area
unbleached area
F
ΔmamJK, P_{tet} mamK D161A_mamJ-dendra2$_{plasmid}$
Green channel
Red channel
Before photoconversion
Time after photoconversion (sec)
0 5 10 15 20 25 30 45
