# Supplementary material for: Segregation of prokaryotic magnetosomes organelles is driven by treadmilling of a dynamic actin-like MamK filament
Source: BMC Biol. 2016 Oct 12;14:88. doi: 10.1186/s12915-016-0290-1 (PMC5059902; doi:10.1186/s12915-016-0290-1)
Supplement: Additional file 21: Figure S14. — MamK and MamJ functional complementation. (A) ∆mamJK cells forming clustered magnetosomes were used for evaluation of functionality of the constructs. (B) ∆mamJK strain co-expressing mamK and mamJ-mCherry from a replicative plasmid. (C) ∆mamJK strain expressing mamJ-dendra2 from a replicative plasmid. (D) ∆mamJK strain co-expressing mamK and mamJ-dendra2 from a replicative plasmid. (E) ∆mamJK strain co-expressing mamK D161A together with either mamJ-mCherry or (F) mamJ-dendra2 from a replicative plasmid under the control of the tetracycline-inducible promoter (Ptet) (24 h induced). Reconstitution of linear magnetosome chains (MCs) indicated successful gene expression and complementation of the phenotype. Notably, in (E) and (F), the mamK D161A phenotype of MC mispartitioning, polar localization and segmented chains were also observed in the complemented strains devoid of the mamK gene. Scale bars: 1 μm. (PDF 3422 kb) [file 12915_2016_290_MOESM21_ESM.pdf]

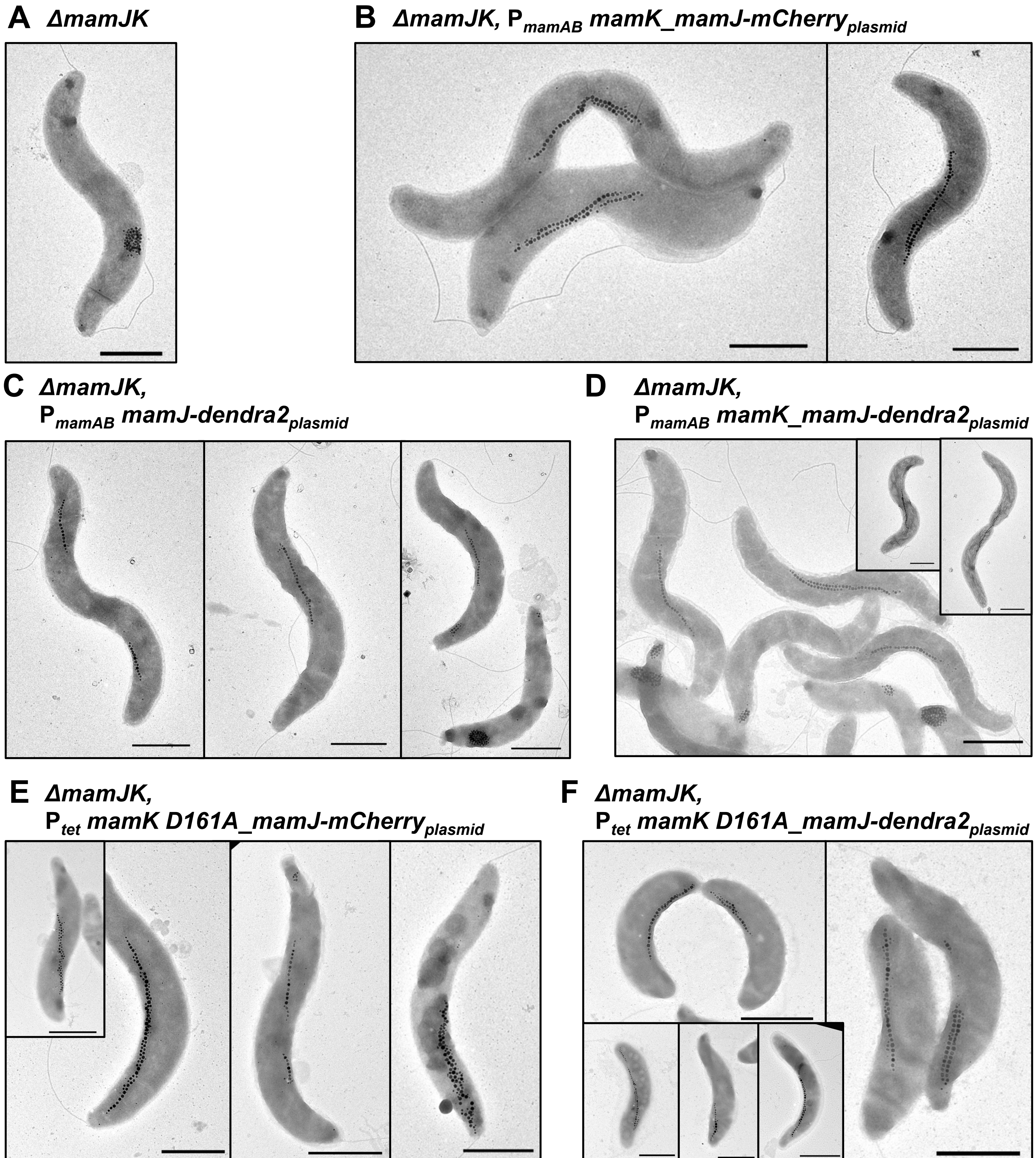
A ΔmamJK
B ΔmamJK, $P_{mamAB}$ mamK_mamJ-$mCherry_{plasmid}$
C ΔmamJK, $P_{mamAB}$ mamJ-$dendra2_{plasmid}$
D ΔmamJK, $P_{mamAB}$ mamK_mamJ-$dendra2_{plasmid}$
E ΔmamJK, $P_{tet}$ mamK D161A_mamJ-$mCherry_{plasmid}$
F ΔmamJK, $P_{tet}$ mamK D161A_mamJ-$dendra2_{plasmid}$
