# Supplementary material for: Segregation of prokaryotic magnetosomes organelles is driven by treadmilling of a dynamic actin-like MamK filament
Source: BMC Biol. 2016 Oct 12;14:88. doi: 10.1186/s12915-016-0290-1 (PMC5059902; doi:10.1186/s12915-016-0290-1)
Supplement: Additional file 22: — Supporting Methods. Text including details of methods that were not included in the main text (Additional file 23: Figure S15). (DOCX 190 kb) [file 12915_2016_290_MOESM22_ESM.docx]

**Additional File 22: Supporting Methods**

**Molecular and genetic techniques**

The draft genome sequence of *M. gryphiswaldense* (GenBank accession number CU459003) was used for oligonucleotides design. Oligonucleotides were purchased from Sigma-Aldrich. All constructs were sequenced on an ABI 3700 capillary sequencer (Applied Biosystems), utilizing BigDye Terminator v3.1. Sequence data were analyzed with CLC Main Workbench Software (Qiagen).

Plasmids construction

Plasmids were constructed by amplifying the DNA fragments of interest with the Phusion High Fidelity DNA Polymerase (Thermo Scientific). Plasmid description and oligonucleotides are listed in Table A and Table B, respectively (see below). For details of the in-frame insertion or deletion mechanism with the pORFM-*galK* plasmid refer to Raschdorf *et al.* (2014) [[1](#_ENREF_1)]. All plasmids were introduced into *M. gryphiswaldense* by means of conjugation.

Plasmid **pMT003** (in-frame deletion of *mamK*) was constructed to delete the *mamK* gene in the Δ*mamJ* background. Since the *mamK* gene is immediately downstream of *mamJ*, the upstream region of the plasmid should correspond to that of the mutant lacking *mamJ* gene. Then, about 1.5 kb of the *mamK* up- and downstream region were amplified from Δ*mamJ* genomic DNA using the primer pairs oMTN012-013 and oMTN014-015, respectively. Once the fusion of these regions was performed by overlap PCR, the fused fragments were cloned into pORFM-*galK* between the *Sal*I-*Not*I restriction sites. This vector was used to create the in-frame deletion of *mamK* generating the strain MT002 by homologous recombination.

Plasmid **pMT004** allows the in-frame insertion of the *mamK* *D161A* point mutation. The *mamK* gene was amplified from gDNA with the primer pair oMTN020-021 and served as a template for site-directed mutagenesis. Thus, *mamK* was mutated into *mamK* *D161A* by using the primer pair oMTN020-021 together with a third oligonucleotide (oMTN023) with an internal mismatch to direct the mutation where the codon GAT for aspartic acid (D) was replaced with GCC, a codon for an alanine (A). The mutagenized fragments were cloned into pORFM‑GalK with the *Sal*I-*Not*I restriction sites and sequenced to confirm correctness of the insert, and subsequently introduced by conjugation into several strains of *M. gryphiswaldense* to generate strains MT007 and MT008 by homologous recombination. MamK, MamK^D161A^ and mCherry-MamK^D161A^ protein presence was evaluated by immunoblot (Additional File 23: Figure S15).

To create **pMT009**, the plasmid pFM244 (with a constitutive P*_mamAB_* promoter from the operon *mamAB*) was digested with *Nde*I-*Bam*HI enzymes. The fragment *mCherry-mamK* was amplified from gDNA from the strain FM022 with primers oMTN044-045, identically digested and ligated into pFM244.

To construct **pMT010**, the *mCherry*-*mamK D161A* fragment was amplified with the primers oMTN044-045 using gDNA from strain MT008 as template, and further *Nde*I-*Bam*HI digested and cloned into pFM244.

For creation of **pMT062**, an overlapped PCR of *dendra2* and *mamK* was performed. The fragment *dendra2* was amplified with primers oMTN184-185, while *mamK* with oMTN186-045. These PCR fragments serve as template for the overlapped PCR using the primers oMTN184-045 to generate the *dendra2*-*mamK* fusion, which was *Nde*I-*Bam*HI digested and cloned into the identically digested pMT009.

**pMT063** was created by amplifying the *mamK D161A* gene with primers oMTN186-045 using pMT010 as template, the fragment as well as pMT062 were *Nhe*I-*Bam*HI digested and ligated.

To generate **pMT065**, the fragment *dendra2-mamK* was amplified with oMTN186-045 using pMT062 as template and further cloned into the vector pAP160 under the control of a tetracycline-inducible promoter (P*_tet_*) using the restriction sites *Nde*I-*Bam*HI.

For construction of **pMT067**, the fragment *mamK* was amplified with oMTN187-045. Then, *mamK* served as template for mutagenesis into *mamK* *E143A* by using the primer pair oMTN187-045 together with a third oligonucleotide (oMTN100) with an internal mismatch to direct the mutation where the codon GAC for glutamic acid (E) was replaced with GCC, a codon for an alanine (A). The mutated fragment *mamK* *E143A* was *Nhe*I-*Bam*HI digested and ligated into the identically digested pMT065.

Annealing two 5’-phosphorilated oligonucleotides oMTN224-225 generated an α-helix linker with a multiple cloning site before (*Nde*I-K*pn*I-*Eco*RI) and after (*Nhe*I-*Hind*III-*Bam*HI). The annealed oligonucleotides having complementary overhangs to *Nde*I-*Bam*HI were cloned into the pMT009 vector *Nde*I-*Bam*HI digested, producing the plasmid **pMT080**.

Vector **pMT081** was created by amplifying *dendra2* gene with the primers oMTN270-216 and cloned into pMT080 between the restriction sites *Hind*III-*Bam*HI.

To construct **pMT082**, the fragment *mamJ* was amplified with the oligonucleotides oMTN213-229 and further cloned into pMT081 between the restriction sites *Nde*I-*Eco*RI.

For creation of the **pMT083** vector, *mamK* and *mamJ* were amplified with oMTN040-271 and oMTN272-229, respectively. Afterwards, the fusion of both fragments (with the 5’ *mamC* intergenic region as spacer) was performed by overlapped PCR using *mamK* and *mamJ* fragments as templates and the oligonucleotides oMTN040-229. The *mamK-mamJ* insert as well as pMT081 vector were *Nde*I-*Eco*RI digested and ligated.

The plasmid **pMT085** was made by amplification of *mCherry* gene with oligonucleotides oMTN230-239 and cloning into pMT080 between *Nhe*I-*Bam*HI restriction sites.

Plasmid **pMT086** was generated by the amplification of *mamK-mamJ* with the primers oMTN040-229 and using the vector pMT083 as template. Next, the *mamK-mamJ* insert and pMT085 were *Nde*I-*Eco*RI digested and ligated.

Finally, to construct the vector **pMT090**, *mamK D161A* and *mamJ-mCherry* were amplified with oMTN040-271 and oMTN272-239, respectively. Afterwards, the fusion of *mamK D161A* and *mamJ-mCherry* was performed by overlapped PCR using *mamK D161A* and *mamJ-mCherry* fragments as templates and the oligonucleotides oMTN040-239. The *mamK D161A_mamJ-mCherry* fused fragment as well as pSB7 vector were *Nde*I-*Bam*HI digested and ligated.

To create the vector **pMT091**, the *dendra2* gene was amplified with the primer pairs oMTN270-216 and cloned into pMT090 using the restriction sites *Hind*III-*Bam*HI.

**Magnetosome chain mean-squared displacement, diffusion and velocity determination**

MSD was determined using consecutive localizations for each track with a total of N steps and calculated as:

$$MSD =\frac{1}{N-n}\sum_{i=1}^{N-n} \left[ {(x_{n+i}-x_{i})}^{2}+ {(y_{n+i}-y_{i})}^{2} \right]$$

MSD values were plotted over a range of lag times by calculating displacements over multiple frames. The apparent diffusion coefficient (D*) was obtained using the MSD data:

$$D* =\frac{MSD}{2d \tau}$$

Where *d* is the number of dimensions (*x* and *y*) and $\tau$ is the time interval as previously shown [[2](#_ENREF_2)]. MC velocity (V_MC_) was determined from the calculated displacement based on the coordinates (*x, y*) using a $\tau$ of 10 min.

**Induction of *de novo* magnetite crystal formation**

Briefly, in order to create non-magnetic cells, i.e., lacking magnetosomes, cells were iron starved in 6-well plates with LIM medium (containing 20 µM 2,2’ dypiridyl) in a microaerobic environment without agitation at 30ºC. For induction of magnetite biomineralization 100 µM Fe(III)-citrate was supplemented to cells, which were passaged at least 4x in LIM [[3](#_ENREF_3), [4](#_ENREF_4)]. Samples were withdrawn and fixed every 10 min during the first hour, every 30 min during the following 8 h, and every 24 h for the next 2 days. The O.D._565nm_ and magnetic response (C_mag_) [[5](#_ENREF_5)] were determined after fixation.

**Photokinetic analysis**

The specific settings used for each protein fusion during photokinetics experiments (such as FRAP and Photoconversion) was as follows:

FRAP

FRAP assays were carried out by photobleaching a small area of a cell and further imaging at various time intervals for MamK and MamJ fused to diverse fluorophores and upon the following conditions:

mCherry-MamK: mCherry filter set, 32% SSI with 150 ms exposure. Bleaching: 561 nm laser line (50 mW) at 10% power, 70% of laser in TIRF mode (only to decrease laser power as TIRF imaging was not performed) and a single pulse for 4 ms. Cell were imaged every 30 s.

MamJ-EGFP: FITC filter set, 32% SSI with 500 ms exposure. Bleaching: 488 nm laser line (100 mW) at 10% power, 65% of laser in TIRF mode and three iterations for 15 ms. Cell were imaged every 4 s.

MamJ-mCherry. mCherry filter set, 10% SSI with 250 ms exposure. Bleaching: 561 nm laser line (50 mW) at 100% power, 75% of laser in TIRF mode and a two iterations for 8 ms. Cell were imaged every 5 s.

The laser event was always placed after the first image. Half-time fluorescence recoveries (t _½_) were calculated independently per each bleached cell and averaged in order to obtain the SEM for the cells community. Additionally, each FRAP related plot show the SD per each time point.

Photoconversion

The monomeric Green-to-Red photoconvertable Dendra2 protein was used for qualitative evaluation of intracellular protein dynamics in MSR. Non-activated Dendra2 possesses excitation-emission maxima at 486 and 505 nm and can be visualized with the FITC filter set. Upon a 405 nm laser pulse application, Dendra2 can be activated or photoconverted to a red fluorescent state of an excitation-emission maxima at 558 and 575 nm, respectively, and imaged with the TRITC filter set.

Dendra2-MamK. Before photoconversion: FITC filter set, 10% SSI with 150 ms exposure was used. Photoconversion: 405 nm laser line (100 mW), 10% power, 70% of laser in TIRF mode and a single pulse for 4 ms. For imaging after photoconversion: TRITC filter set, 32% SSI with 500 ms exposure. Cell were imaged every 30 s.

MamJ-Dendra2. Before photoconversion: FITC filter set, 10% (or 5% for the construct pMT084 co-expressing *mamK*) SSI with 500 ms exposure was used. Photoconversion: 405 nm laser line (100 mW), 10% power, 70% of laser in TIRF mode and a single pulse for 4 ms. For imaging after photoconversion: TRITC filter set, 32% SSI with 500 ms exposure. Cells were imaged every 5 s. The laser event was always placed after the first image.

Table A. Bacterial strains created and used in this work

| Strain* | Genotype or characteristics | Reference or source |
| --- | --- | --- |
| *M. gryphiswaldense* |  |  |
| MSR-1 R3/S1 | Wild-type (Rif^R^, Sm^R^). Host for gene expression and localization studies. Refered to as MSR WT. | Schultheiss and Schüler, 2003 [[6](#_ENREF_6)] |
| Δ*mamJ* | Δ*mamJ* | Scheffel et al, 2006 [[3](#_ENREF_3)] |
| Δ*mamK* | Δ*mamK* | Katzmann et al, 2010 [[7](#_ENREF_7)] |
| MSR-1B | Spontaneous non-magnetic mutant | Schübbe et al, 2003 [[8](#_ENREF_8)] |
| FM021 | *mamC-egfp* | Raschdorf et al, 2014 [[1](#_ENREF_1)] |
| FM022 | *mCherry-mamK* | Raschdorf et al, 2014 [[1](#_ENREF_1)] |
| MT002 | Δ*mamJK* | This work |
| MT007 | *mamK* *D161A*, *mamC-egfp* | This work |
| MT008 | *mCherry-mamK D161A* | This work |
| MT009 | MSR WT, conjugated with Tet-pBam_MamJ-EGFP, Tc^R^ | This work |
| MT010 | MSR WT, conjugated with pMT090, Km^R^ | This work |
| MT011 | Δ*mamK*, conjugated with pMT090, Km^R^ | This work |
| MT012 | Δ*mamJK*, conjugated with pMT090, Km^R^ | This work |
| MT013 | Δ*mamJK*, conjugated with pMT091, Km^R^ | This work |
| MT014 | MSR WT, conjugated with pMT091, Km^R^ | This work |
| eMT001 | MSR WT, conjugated with pMT009, Km^R^ | This work |
| eMT002 | MSR WT, conjugated with pMT010, Km^R^ | This work |
| eMT003 | Δ*mamK*, conjugated with pMT009, Km^R^ | This work |
| eMT004 | Δ*mamJK*, conjugated with pMT009, Km^R^ | This work |
| eMT005 | MSR-1B, conjugated with pMT009, Km^R^ | This work |
| eMT006 | MSR WT, conjugated with pMT062, Km^R^ | This work |
| eMT007 | MSR WT, conjugated with pMT063, Km^R^ | This work |
| eMT008 | MSR WT, conjugated with pMT065, Km^R^ | This work |
| eMT009 | MSR WT, conjugated with pMT067, Km^R^ | This work |
| eMT010 | MSR WT, conjugated with pMT082, Km^R^ | This work |
| eMT011 | MSR WT, conjugated with pMT084, Km^R^ | This work |
| eMT012 | Δ*mamJK*, conjugated with pMT084, Km^R^ | This work |
| eMT013 | MSR WT, conjugated with pMT086, Km^R^ | This work |
| eMT014 | Δ*mamJK*, conjugated with pMT086, Km^R^ | This work |
| *E. coli* |  |  |
| DH5α | Host for cloning. F^-^ φ80*lac*ZΔM15 Δ(*lac*ZYA-*arg*F) U169 *rec*A1 *end*A1 *hsd*R17 (rK-, mK+) *pho*A *sup*E44 λ-*thi*-1 *gyr*A96 *rel*A1 | Invitrogen |
| BW29427 | *thrB1004 pro thi rpsL hsdS lacZ*ΔM15 *RP4-1360* Δ(*araBAD*)*567* Δ*dapA1341*::[*erm pir* (*wt*)] | Datsenko and Wanner (unpublished) |
| eMT015 | DH5α, transformed with pMT009, Km^R^ | This work |

* MTN strains: chromosomally stably modified. eMTN strains: transformed with a replicative vector.

Table B. Plasmids created and used in this study

| Plasmid | Relevant characteristics | Reference or source |
| --- | --- | --- |
| pORFM-GalK-MCS | Integrative backbone vector for in-frame gene deletion. oriT, P_tet_-galK, Km^R^, Tc^R^ | Raschdorf et al, 2014 [[1](#_ENREF_1)] |
| pBBR1-MCS2 | Replicative backbone vector for *in trans*  gene expression in MSR. oriT, mob, Km^R^ | Kovach et al, 1994 [[9](#_ENREF_9)] |
| Tet-pBAM_MamJ-EGFP | pBAM1 derivative (Tc^R^, Ap^R^, γR6K origin of replication, oriT, Tn5 vector). *mamJ-egfp* under control of P*_mamDC_* promoter | Kolinko et al, 2014 [[10](#_ENREF_10)] |
| pSB7 | pBAM1 derivative (Km^R^, γR6K origin of replication, oriT, Tn5 vector). *egfp* under control of P*_tet_* promoter | Borg et al, 2014 [[11](#_ENREF_11)] |
| pFM244 | pBBR1-MCS2 based vector. P*_mamAB_*;Km^R^ | F. Müller, (unpublished) |
| pAP160 | pBBR-MCS2, with P*_tet_*, *egfp*,  terminator-fragment, P*_Neo_*-TetR; Km^R^ | A. Pollity (unpublished) |
| pMT003 | pORFM-GalK derivative,  *mamK* deletion into Δ*mamJ* strain | This work |
| pMT004 | pORFM-GalK derivative,  *mamK* *D161A* mutation | This work |
| pMT009 | pFM244 derivative, P*_mamAB_*-*mCherry-mamK* | This work |
| pMT010 | pFM244 derivative, P*_mamAB_*-*mCherry-mamK D161A* | This work |
| pMT062 | pMT009 derivative, P*_mamAB_*-*dendra2-mamK* | This work |
| pMT063 | pMT062 derivative, P*_mamAB_*-*dendra2-mamK D161A* | This work |
| pMT065 | pAP160 derivative, P*_tet_*-*dendra2-mamK* | This work |
| pMT067 | pMT065 derivative, P*_tet_*-*dendra2-mamK E143A* | This work |
| pMT080 | pMT009 derivative, P*_mamAB_*-*α-helix* | This work |
| pMT081 | pMT080 derivative, P*_mamAB_*-*α-helix- dendra2* | This work |
| pMT082 | pMT081 derivative, P*_mamAB_*-*mamJ-dendra2* | This work |
| pMT083 | pMT081 derivative, P*_mamAB_*-*mamK_mamJ-dendra2* | This work |
| pMT085 | pMT080 derivative, P*_mamAB_*-*α-helix-mCherry* | This work |
| pMT086 | pMT083 derivative, P*_mamAB_*-*mamK_mamJ-mCherry* | This work |
| pMT090 | pSB7 derivative, P*_tet_*-*mamK D161A_mamJ-mCherry* | This work |
| pMT091 | pMT090 derivative, P*_tet_*-*mamK D161A_mamJ-dendra2* | This work |

Table C. DNA Oligonucleotides used in this study

| Primer | Sequence 5’→3’ | Remarks |
| --- | --- | --- |
| oMTN012 | AGACTA**GTCGAC**GAGCTGGGGGCGCCTATCGCTTTTCCCA | ***Sal*I**, overhang |
| oMTN013 | tcaaaagtcggcctgttcttgGCCTGGCCTTCACCTTCACTC | Lowercases: complementary region to oMTN014 |
| oMTN014 | caagaacaggccgacttttgaTGGTTGCCGGGGCGCTCTGCGGC | Lowercases: complementary region to oMTN013 |
| oMTN015 | AGACTA**GCGGCCGC**CATGCCCACATTGACGCCGATGGAAAC | ***Not*I**, overhang |
| oMTN016 | CTACTCCTGTTGCCGGGGGGGGTAA |  |
| oMTN017 | CCCGATTTGGGCCACTGATAATGCTTGC |  |
| oMTN018 | TTTGGCCAACCGATGATGCCCATTGCCG |  |
| oMTN019 | GGCTGTTAGTCTCAATGGCGACACAGCG |  |
| oMTN020 | AGACTA**GTCGAC**GGATTGATCTGTTAGCACGCGAACGGAGTGACAA | ***Sal*I**, overhang |
| oMTN021 | AGACTA**GCGGCCGC**GCCACGCATCTTCGCCAACCAAAATGACATCC | ***Not*I**, overhang |
| oMTN023 | CCATCATTGTCG***cc***ATCGGCGCCGGGAC | ***nucleotide exchange*** |
| oMTN024 | ATCGGCGTGCCGGCCCGAGCGTCGGGAGC | - |
| oMTN040 | AGACTA**CATATG**AGTGAAGGTGAAGGCCAGGCC | ***Nde*I**, overhang |
| oMTN044 | AGACTA**CATATG**GTGAGCAAGGGCGAGGAGGATAAC | ***Nde*I**, overhang |
| oMTN045 | AGACTA**GGATCC**TCACTGACCGGAAACGTCACCAAGC | ***Bam*HI**, overhang |
| oMTN100 | CTGGTGGTATCCG***cg***CCGTTCATG | ***nucleotide exchange*** |
| oMTN184 | AGACTA**CATATG**AACACCCCGGGAATTAACCTGATCAAG | ***Nde*I**, overhang |
| oMTN185 | agctcgagatcttaaggtaccCCACACCTGGCTGGGCAGGG | Lowercases: complementary region to oMTN186 |
| oMTN186 | gcggccgccgatcct**GCTAGC**ATGAGTGAAGGTGAAGGCCAGGCC | Lowercases: complementary region to oMTN185. ***Nhe*I** |
| oMTN187 | AGACTA**GCTAGC**ATGAGTGAAGGTGAAGGCCAGGCC | ***Nhe*I**, overhang |
| oMTN213 | **CATATG**GCAAAAAACCGGCGTGATCGCGG | ***Nde*I** |
| oMTN216 | **GGATCC**TTACCACACCTGGCTGGGCAGG | ***Bam*HI** |
| oMTN229 | AGACTA**GAATTC**TTTATTCTTATCTTCAGCATCACATTTCGGCGATG | ***Eco*RI**, overhang |
| oMTN230 | AGACTA**GCTAGC**ATGGTGAGCAAGGGCGAGGAGGATAAC | ***Nhe*I**, overhang |
| oMTN239 | AGACTA**GGATCCTTA**CTTGTACAGCTCGTCCATGCCG | ***Bam*HI**, overhang |
| oMTN270 | AGACTA**AAGCTT**ATGAACACCCCGGGAATTAACCTGATCAA | ***Hind*III**, overhang |
| oMTN271 | cgctgttgtccttaattcaagggtcagTCACTGACCGGAAACGTCACCAAGCTG | Lowercases represent the *mamC* intergenic region and the complementary region to oMTN272 |
| oMTN272 | ctgacccttgaattaaggacaacagcgATGGCAAAAAACCGGCGTGATCGCGG | Lowercases represent the *mamC* intergenic region and the to oMTN271 |
| oFM280a | CTGCCACTCATCGCAGTCTAGCTTGG | Raschdorf et al, 2014 [[1](#_ENREF_1)] |
| oFM281 | GGCTTTCTACGTGTTCCGCTTCCTTTAGC | Raschdorf et al, 2014 [[1](#_ENREF_1)] |

**Supplementary References**

1. Raschdorf O, Plitzko JM, Schüler D, Müller FD: **A tailored galK counterselection system for efficient markerless gene deletion and chromosomal tagging in *Magnetospirillum gryphiswaldense***. *Appl Environ Microbiol* 2014, **80**(14):4323-4330.

2. Uphoff S, Reyes-Lamothe R, Garza de Leon F, Sherratt DJ, Kapanidis AN: **Single-molecule DNA repair in live bacteria**. *Proc Natl Acad Sci USA* 2013, **110**(20):8063-8068.

3. Scheffel A, Gruska M, Faivre D, Linaroudis A, Graumann PL, Plitzko JM, Schüler D: **An acidic protein aligns magnetosomes along a filamentous structure in magnetotactic bacteria**. *Nature* 2006, **440**(7080):110-114.

4. Faivre D, Böttger LH, Matzanke BF, Schüler D: **Intracellular magnetite biomineralization in bacteria proceeds by a distinct pathway involving membrane-bound ferritin and an iron(II) species**. *Angew Chem Int Ed Engl* 2007, **46**(44):8495-8499.

5. Schüler D, Uhl R, Baeuerlein E: **A simple light scattering method to assay magnetism in *Magnetospirillum gryphiswaldense***. *FEMS Microbiol Lett* 1995, **132**:139-145.

6. Schultheiss D, Schüler D: **Development of a genetic system for *Magnetospirillum gryphiswaldense***. *Archives of Microbiology* 2003, **179**(2):89-94.

7. Katzmann E, Scheffel A, Gruska M, Plitzko JM, Schüler D: **Loss of the actin-like protein MamK has pleiotropic effects on magnetosome formation and chain assembly in *Magnetospirillum gryphiswaldense***. *Mol Microbiol* 2010, **77**(1):208-224.

8. Schübbe S, Kube M, Scheffel A, Wawer C, Heyen U, Meyerdierks A, Madkour MH, Mayer F, Reinhardt R, Schuler D: **Characterization of a Spontaneous Nonmagnetic Mutant of *Magnetospirillum gryphiswaldense* Reveals a Large Deletion Comprising a Putative Magnetosome Island**. *J Bacteriol* 2003, **185**(19):5779-5790.

9. Kovach M, Phillips R, Elzer P, Roop R, Peterson K: **pBBR1MCS: a broad-host-range cloning vector**. *BioTechniques* 1994, **16**(5):800-802.

10. Kolinko I, Lohsse A, Borg S, Raschdorf O, Jogler C, Tu Q, Posfai M, Tompa E, Plitzko JM, Brachmann A *et al*: **Biosynthesis of magnetic nanostructures in a foreign organism by transfer of bacterial magnetosome gene clusters**. *Nat Nanotechnol* 2014, **9**(3):193-197.

11. Borg S, Hofmann J, Pollithy A, Lang C, Schuler D: **New vectors for chromosomal integration enable high-level constitutive or inducible magnetosome expression of fusion proteins in *Magnetospirillum gryphiswaldense***. *Appl Environ Microbiol* 2014, **80**(8):2609-2616.
